# Deciphering 3'UTR Mediated Gene Regulation Using Interpretable Deep Representation Learning

**DOI:** 10.1002/advs.202407013

**Published:** 2024-08-19

**Authors:** Yuning Yang, Gen Li, Kuan Pang, Wuxinhao Cao, Zhaolei Zhang, Xiangtao Li

**Affiliations:** ^1^ School of Information Science and Technology Northeast Normal University Changchun Jilin 130117 China; ^2^ Donnelly Centre for Cellular and Biomolecular Research University of Toronto Toronto ON M5S 3E1 Canada; ^3^ Department of Computer Science University of Toronto Toronto ON M5S 3E1 Canada; ^4^ Department of Molecular Genetics University of Toronto Toronto ON M5S 3E1 Canada; ^5^ School of Artificial Intelligence Jilin University Changchun Jilin 130012 China

**Keywords:** 3'UTRs, deep representation learning, m6A modification preference, mRNA subcellular localization, RNA–RBP interaction

## Abstract

The 3' untranslated regions (3'UTRs) of messenger RNAs contain many important cis‐regulatory elements that are under functional and evolutionary constraints. It is hypothesized that these constraints are similar to grammars and syntaxes in human languages and can be modeled by advanced natural language techniques such as Transformers, which has been very effective in modeling complex protein sequence and structures. Here 3UTRBERT is described, which implements an attention‐based language model, i.e., Bidirectional Encoder Representations from Transformers (BERT). 3UTRBERT is pre‐trained on aggregated 3'UTR sequences of human mRNAs in a task‐agnostic manner; the pre‐trained model is then fine‐tuned for specific downstream tasks such as identifying RBP binding sites, m6A RNA modification sites, and predicting RNA sub‐cellular localizations. Benchmark results show that 3UTRBERT generally outperformed other contemporary methods in each of these tasks. More importantly, the self‐attention mechanism within 3UTRBERT allows direct visualization of the semantic relationship between sequence elements and effectively identifies regions with important regulatory potential. It is expected that 3UTRBERT model can serve as the foundational tool to analyze various sequence labeling tasks within the 3'UTR fields, thus enhancing the decipherability of post‐transcriptional regulatory mechanisms.

## Graphical abstract

The 3UTRBERT language model uses Transformer architectures to analyze 3'UTR regions of mRNAs. Pre‐trained on human 3'UTR sequences, it is fine‐tuned to identify RBP binding sites, m6A RNA modifications, and RNA sub‐cellular localizations. 3UTRBERT outperforms other methods, highlighting key functional regions and enhancing the understanding of post‐transcriptional regulatory mechanisms.

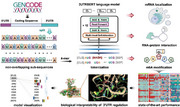

## 1 Introduction

The 3' untranslated regions (3'UTRs) of messenger RNAs (mRNAs) are critical in regulating gene and protein expression at the post‐transcriptional level.^[^ ^1^ ^]^ Cis‐regulatory elements located in the 3'UTRs are recognized and bound by trans‐acting factors such as RNA‐binding proteins (RBPs) and microRNAs, which can modulate mRNA modification, abundance, and mRNA subcellular localization.^[^ ^2^, ^3^, ^4^ ^]^ A number of computational methods have been developed to predict these cis‐regulatory elements, either by matching mRNA sequence with experimentally determined binding motifs, or by taking advantage of evolutionary conservation information, or a combination of both approaches. For example, by comparing closely related genome sequences, Kellis and Xie derived putative regulatory elements in yeast and mammals, respectively.^[^ ^5^, ^6^ ^]^ More sophisticated methods such as PhastCons and PhyloP incorporate phylogenetic information with hidden Markov models to identify genomic regions that are under evolutionary constraints, which have been widely used in the field.^[^ ^7^, ^8^ ^]^ Additionally, specialized computational tools were also developed for specific tasks such as RNA–RBP interaction,^[^ ^9^, ^10^ ^]^ m6A modification preference,^[^ ^11^ ^]^ and mRNA subcellular localization.^[^ ^12^ ^]^

In recent years, advances in deep learning (DL) have prompted researchers to develop DL‐based methods in the study of RNA regulation; these methods include Convolutional Neural Network (CNN),^[^ ^9^ ^]^ Recurrent Neural Network (RNN),^[^ ^13^ ^]^ or integration of both architectures.^[^ ^14^, ^15^ ^]^ For example, iDeepE combines local multi‐channel CNNs and a global CNN to predict RBP binding sites from sequences alone;^[^ ^16^ ^]^ DeepCLIP uses one‐hot RNA encoding to represent RBP binding sites and constructs a shallow neural network composed of CNN and LSTM layers.^[^ ^14^ ^]^ However, these models mainly address the linear sequential data, they are ineffective in learning the non‐Euclidean representational information of the RNA sequences. To compensate for this limitation, RPI‐Net employs a graph neural network (GNN)‐based framework and adds base‐pairing information to enhance prediction accuracy;^[^ ^17^ ^]^ Graphprot2 employs graph convolutional neural network (GCN) with RNA topological structure information to infer protein‐RNA binding preferences.^[^ ^18^ ^]^ Despite promising results, the natural binding patterns are potentially lost when sequences in converted to graph formats, and the graph neural networks are also limited to capture long‐range semantic dependencies. More importantly, the deep neural network architectures often suffer from difficulty to parallelize, gradient vanishing and reproducibility, causing bottlenecks in terms of prediction performance.

Recent breakthroughs in natural language processing (NLP) models have inspired the exploration of these sophisticated tools for studying biological sequences. The rationale behind these approaches is that biological sequences such as protein amino acid sequences or regulatory elements follow evolutionary or functional constraints that are similar to the evolution of syntax and gramma of human language so that NLP models can be applied to these sequences to derive the latent constraints or “grammars” hidden in the biological sequences.^[^ ^19^ ^]^ Among these modern NLP models, attention‐based Transformers and Bidirectional Encoder Representations from Transformers (BERT) were recently adapted to model biological sequences on specific tasks.^[^ ^20^, ^21^ ^]^ Ji et al. introduced DNABERT, a Transformer model pretrained on the whole human reference genome that enabled fine‐tuning for specific tasks, such as the annotation of promoters or splice sites.^[^ ^22^ ^]^ Brandes and colleagues developed a protein‐BERT model, which allowed them to predict protein properties such as secondary structure, stability and remote homology.^[^ ^23^ ^]^ Elnaggar and colleagues evaluated several auto‐regressive models and autoencoder language models, and described an embedding‐based model called ProtTrans, which established that NLP models can be useful in learning protein properties.^[^ ^24^ ^]^ Recently, NLP models were also successfully applied to transcriptome fields, e.g., RNABERT pretrains a Transformer module from non‐coding RNAs (ncRNAs) for structural alignment and family clustering tasks;^[^ ^25^ ^]^ Built upon this architecture, RNA‐FM enlarges the training samples and increases the number of attention heads to generate a more informative embedding for each nucleotide.^[^ ^26^ ^]^ Subsequently, with the success of NLP models in predicting the protein 3D structures,^[^ ^27^, ^28^ ^]^ RNA‐MSM also employs homologous sequence conservation information to predict RNA secondary structure and solvent accessibility.^[^ ^29^ ^]^ Besides these non‐coding region‐based language models, Rm‐LR incorporates a bilinear attention network pretrained by pre‐mRNA sequences to identify RNA modification sites.^[^ ^30^ ^]^ Similarly, Yamada et al. developed BERT‐RBP, a model fine‐tuned on the eCLIP‐seq datasets using DNABERT, which enables the identification of RNA–protein interactions.^[^ ^31^ ^]^ This convergence of NLP methodologies and multi‐omics signifies a promising frontier in bioinformatics, potentially transforming our understanding of complex biological systems.

Motivated by the success of language models on the study of proteins, we herein describe a new method, 3UTRBERT, which uses a language model to identify constrained regions in the 3'UTR of mRNAs. Language models applied on the protein sequences can reveal sequence and structure constraints, while language models on genomic DNAs can help identify potential transcriptional regulatory elements. We hypothesize that language models can be applied to mRNA sequences to help identify regulatory elements important at the post‐transcriptional level. To the best of our knowledge, there have not been any published studies on deploying Transformer or other NLP models on mRNA sequences. Existing transcript‐centric language models mainly focus on non‐coding RNA sequences and lack enough exploration of the crucial regions contributing to mRNA stability and post‐transcriptional regulation. Our method, 3UTRBERT, was pre‐trained on aggregated 3'UTR sequences of human mRNAs in a task‐agnostic manner; the pre‐trained model was then fine‐tuned for specific downstream tasks such as predicting RBP binding sites (41 RBPs covering multiple CLIP experiments), m6A RNA modification sites, or RNA sub‐cellular localizations. For each of these sub‐tasks, we benchmarked the performance of 3UTRBERT against state‐of‐the‐art methods. Our results showed that 3UTRBERT generally outperforms other methods for these tasks. More importantly, by analyzing the motif patterns and high‐attention regions extracted from the model, we can demonstrate the regions with potential regulatory functions in the sequences and visualize the learning integration process of effective information, which bridges the lack of interpretability among the existing language models.

## 2 Results

### 2.1 Overview of 3UTRBERT Design and Applications

**Figure 1:**
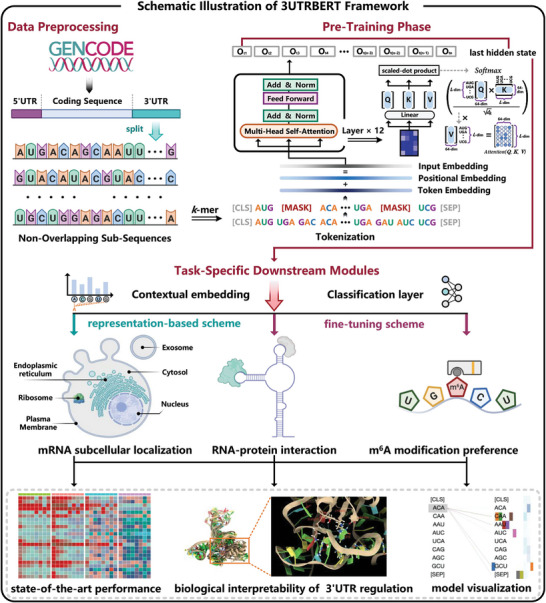
Schematic illustration of 3UTRBERT framework. 3UTRBERT is pre‐trained on the 3'UTR sequences of human genes using self‐supervised learning. Unlabeled mRNA sequences are segmented by sequential 3‐mer tokens and subsequently fed into a Transformer architecture to learn general‐purpose representations using a masked language model, i.e., reconstructing the masked tokens from other tokens thus learning the syntax and grammar hidden in the 3'UTRs. The pre‐trained model is then fine‐tuned on several downstream prediction tasks, including predicting RBP–RNA interactions, m6A modification sites and mRNA subcellular localization.

The top of **Figure** **1** shows that 3UTRBERT was first trained with unannotated RNA fragments using self‐attention‐based Transformer architecture. The correlations among token positions learned in the pre‐training stage were aggregated into the final hidden vector of the [CLS] token to provide general and transferable information. This flexibility allows the model to efficiently work with both long RNA sequences and short RNA fragments in downstream tasks (see Experimental Section). For classification problems involving longer sequences, we freeze the model parameters and extract the contextual embedding inside 3UTRBERT as the effective feature scheme for each base. Meanwhile, 3UTRBERT can also be directly fine‐tuned on binary labels of short‐region classification tasks in an end‐to‐end manner. We further demonstrated the flexible configurability and the effectiveness of such knowledge‐embedded framework for transcript labeling. 3UTRBERT compared favorably against the latest methods for predicting RBP–RNA interactions, m6A modifications, and mRNA subcellular localization (bottom half of Figure 1). In addition, the interpretable analysis implemented by self‐attention mechanism also allowed visualization of the semantic relationship among nucleotides.

### 2.2 3UTRBERT Improves Detection of RNA‐Protein Binding Sites

**Figure 2:**
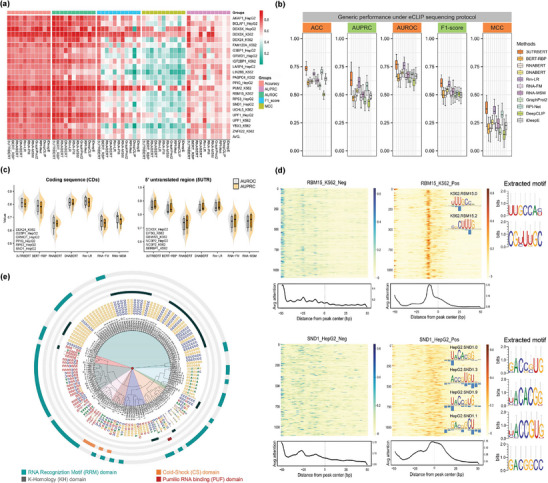
a) Performance comparisons between 3UTRBERT and other methods on predicting RBP–RNA interactions (22 RBPs under eCLIP protocol); prediction results are color coded and * represent a value greater than 0.75. b) The boxplot evaluated the performance of different methods in RBP‐generic strategy, which trained a model applicable to any RBPs. c) Violin plot of the overall AUROC and AUPRC scores of 3UTRBERT versus other language models on CDs and 5'UTRs. d) Landscape of attention scores surrounding RBP–RNA binding sites and the negative control sites; RBM15 in K562 cell line and SND1 in HepG2 cell line are shown as examples. e) Radial tree plot showing the congruent clustering of RBP‐binding motifs and RNA‐binding domains.

RNA‐binding proteins (RBPs) typically recognize specific sequence motifs facilitated by local RNA structure features on the mRNA transcripts.^[^ ^32^ ^]^ We hypothesize that the sequence windows with high attention scores were enriched with these RBP binding sites. As described in the Experimental Section, we downloaded and processed protein‐RNA crosslinking sites for 41 RBPs of different CLIP protocols and used these experimentally determined binding sites to fine‐tune the Transformer model to predict RBP binding sites. We next benchmarked the performance of 3UTRBERT with different types of state‐of‐the‐art computational methods: including CNN and RNN‐based model, graph neural network (GNN)‐based model, and Transformer‐based model (**Figure** **2**a,b). For our benchmark studies, we included iDeepE, DeepCLIP, RPI‐Net, GraphProt2, RNABERT, RNA‐FM, RNA‐MSM, BERT‐RBP, and Rm‐LR.

We used fivefold cross‐validation to test the performance of the predictive methods (please see Experimental Section for details). For each of CLIP‐seq datasets, all predictors were subjected to the same training, validation, and test sets. The following metrics were used to quantify the performance of predictions: 1) AUROC (Area Under the Receiver Operating Characteristic), 2) AUPRC (Area Under the Precision‐Recall Curve), 3) F1‐score (the harmonic mean between precision and recall), 4) MCC (Matthews Correlation Coefficient), and 5) ACC (accuracy). The formal definition of these metrics can be found in Note S1 (Supporting Information). We first conducted benchmark studies on 22 RBPs from eCLIP protocol using an RBP‐specific strategy, which involved modeling the RNA binding properties by constructing a separate model for each RBP. The comparison results for other predictors were reproduced using the default parameters as described in their original publications. Figure 2a shows that 3UTRBERT generally outperformed other methods (AUROC = 0.836, AUPRC = 0.695, F1 = 0.750, MCC = 0.503, and ACC = 0.785), while the second‐best results were AUROC = 0.809 (by Rm‐LR), AUPRC = 0.671 (by Rm‐LR), F1 = 0.607 (by Rm‐LR), MCC = 0.473 (by Rm‐LR) and ACC = 0.773 (by Rm‐LR) (Table S1, Supporting Information). We next compared the performance of these methods on 19 other RBPs from certain variants of the CLIP‐Seq protocol (such as HITS‐CLIP and PAR‐CLIP),^[^ ^33^ ^]^ and ensured that these 19 RBPs have no significant sequence homology (<80%) with the 22 RBPs mentioned for fine‐tuning. Figure S1 (Supporting Information) shows 3UTRBERT also outperformed other methods on these RBPs (AUROC = 0.865 vs 0.850, AUPRC = 0.685 vs 0.646, F1 = 0.752 vs 0.607, MCC = 0.562 vs 0.547, and ACC = 0.871 vs 0.864, more details are in Table S2, Supporting Information), which demonstrated the stability and applicability of the proposed method. We note that, although BERT‐RBP was specifically designed to study RBP‐RNA binding, it did not achieve the best performance, perhaps due to lack of pre‐training steps.^[^ ^31^ ^]^ This was also reflected in DNABERT,^[^ ^22^ ^]^ since both methods only have different thresholds for *k*‐mer (BERT‐RBP with 3‐mer, DNABERT with 6‐mer). And for those NLP models pre‐trained from non‐coding RNAs (including RNABERT, RNA‐FM and RNA‐MSM), they also failed to perform well since the significant differences between mRNAs and ncRNAs in function, structure and data characteristics^[^ ^25^, ^26^, ^29^ ^]^ (Figure S2, Supporting Information). Unlike the key role of ncRNA structure in functional realization, the mRNA domain that controls the critical post‐transcriptional gene regulation process mainly depends on the translation regulatory elements and open reading frames (ORFs) in the sequence,^[^ ^2^ ^]^ resulting in better predictive performance achieved by 3UTRBERT. Other deep‐learning or graph‐based approaches also did not perform as well due to likely architectural complexity and insufficient representational capabilities. Interestingly, Rm‐LR achieved the second best performance on different sequencing protocols, especially on 19 RBPs containing 5'UTR and CDs regions. This is because the BERT model was trained on pre‐mRNA sequences, but it performed poorly on 3'UTR‐specific regulatory tasks since the sequence context contains much information about non‐coding regions.^[^ ^30^ ^]^ Overall, these results demonstrated that the representation learning approach implemented in 3UTRBERT was an effective approach in finding RBP binding sites located in the 3'UTRs.

Beyond that, a “general model” was developed to handle any RNA‐binding proteins (RBPs) with little or no target information but with known binding preferences. We first pooled known targets from different RBPs under eCLIP sequencing protocol, and the sequence similarity was filtered by CD‐HIT with the threshold of 0.6 to balance the tradeoff between non‐redundancy and the quantity of training data. For the sake of fairness, we assessed the performance evaluation on the same independent test used in RBP‐specific strategy, and ensured that the data of the test sets were fully excluded from the training data. As shown in Figure 2b, 3UTRBERT‐generic model achieved the best performance compared to other methods (AUROC = 0.768, AUPRC = 0.637, F1 = 0.601, MCC = 0.377, and ACC = 0.730), while the second‐best results were AUROC = 0.755 (by Rm‐LR), AUPRC = 0.615 (by Rm‐LR), F1 = 0.588 (by Rm‐LR), MCC = 0.351 (by Rm‐LR) and ACC = 0.684 (by Rm‐LR) (Table S3, Supporting Information). Unsurprisingly, the overall prediction performance slightly decreased, but some individual RBPs benefited (such as G3BP1_HepG2, LIN28B_K562, UCHL5_K562 and ZNF622_K562) since the expansion of training data brought more target information, which gave RBPs with similar binding patterns greater learning and discrimination capabilities. This was also reflected in 31 CLIP experiments on 19 RBPs (Figures S3 and Table S4, Supporting Information). Moreover, we also conducted another comparison to show the generalizability of 3UTRBERT on 5' untranslated regions (5'UTRs) and coding sequence regions (CDs) of mRNAs. RBPs with significant eCLIP peaks in both regions were separately selected to build the benchmarks following the same data processing in the Experimental Section. As shown in the left half of Figure 2c, Rm‐LR provided the best prediction on coding regions (AUROC = 0.821, AUPRC = 0.815), outperforming 3UTRBERT by AUROC = 0.810 and AUPRC = 0.800 (Table S5, Supporting Information). Benefiting from the pre‐training process of pre‐mRNA exons, RM‐LR was more skillful in handling binding preference prediction of protein‐coding genes. However, in the 5'UTR regions, 3UTRBERT achieved the better performance since 3'UTR and 5'UTR are both translation regulatory elements (Table S6, Supporting Information), perhaps shared similar grammar and semantics.

**Figure 3:**
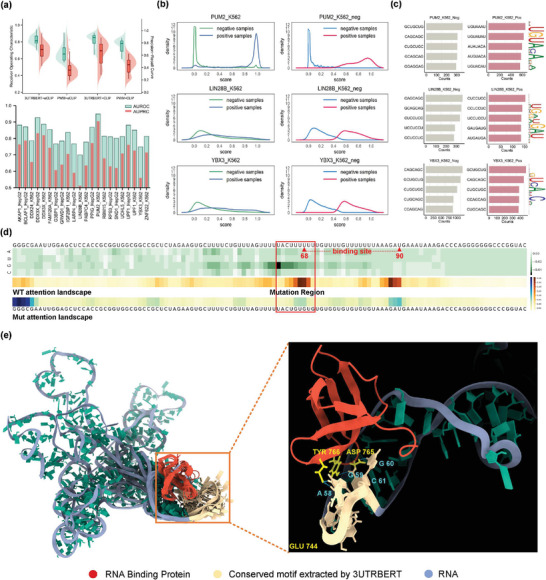
a) Performance comparison between 3UTRBERT semantic embedding and PWM‐based representation on different CLIP datasets, with AUROCs in green and AUPRCs in red (the top half), and the corresponding AUROC and AUPRC scores are shown in the barplot below. b) Distribution of prediction scores for positive and negative samples on PUM2_K562, LIN28B_K562, and YBX3_K562 (left half); the right half shows the density of false positive hits in negative samples. c) The top five 7‐mer counts in the positive and negative samples, along with the significant binding motif extracted by 3UTRBERT. d) 3UTRBERT can assess the impact of functional variants with mutation map. The attentional contribution at the RBP targets decreases with the occurrence of mRNA sequence mutation. e) Predicted complex structure between SND1 and a target RNA sequence (UUCCAGG). Multiple hydrogen bonds are observed: Glu744‐A58, Asp765‐G60, and Tyr766‐G59.

Figure 2d shows the landscape of attention scores for RBM15 and SND1. For positive samples, the nucleotides adjacent to the cross‐linking sites tended to have the highest attentions scores in the 100‐nucleotide window. However, the attention score of negative samples did not have such enrichment. We next investigated whether these continuous high attention regions were enriched with previously known RBP binding motifs. In agreement with the RNA binding sequence motifs for RBM15 and SND1 under crosslinking experiments,^[^ ^34^ ^]^ 3UTRBERT found the similar motif patterns from the target transcripts (Figure S4, Supporting Information), where UAC‐rich was the significantly enriched motif in SND1 binding to m6A‐modified exons.^[^ ^35^ ^]^ Then we used different cutoffs to select regions of high attention scores and extracted enriched sequence motifs for all RBPs. These sequence motifs were subsequently converted to positional weight matrices (PWMs) and compared with known RBP binding motifs collected in the ATtRACT database.^[^ ^36^ ^]^ By using the TOMTOM search tool^[^ ^37^ ^]^ with an FDR *q*‐value <0.01, 133 documented motifs were discovered and matched to the candidate motifs discovered by attention scores. We further systematically visualized the hierarchical clustering of all validated motifs (Figure 2e), RBPs involved in similar RNA regulatory pathways were generally grouped together within the canonical RNA binding domains (RBD).^[^ ^38^ ^]^ For example, HNRNPK clearly prefers to cytosine (C)‐rich motifs within 3'UTR of K‐Homology (KH) domain to stabilize target mRNAs, the dysregulation of which is implicated in cancer progression.^[^ ^39^ ^]^ And ELAVL1‐mRNA enrichment are mostly dependent on 3'UTR association, the highly conserved RBDs belonging to the RNA‐recognition motif (RRM).^[^ ^40^ ^]^ Compared with the static and pre‐defined PWM search methods, 3UTRBERT based on self‐attention mechanism is naturally suitable for locating regulatory regions in transcriptome by dynamically learning the complex dependencies between nucleotides. As shown in the top half of **Figure** **3**a, under the RandomForest classifier prediction (n_estimators = 100, max_depth = 3), the semantic embedding representation of 3UTRBERT (AUROC‐eCLIP = 0.826, AUPRC‐eCLIP = 0.698; AUROC‐CLIP = 0.842, AUPRC‐CLIP = 0.750) outperformed PWM‐based sequence features (AUROC‐eCLIP = 0.669, AUPRC‐eCLIP = 0.482; AUROC‐CLIP = 0.633, AUPRC‐CLIP = 0.428) at 41 CLIP‐seq RBPs. The detailed distribution of RBP prediction scores (bottom half of Figure 3a) demonstrated that case with high AUROC and AUPRC scores (PUM2_K562) showed clear differences between positive and negative samples (left half of Figure 3b), whereas for RBP with lower scores (LIN28B_K562 and YBX3_K562), there was little discrimination between positives and negatives. Variation in classification performance could result from the false positive hits, where many negative samples were misclassified as genuine binding sites resulting in lower performance (right half of Figure 3b). To investigate this, we ranked the inherent 7‐mer sequence patterns in positive and negative samples, where the top five were selected and checked against the significance motifs extracted by 3UTRBERT (Figure S5, Supporting Information). We found that in RBPs with higher false positive rates, there were a large number of redundant sequence distributions between positive and negative samples (e.g., GCUGCUG and CUGCUGC in YBX3; CUCCUCC and UCCUCCU in LIN28B), whereas the differences were quite clear in PUM2 (Figure 3c). Compared with the linear motif representation method of PWM‐based search, 3UTRBERT provided more candidate motifs by considering the dependencies of the global sequence context (e.g., both UGU‐rich and ACA‐rich variants in PUM2, and AUG‐rich variants in LIN28B), and the increase in their matching degree with the reference motifs would reduce the occurrence of false positive hits. Similarly, the quality of each CLIP data is another important factor, the reduction of sequence bias will also lead to better discrimination of binding sites. Additionally, genetic variation in the 3'UTR potentially changed the regulatory elements that affect the protein interactions, therefore 3UTRBERT was applied to identify functional sequence variants on RBP binding. For instance, ELAVL1 (also called HUR) bound to the 3'UTR on ERBB‐2 oncogene mRNA and thus modulated the expression of ERBB‐2 in breast cancer cells.^[^ ^41^ ^]^ Following the experimental observation, we quantified the effect of the difference in probability scores between wild‐type and mutation on each base of interest, and the specific model was fine‐tuned on the CLIP‐seq dataset of ELAVL1 measured from the Hela cells.^[^ ^42^ ^]^ As shown in Figure 3d (heatmap in green), 3UTRBERT provided a clear identification of functional variant regions and mutational trends (Uracil to Guanine). More importantly, the attention landscape of wild‐type sequence (heatmap in orange) indicated that our model focused attention precisely on the RNA–protein interaction regions. And the degree of attention dropped with the occurrence of mutations inside mRNA (heatmap in yellow).

We next carried out RNA–protein docking and molecular simulation experiments to test the physical interactions between RBPs and RNA motifs identified by 3UTRBERT. Due to computational complexity, we selected RBM15 and SND1 for this study since these are well‐known RBPs implicated in critical cellular processes such as m6A modifications and tumor angiogenesis.^[^ ^35^, ^43^ ^]^ We used the experimentally determined crystal structure for RBM15 (PDB ID Q96T37) and SND1 (PDB ID Q7KZF4).^[^ ^44^ ^]^ For each of these RBPs, we took the sequence motif of the highest attention score, GGCCCG for RBM15 and UUCCAGG for SND1 (Figure S6a, Supporting Information). We investigated several 3D RNA structure prediction tools and used DeepFoldRNA;^[^ ^45^ ^]^ we confirmed that the structure predictions converged and returned converged final structures. We next used the ClusPro web server to perform RNA–protein docking to generate high‐resolution 3D RBP‐RNA complexes.^[^ ^46^ ^]^ ClusPro uses PIPER to generate docking samples, which implements a Fast Fourier Transform (FFT) algorithm with structure‐based pair‐wise interaction terms. Specifically, it uses shape complementarity, electrostatic, and desolvation terms to produce the near‐native binding complexes. ClusPro further clustered the 1000 most energetically favorable samples by root‐mean‐square deviation (RMSD), and then performed structure refinement on the cluster centers using Charmm energy minimization, ultimately returning top ten binding complexes.^[^ ^47^ ^]^ After acquiring these samples, we filter for those involving interactions with the RNA motifs identified by our method. For RBM15‐RNA complexes, the median PIPER energy score for top ten docked complex was –1792.6, –1839.4 for top five complexes; for SND1‐RNA complexes, the median PIPER energy score for top ten docked complex was –1269.1, –1310.1 for top five complexes. We note that these numbers were calculated from scoring functions, which were proportional to free energies but did not have actual units. After docking experiments, we subsequently conducted molecular dynamic (MD) simulation on docked RBP‐RNA complexes after filtering to further refine the RBP‐RNA complexes and evaluate interactions between RBP residues and RNA nucleotides. Figure 3e and Figure S6b (Supporting Information) display the average structures for the final 50 ns of simulation (more details in Note S2, Supporting Information). We observed formation of strong phosphate contacts, accompanied by supplementary base contacts, further supporting the reliability of the predicted RNA‐protein interactions. In the case of RBM15, the predicted RNA motif was located at a loop forming a pseudoknot; the side chain residues of the first three beta sheets of RBM15 flipped the nucleotides on the motif outward, forming two notable hydrogen interactions: (ASP404 ‐ G10) and (LYS450 ‐ G11), and the phosphate interactions were also observed for (THR375, G11) and (LYS420, G10, G11). For SND1, the bound RNA motif was situated on a hairpin loop; the side chains on the second to fourth beta sheets of SND1 were inserted into the hairpin, forming multiple hydrogen bond interactions with the bases, specifically (GLU744 ‐ A58), (ASP765 ‐ G60), and (TYR766 ‐ G59). Interestingly, the predicted electrostatic interactions with nucleic acids GGA (58‐60) have been previously reported as a binding site of SND1 for regulating mRNA functions.^[^ ^48^ ^]^ Overall, our docking and MD experiments, despite with only on two RBPs, showed that the RNA motifs discovered by 3UTRBERT were biologically meaningful and could generate insights on interactions between RBP and target RNA sequences.

### 2.3 3UTRBERT Enables Identification for Dynamic Cellular Epitranscriptomic Modifications

Inside cells, RNA transcripts undergo extensive covalent modifications. Among the estimated dozens of types of modifications, m6A (N6‐methyladenosine modification) is the most prevalent and most studied. It is reported that over 70% of the mammalian mRNA transcripts inside cells undergo m6A modifications and m6A is implicated in many important developmental processes and human diseases.^[^ ^4^, ^49^ ^]^ Many of these modification sites are evolutionarily conserved, although there are debates on the strength of these evolutionary constrains.^[^ ^50^ ^]^ We hypothesize that evolutionary conservation and sequence contexts of these m6A sites would allow 3UTRBERT pick them out from background sequences. Toward this goal, we fine‐tuned the 3UTRBERT model to predict potential m6A modification sites. As described in the Experimental Section, the ground truth m6A sites were downloaded from m6A‐Atlas database.^[^ ^51^ ^]^

**Figure 4:**
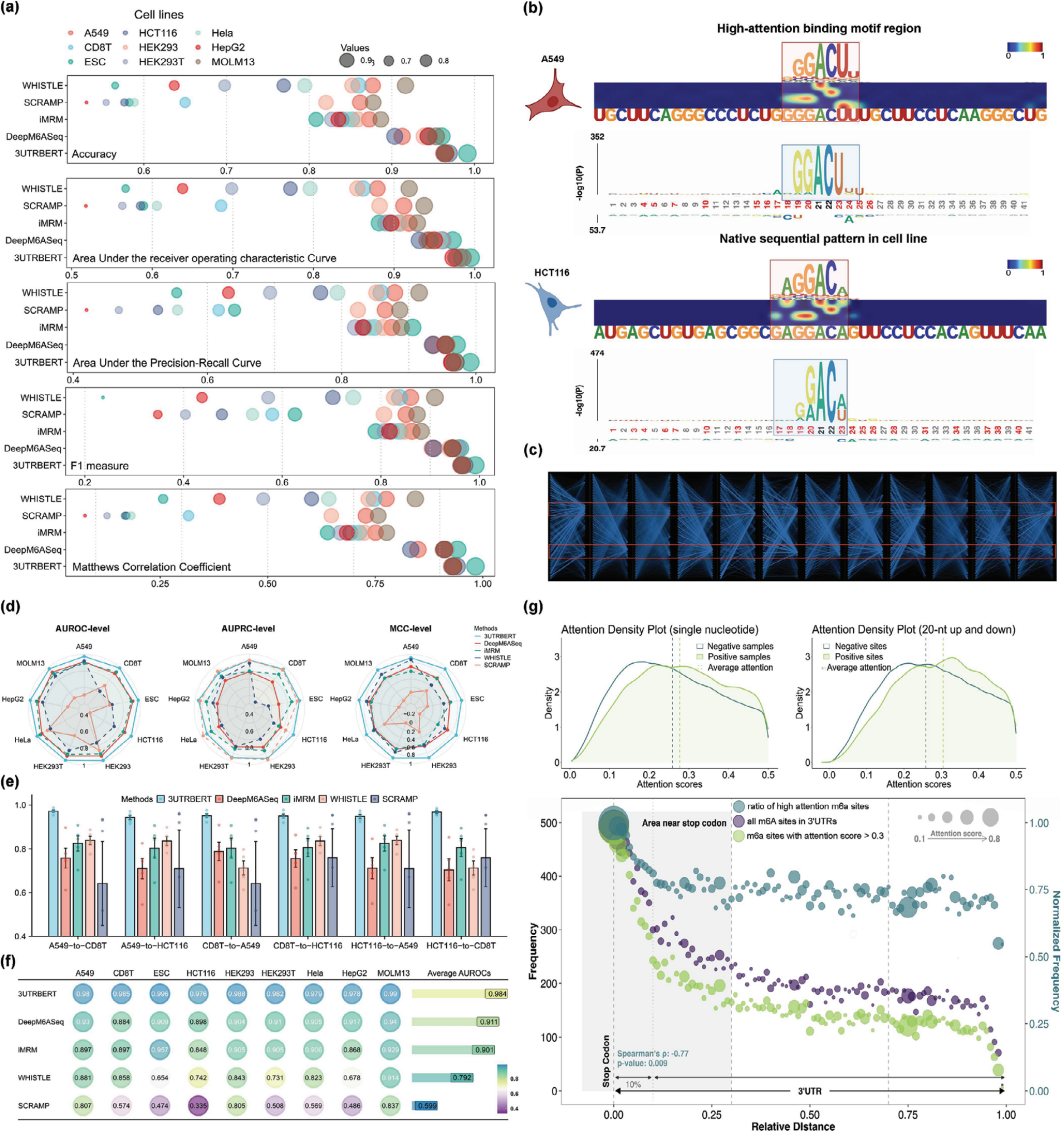
a) Bubble plots comparing prediction performance between 3UTRBERT and other methods. Each panel corresponds to a statistical metric: Accuracy, AUROC, AUPRC, F1 measure, and Matthew's correlation coefficients. b) Two examples of predicted m6A sites with high attentions scores and consistent with well‐defined RRACH m6A motif. c) A bird's‐eye view of global attention scores throughout entire architecture, showing 3UTRBERT correctly self‐focused on two converged regions (red boxed) corresponding to known m6A modification sites. d) Performance comparison between 3UTRBERT and other baselines using ninefold cross‐validation under model‐generic strategy. e) 3UTRBERT has enough power to perform dynamic modification prediction under cross‐cell line conditions, where the A549, CD8T and HCT116 samples have significant differences in motif signals. f) Impact of gene‐specific regulatory relationships on generalization performance (measured by AUROCs). g) (top): distribution of attention scores of experimentally determined m6A sites (positive) and negative control sites, at single nucleotide level and within 40 nucleotides window. (bottom): Distribution plots showing the predicted m6A sites are enriched near stop codons (see text).

We benchmarked 3UTRBERT with several other m6A sites predictors, including machine learning (ML) based methods SRAMP,^[^ ^52^ ^]^ WHISTLE,^[^ ^53^ ^]^ iMRM^[^ ^11^ ^]^ and a deep learning (DL) based method DeepM6ASeq.^[^ ^54^ ^]^ The results are illustrated in **Figure** **4**a and Table S7 (Supporting Information) (including evaluation metrics Accuracy, Area Under the Receiver Operating Characteristic Curve, Area Under the Precision‐Recall Curve, F1‐measure, and Matthews Correlation Coefficient), showing that 3UTRBERT mostly outperformed other methods in five different metrics across nine different cell lines. SRAMP and WHISTLE used hand‐crafted features and generally didn't perform as well as other methods, whereas iMRM depended heavily on cross‐species conservation. DeepM6ASeq was based on a hybrid deep learning network architecture and generally achieved the second‐best performance, just below 3UTRBERT. Figure 4b shows two examples from two cell lines (A549 and HCT116) as depicted by visualization tool kpLogo,^[^ ^55^ ^]^ showing high attention scores associated with experimentally determined m6A site from m6A‐Atlas database. Additional examples can be found in Figure S7 (Supporting Information). Interestingly, we found that our learned consensus motifs (highlighted with red‐colored boxes) also closely matched the consensus m6A motif RRACH (blue‐color window) (where A = m6A, R = purine, and H = A, C, or U).^[^ ^56^ ^]^ The Transformer architecture in 3UTRBERT allowed us to visualize the transition and convergence of attention scores in a sequence. As an example, Figure 4c provides a bird‐eye view across 12 attention heads on an RNA sequence. The sequence was represented as a series of 39 3‐mer tokens, in addition to [CLS] and [SEP]. 3UTRBERT correctly identified two important regions (red boxes with self‐attention converged), which were known m6A sites. By adjusting thresholds, we were able to uncover the hidden semantic relationship embedded in the crucial token “ACU,” which aggregated the contribution attentions from various short regions (Figure S8a, Supporting Information). Moreover, to protect against super‐high performance from over‐training, we conducted another generalizability comparison between 3UTRBERT and other methods. The ninefold cross‐validation was employed where each of the nine cell lines was randomly selected as the test set to evaluate model performance, and the remaining eight cell lines were merged as training data for modeling (de‐redundancy was performed on the whole data using a CD‐HIT threshold of 0.6, and the test samples were fully excluded from the training data). We used the AUROC, AUPRC, and MCC to evaluate their performance on imbalanced datasets, as shown in Figure 4d, the average performance of 3UTRBERT (AUROC = 0.983 ± 0.004, AUPRC = 0.946 ± 0.019, and MCC = 0.929 ± 0.013) was still superior to other baselines, while the second‐best results were AUROC = 0.913 ± 0.016 (by DeepM6ASeq), AUPRC = 0.938 ± 0.042 (by SCRAMP), and MCC = 0.672 ± 0.03 (by DeepM6ASeq). Meanwhile, from Figure 4b and Figure S7 (Supporting Information), we found that the potential binding motifs of the nine cell lines belonged to three categories (ACU‐rich, ACA‐rich, and GAC‐rich). We further followed those three significant binding motifs to select A549, CD8T, and HCT116 cell lines (each corresponding to only one binding mode) for dynamic prediction under cross‐cell line conditions, that is, a model trained on one cell line to predict a different cell line. As shown in Figure 4e and Table S8 (Supporting Information), the robustness of 3UTRBERT in different scenarios was still superior to comparison methods under five types of measurements, yielding up to 14.6% higher overall performance compared to the second best method (by iMRM). Considering that the potential regulatory relationship between transcripts of different genes in each cell line may influence the prediction effect, based on the CD‐HIT‐EST filtering samples, we also divided five non‐redundant datasets according to gene name and then used the fivefold cross‐validation strategy to compare the performance. As shown in Figure 4f and Figure S8b (Supporting Information), 3UTRBERT also achieved the highest average AUROC and AUPRC scores across nine cell lines. Benefiting from the multi‐head self‐attention mechanism, 3UTRBERT has the ability to learn the dynamic contextual semantics within nucleotide sequences, which potentially captures the “biolinguistic grammar” of functional regulations, leading to applicability for epitranscriptomic modifications in different cellular scenarios.

We next investigated whether the globally known m6A modification sites had higher attention scores than the background. Figure 4g (top) plots the distribution of the attention scores of the experimentally determined m6A sites and negative control positions (please refer to the Experimental Section for the choice of negative set), which shows that the m6A sites have overall higher attention scores. We investigated how the attention scores were spatially distributed among 3'UTRs, by scaling 3'UTR sequences into the same length and calculated the frequency of high attention scores along the sequence; for example, relative distance of an m6A site at position 10 on a 50 nucleotides‐long 3'UTR was calibrated as 0.2 (10/50). The bottom of Figure 4g shows that m6A sites were enriched in the region closer to stop codons, and the purple bubble curve shows that the number of m6A sites decreased with growing distance from the stop codon. We next asked whether the m6A sites closer to stop codons also have higher attention scores, i.e., being stronger modified sites. We applied a threshold of 0.3, which is the average attention score over aggregated 3'UTRs, and retained only those m6A sites with attention scores higher than 0.3 (green circles). It is clear that higher attention m6A sites are also accumulated near the stop codons, which has been previously shown.^[^ ^56^, ^57^ ^]^ We next calculated the fraction of high‐attention m6A sites over total number of m6A sites in sliding windows (dark blue circles), which shows that indeed the high attention scores tend to be enriched closer to stop codons, i.e., negatively correlated with the distance to stop codons (Pearson correlation = –77% and *p*‐value < 0.009). These observations demonstrated that indeed the attention based 3UTRBERT can effectively predict and recover experimentally determined m6A sites.

### 2.4 Contextual Embedding of 3UTRBERT Helps to Predict mRNA Subcellular Localization

**Figure 5:**
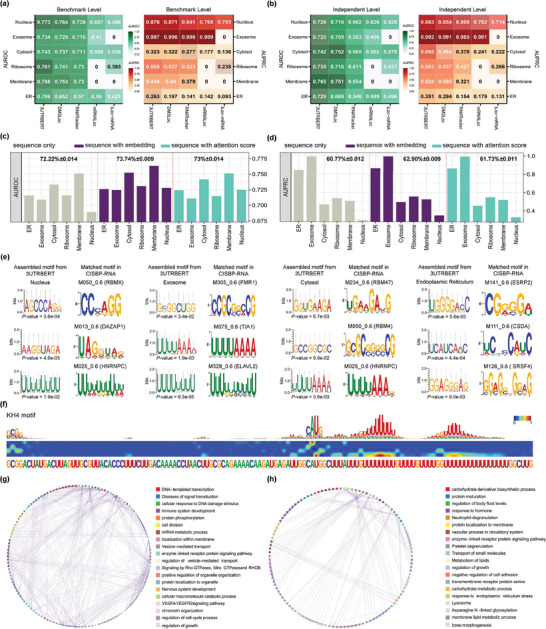
a) Comparison of prediction performance between 3UTRBERT and other methods on mRNA subcellular localizations. Localization data from DM3Loc was used. b) Prediction performance on a newer independent set of localization data from RNALocate 2.0. c,d) Bar diagrams separately plot the AUROC and AUPRC performance on identical model architecture of different feature schemes for multi‐label mRNA subcellular localization prediction. e) Visualization of the top three localization‐specific sequence motifs. The assembled sequence motifs by 3UTRBERT are then compared against known targeting signals from CISBP‐RNA database. f) 3UTRBERT is naturally suitable for locating functional regulatory regions on the zipcode sequence, which controls the mRNA transport by interacting with ZBP1 and HuD protein. g,h) The Gene Ontology Network to capture the relationships between multiple enriched terms from Endoplasmic Reticulum (left) and a set consisting of nucleus, exosome, cytosol, and membrane (right), respectively.

The 3'UTR of mRNA transcripts contain sequence elements that can help direct transportation of mRNA transcripts to specific subcellular compartments.^[^ ^58^ ^]^ These experimentally determined localization data have been cataloged in databases such as RNALocate and RNALocate v2.0^[^ ^59^, ^60^ ^]^ (see Experimental Section). Several computational algorithms have been published, aiming to predict mRNA localization from RNA sequences or structure signatures. These methods include DM3Loc,^[^ ^12^ ^]^ RNATracker,^[^ ^61^ ^]^ mRNALoc,^[^ ^62^ ^]^ and iLoc‐RNA.^[^ ^63^ ^]^ Briefly, iLoc‐mRNA and mRNALoc use a Support Vector Machine (SVM) with hand‐craft features. RNATracker integrates sequence and secondary structure information using a neural network framework. DM3Loc uses multi‐head self‐attention mechanism in predictions; notably it also predicts multi‐label subcellular localizations, i.e., allowing an mRNA transcript present in multiple subcellular compartments.^[^ ^12^ ^]^ Despite promising results, these methods still have limitations, as it is difficult to model long‐range dependencies among nucleotide positions by the widely used sparse positional encoding scheme. The major innovation of 3UTRBERT versus the other methods is that it creates an informative coding scheme by generating context‐sensitive embedding with self‐learning. Adopting a framework similar to DM3Loc, we trained 3UTRBERT for the subtask of predicting RNA localization and benchmarked against four other methods. The proposed model was compared with the DM3Loc and RNATracker from a deep‐learning method perspective, where the same benchmark sets were used to train and test those three methods (please see Experimental Section for details); and we also compared 3UTRBERT with mRNALoc and iLoc‐mRNA from a tool perspective (due to non‐open source codes and limitations on the types of localization compartments), where the five folds of independent tests from the fivefold cross‐validation benchmark data were employed to evaluate those tools. **Figure** **5**a summarizes the performance metrics of AUROC (Area Under the Receiver Operating Characteristic) and AUPRC (Area Under the Precision‐Recall Curve). Only the compartments predicted by all five methods are listed; the panel on Figure 5a shows that 3UTRBERT had the best performance on both AUROC and AUPRC, with the DM3Loc coming in second in performances.

To further evaluate the robustness of 3UTRBERT in predicting mRNA subcellular localizations, we conducted another benchmarking experiment on an independent dataset from RNALocate v2.0 database,^[^ ^60^ ^]^ containing a collection of mRNA sequences that were excluded from all previous datasets. Employing the models described above, we directly fed the test data to the model to make predictions (as shown in Figure 5b). 3UTRBERT also achieved the best performance, achieving AUROC of 0.737, and mean average area under Precision‐Recall (AUPRC) of 0.66, over six compartments. The second‐best results were AUROC = 0.722 and AUPRC = 0.613 (by DM3Loc). We then calculated the average prediction matrices using three different coding schemes (sequence with embedding, sequence with attention score, and sequence only) of six subcellular localizations for comparison (Figure 5c,d). The embedding and attention‐based approaches achieved better performance (AUROC = 73.74% ± 0.009, 73% ± 0.014; AUPRC = 62.9% ± 0.009, 61.73% ± 0.011) than sequential information alone (AUROC = 72.22% ± 0.014; AUPRC = 60.77% ± 0.012), suggesting that the contextual feature from deep representation learning helped to enhance the original model. Meanwhile, to explore whether the modeling of RNA trafficking revealed the inherent functional elements that determined computational localization predictions, we employed 3UTRBERT to extract the potential targeting signals in each of the six compartments. As shown in Figure 5e and Figure S9 (Supporting Information), the “Poly‐U rich,” “AU‐rich” elements (AREs), and “GC‐rich” identified by 3UTRBERT were particularly significant motifs that derived subcellular localization across all compartments. Among them, AREs were normally located in the 3'UTR of mRNAs, which guided the precise localization of mRNA in cells by interacting with RNA‐binding proteins.^[^ ^3^ ^]^ For each specific localization, the expressive “CCA‐rich” in the nucleus was matched to the binding motif of RBMX from CISBP‐RNA database, which could regulate alternative splicing;^[^ ^64^, ^65^ ^]^ “GC‐rich” found in the top targeting signals of exosomes was a binding motif identified by FMR1 that served an important function in endosome cargo loading.^[^ ^66^ ^]^ Also, the “GA‐rich” in cytosol, “GC‐rich” in ribosome and “GGG‐rich” in endoplasmic reticulum were completely consistent with the findings mentioned in iLoc‐mRNA.^[^ ^63^ ^]^ While the targeting motif such as “CGA‐rich” was not extensively documented in the database, it probably represented a common sequential pattern for guiding RNA into the membrane. To further demonstrate the sensitivity of 3UTRBERT to functional signaling regions, we collected a zipcode sequence localized at 3'UTR, which contains the RBP binding region from ACTB gene to identify the targeting motifs associated with ZBP1 and HuD.^[^ ^67^ ^]^ As shown in Figure 5f, the high‐attribution positions naturally located by 3UTRBERT corresponded to the functional regulatory regions of the sequence. The KH4 recognition motif 5′‐CGGAC‐3′ at the beginning of the zipcode and “Poly‐U rich” features across the entire ACTB sequence were confirmed as the potential targeting signals bound to the both protein, highlighting the interpretability of 3UTRBERT in 3'UTR‐specific cellular localization.

In addition, we found that the semantic representation significantly facilitated the performance of mRNA localization to endoplasmic reticulum (ER), as measured by AUROC, which respectively improved 5.4% on benchmark, and 4% on independent test set. For more intuitive explanation, we performed non‐redundant enrichment clustering with Metascape,^[^ ^68^ ^]^ simultaneously eliminating the confounding data interpretation issues that arose from the reporting of multiple ontologies. The comparison experiments were separately conducted on the mRNA genes from ER (Figure 5g) and on the set consisting of nucleus, exosome, cytosol, and membrane (Figure 5h). Rendered by the GO Network diagrams, each node represented an enriched term and colored by its functional description, where terms with Kappa similarities above 0.3 were bridged by edges. Obviously, the network plot of ER revealed more relationships between the terms within benchmark than that a group of four sub‐compartments, and displayed the identical phenomenon on the independent test set (Figure S10, Supporting Information). This also explained the superior localization effect of 3UTRBERT‐embedding in ER compared to the remaining compartments, on the one hand, more functional information promoted 3UTRBERT to gain deep understanding and rich representation within sequential mRNAs, and the sufficient learning of semantic contextualization during pre‐training phase ensured effective generalizability for the correlated modulation task.

### 2.5 3UTRBERT can Derive Semantic Information from Biological Sequences

**Figure 6:**
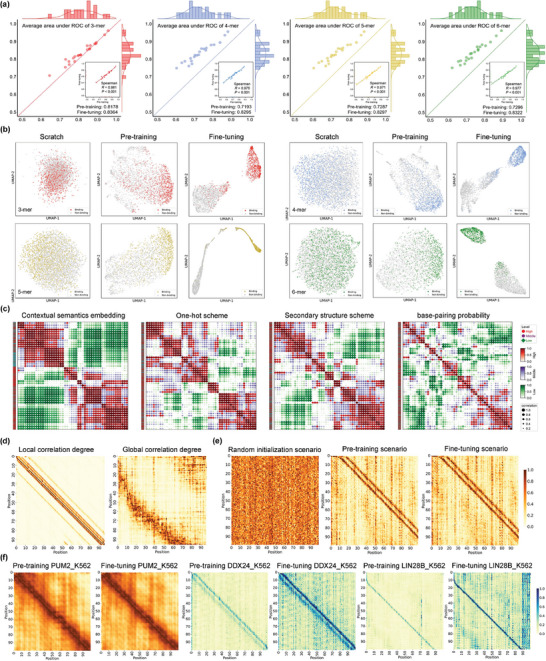
a) Scatter plots of AUROC scores along with the Spearman's rank correlation coefficient after pre‐training (X‐axis) and after fine‐tuning (Y‐axis) for 22 eCLIP‐RBP datasets. Different *k*‐mer sizes are shown separately, and fine‐tuning process has generally improved the prediction performance. b) UMAP projection of the embedding vectors generated by 3UTRBERT for RBP binding sites (positive) and negative controls; different stages with *k*‐mer sizes are shown. c) Clustering of positive RNA sequences bound by RBM15 as encoded by four different coding schemes: 3UTRBERT, one‐hot scheme, secondary structure, and a graph‐based embedding. d) Attention maps illustrating that 3UTRBERT can extract both local and global contextual semantics. e,f) Illustration of how information is extracted at scratch, pre‐training and fine‐tuning stage, the strength of RBP target signal distribution will affect the learning efficiency of 3UTRBERT.

The 3'UTRs of eukaryotic mRNA transcripts contain important sequence motifs that are under evolutionary and functional constraints, which may have evolved in a manner similar to the evolution of syntax and grammars of human languages.^[^ ^19^ ^]^ This allows us to use advanced nature language models, especially attention mechanisms, to infer and visualize the contextual information between these motifs. Such semantic information can be extracted by self‐attention mechanism within Transformer model to weigh and capture relationships between different tokens in the mRNA sequences. The self‐attention mechanism operates by assigning attention scores to each token based on its dependencies on other tokens, thus helping 3UTRBERT capture the intricate patterns between different elements within the 3'UTRs, even if they are separated by several tokens. To search for the most optimal *k*‐mer size to balance the computational time and the representation capability, we first tested four types of *k*‐mer (*k* = 3, 4, 5, 6) with 1‐stride settings. **Figure** **6**a displays the evaluation results before and after training at different *k*‐mer size for the 22 eCLIP‐RBP results, both x‐axis and y‐axis are equalized to the same scale to prevent distortion of the resulting distribution. For example, the top left panel compares the prediction performance, as measured as AUROC, after the pre‐training stage (shown on the horizontal axis) and after the fine‐tuning stage (shown on the vertical axis). It is clear that, for all the *k*‐mer size tested, the task‐specific fine‐tuning always improved the prediction performance compared to the task‐agnostic pre‐training stage. We observed that *k*‐mer = 3 had the best prediction performance with averaged AUROC = 0.8178 after pre‐training and AUROC = 0.8364 after fine‐tuning, comed with the best Spearman's rank correlation coefficient *R* = 0.981 (panel on the lower right). We also explored fine‐tuning directly on the scratch model, i.e., skipping the pre‐training phase and directly training the model on a particular task with randomly initialized parameters. As expected, the results and stability of all *k*‐mers (0.7208 ± 0.005, 0.6866 ± 0.007, 0.6797 ± 0.009, and 0.6662 ± 0.012) dropped significantly (Figure S11, Supporting Information). This shows that the pre‐training stage learned from a large corpus of unannotated RNA sequences and captured valuable contextual knowledge that can be leveraged for various downstream tasks.

We next tried to project and visualize the feature space of the positive and negative (bound and unbound) RNA sequences as decoded by 3UTRBERT (the hidden states of the last layer). Taking the best performance PUM2_K562 as an example (please see Results Section), Figure 6b employs UMAP (Uniform Manifold Approximation and Projection) to visualize the input RNA sequences as 2D projections,^[^ ^69^ ^]^ using different *k*‐mer sizes. Each data point represents a sample input RNA sequence (100 nucleotides long), predicted positive (bound) sequences are painted in color while negative (unbound) sequences are in grey. These figures clearly show how the embeddings generated by 3UTRBERT effectively separate positive and negative samples in three stages: parameter random initialization (scratch) stage, pre‐training stage, and fine‐tuning stage. However, we find that there is still a mixture of binding and non‐binding points on some RBPs (Figure S12a, Supporting Information). This resulted from the model confusion caused by potential sequence bias within positive and negative samples. Specifically, we visualize the changes in the significant sequential enrichment extracted by 3UTRBERT from the positive and negative samples during the pre‐training and fine‐tuning stages (Figure S12b, Supporting Information). Compared with the known targeting signals from mCrossBase,^[^ ^34^ ^]^ we find that 3UTRBERT effectively localizes to the UGUA‐rich motif of PUM2 in both stages, along with clear non‐biased for positive and negative samples. As for the RBM15 protein, 3UTRBERT also captures the known UUG‐rich motif within positive sequences, but similar sequence patterns are also found in negative samples (UGC‐rich and CCA‐rich), which increases the difficulty of model classification. Then we investigated whether the language model provided a better clustering effect between input RNA sequence samples than other encoding schemes. Figure 6c compares the 3UTRBERT token embedding scheme with one‐hot scheme, secondary structure, and a graph‐based embedding scheme on RNA sequences bound by RBM15 as input. For the sake of fairness, we use RandomForest (n_estimators = 100, max_depth = 3) to evaluate the contribution of each feature to the model performance and consistently select the top‐50 highly expressed features. In one‐hot scheme, each nucleotide is represented as a binary vector according to base type; in secondary structure scheme, each nucleotide is represented as one of the five structure categories (E for external loop, H for hairpin loop, I for internal loop, M for multi‐loop, and P for paired); in graph‐based embedding, each RNA sequence is represented as a graph where nucleotides are nodes and their interactions or spatial proximities are edges. We can see that the contextual semantic embedding by 3UTRBERT gave stronger and cleaner clustering of positive bound sequences. One advantage of attention‐based nature language models such as 3UTRBERT is that they can capture both local and long‐range sequence semantics or interactions. Such relevance can be visualized with attention map, a high‐resolution perspective on RNA sequence analysis. The degree of correlations along the sequences illustrates attention strength between respective positions. By contextualizing RNA sequences both locally and globally (as shown in Figure 6d), 3UTRBERT can help extract correlations or interactions between different elements within 3'UTRs, which are important to understand regulation at post‐transcriptional level. We next portrayed the complete evolutionary processes of hidden semantic relationships (Figure 6e). As we can see, the information is cluttered during the scratch phase, yet by sufficient iterations of the training process, the complex and hierarchical properties are uncovered. For RBPs with similar strong signals (orange panel in Figure 6e,f), 3UTRBERT pays attention to the same regulatory information in both positive and negative samples in two stages (Figure S12b, Supporting Information), so the attention maps share consistency distribution and yield a comparable performance (AUROC = 0.811 vs 0.824 for RBM15 and 0.957 vs 0.961 for PUM2). However, for RBPs with more types of target binding motifs (Figure S12c, Supporting Information), the language model requires a fine‐tuning operation to further enrich the contextual information of each nucleotide on the task‐specific data (AUROC = 0.731 vs 0.798 for DDX24 and 0.646 vs 0.706 for LIN28B), and the self‐attention regions of interest changes more significantly with the increase on the hit rates of known sequence patterns (blue panel in Figure 6f; Figure S12d, Supporting Information).

**Figure 7:**
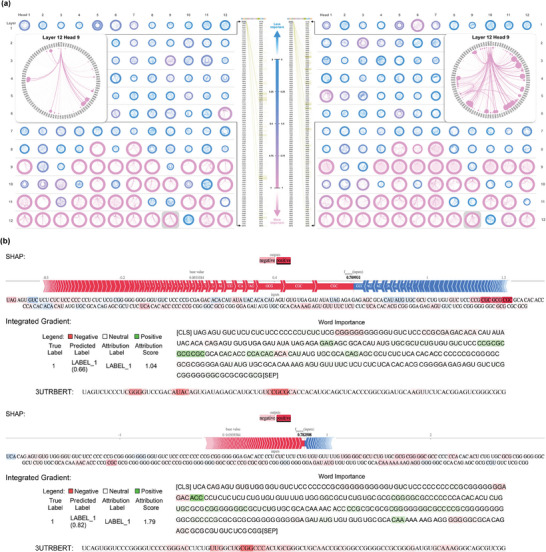
a) Attention Head Overview provides the contribution score in each attention head for the input sequences. The dynamic representation‐based 3UTRBERT shares different contextual information for homologous sequences. b) Textplot visualizes the importance of individual tokens between 3UTRBERT and other reliable explainers (SHAP and Integrated Gradients). A token with a strong positive effect is highlighted (3UTRBERT and SHAP in red color, IG in green color), indicating a positive prediction from the language model. And 3UTRBERT consistently puts high attention at/around the binding of RBP interest.

Unlike static word embedding techniques that generate fixed vector values for the same token, the Transformer model derives different vector representations based on dynamic changes in its surrounding context.^[^ ^70^ ^]^ However, deep learning models, including BERT, are often viewed as “black boxes” since it can be challenging to understand why they make certain predictions.^[^ ^71^ ^]^ Using a novel visualization system Dodrio,^[^ ^72^ ^]^ we linked attention weights with semantic contextual knowledge in order to provide a clear understanding of the learning process of 3UTRBERT. We randomly selected two homologous RNA sequences bound by RBM15 and created **Figure** **7**a to visualize the dynamically generated semantic information. Specifically, these two sequences were parsed by BioPython^[^ ^73^ ^]^ based on a high global alignment score, and then aligned locally using BLAST.^[^ ^74^ ^]^ In Figure 7a, the “Attention Head Overview” displays the relative importance of all attention heads for the input RNA sequences. Each head was comprised of weights incurred from 3‐mer tokens when calculating the next representation of the current token, and then formed a Radial Layout attention map. For the identical position of the attention head, the final hidden vector of the [CLS] token aggregated different semantic relationships, resulting in distinct decisions for downstream classification tasks. More importantly, the distribution of attention heads with high contribution scores increased by the number of layers and peaked at the last layer, which is consistent with the UMAP classification in each layer of the 3UTRBERT model (Figure S13, Supporting Information). In the context of RNA sequences, lower layers are closely associated with local features such as specific nucleotide patterns or motifs, while higher layers gradually integrate the contextual information related to identifying functional sites. We next examine whether the high attention regions predicted by 3UTRBERT reveal the gradual comprehension of input sequence, so a framework named Ferret is employed to evaluate the explanations for plausibility.^[^ ^75^ ^]^ This tool ensembles the state‐of‐the‐art post‐hoc explainers such as SHAP,^[^ ^76^ ^]^ Integrated Gradients (IG),^[^ ^77^ ^]^ and LIME,^[^ ^78^ ^]^ etc., and measures which technique provides better comprehension of the inputs based on several metrics. As shown in Figure S14 (Supporting Information), SHAP (aopc_compr = 0.36 and 0.40) and IG (aopc_compr = 0.38 and 0.44) retrieve the higher number of tokens which contribute to the model decision, and the lower scores of “aopc_suff” indicate that those tokens are strongly associated with driving the predictions. Further, the better “taucorr_loo” scores reflect the importance of the retrieved tokens' actual impact on the model performance. Supported by the above results, we separately employed the optimal explainers including SHAP and IG to visualize the impact of various regions on the RNA sequences (*k*‐mers) on model predictions. The regions with high SHAP values (red color) and IG scores (green color), as visualized in Figure 7b, are correlated with functionally important regions of the RNA, i.e., RBP targeting signals. The conserved “GC‐rich” semantic regions have the same positive effect on model predictions for RBM15 protein. More importantly, 3UTRBERT consistently locates the highest attention at/around the binding of interest (“GC‐rich” and “UG‐rich”). All the results demonstrated that the knowledge‐embedded framework of 3UTRBERT can learn and understand functional semantics of the biological sequences and can be easily extended to other sequence labeling tasks.

## 3 Experimental Section

### 3.1 3'UTR Functional Data Benchmark Datasets

Annotated functional elements in the 3'UTR of human mRNA transcripts were collected and these elements were used as ground truth to evaluate and benchmark the performance of 3UTRBERT. Some of these elements were extracted from previous published studies. All the datasets in this study can be downloaded from https://figshare.com/articles/dataset/3UTRBERT_dataset_availability/26082916/1.

### 3.2 Data Preparation for Pre‐Training

For task‐agnostic pre‐training phase of 3UTRBERT, the human mRNA transcript sequences were downloaded from the GENCODE website (GRCh38.p13, Release 40),^[^ ^79^ ^]^ which consists of 108 573 unique mRNA transcripts. For genes with multiple transcripts, the longest primary transcript was chosen. On average, each of these transcripts contain 3,754 nucleotides (median 3048 nts), 1657 nts for coding region (CDS, median 1215 nts), 276 nts for 5'UTR (median 175 nts), and 1227 nts for 3'UTR (median 631 nts). BioPython was used in sequence manipulation and alignment.^[^ ^73^ ^]^ In the model, sequence window length of 510 nts was used since current BERT model had a maximum sentence length of 512 characters (a special token is added at each end). Each 3'UTR sequence was split into non‐overlapping windows of 510 nts long, the remaining sequence was back‐padded to the same length. As described below, the study was limited to 3'UTR of mRNA sequences to avoid codon constrains in the CDS region, and to reduce increased complexity of the entire mRNA transcripts.

### 3.3 Collection of RBP Binding Sites

Data were collected from 31 published and curated CLIP (crosslinking and immunoprecipitation) experiments corresponding to 19 RBPs (RNA Binding Proteins), which provided nucleotide resolution binding sites of RBPs.^[^ ^16^, ^33^, ^80^ ^]^ These CLIP datasets were processed using the same pipeline, and sequence windows of 101 nucleotides were used in peak calling. Positive datasets (binding sites) were derived from nucleotide positions with the highest read‐counts; binding sites were required to be at least 15‐nucleotides apart to minimize redundancy and prevent neighboring sites. The negative control sets were randomly sampled from the genes without detectable interactions in any of the 31 experiments. In each experiment, a total of 4000 crosslinked sites were randomly drawn for training purpose, and 1000 samples each were drawn for optimization and model validation. To assure separation between training and testing processes, the independent testing set containing 1000 sequences were sampled only from genes unused in model training.^[^ ^33^ ^]^ In addition to these 19 RBPs, a dataset was also collected from the earlier work, which included eCLIP datasets for 22 additional RBPs from two different cell lines, K562 and HepG2.^[^ ^81^ ^]^ The peak intensities on the RBP crosslinking sites were represented as the Irreproducible Discovery Rate (IDR) BED files,^[^ ^82^ ^]^ which were processed by the original authors. All the sequencing data were unified to the length of 100 nucleotides, and the samples with sequence identity over 80% were removed by using CD‐HIT‐EST module.^[^ ^83^ ^]^ The positive‐to‐negative ratio of 1:2 was kept for each eCLIP‐RBP, and the data partitioning outlined above were also complied to maintain an equitable model simulation scenario, i.e., 80% of the samples from each RBP dataset were randomly selected for model training process, with the training to validation ratios set at 4:1. The remaining 20% of the samples were separately reserved for independent testing.

### 3.4 Human m6A Modifications Across Nine Cell Lines

Single nucleotide resolution human m6A modification dataincluding those obtained by different technologies and from nine cell lines were downloaded.^[^ ^51^ ^]^ The final dataset contains a total of 131 703 high‐confidence m6A sites. A negative data set (total 108,573 nucleotides) was also derived by selecting non‐positive adenosine nucleotides on the 3'UTR of the same transcript, using a non‐overlapping window size of 41 nucleotides centered around the selected adenosine nucleotide. CD‐HIT‐EST was run with a cut‐off of 0.8 to remove potential redundancy between the positive and negative datasets, which produced a final 79 021 m6A sites and 849 005 non‐m6A negative set (please refer to Figure S15, Supporting Information). It was noted that such 1:10 positive‐negative ratio was also used in a previous study WHISTLE.^[^ ^53^ ^]^ Following the balancing strategy described therein, random down‐sampling was conducted for each cell‐line by splitting the non‐redundant negative samples into 10 subsets, each with 1:1 positive‐to‐negative ratio. Next, 20% of the mixed positive and negative samples were randomly selected in each subset to construct an independent test set, and ensured that these test data were completely excluded from the remaining training samples. Finally, the model was trained on all subsets of each cell line separately using tenfold cross‐validation, and the testing performances from the ten independent sessions were averaged and reported as final results.

### 3.5 mRNA Subcellular Localization

RNA sub‐cellular localization was regulated by specific cis‐regulatory elements typically found in 5' and 3'UTRs. RNA localization data were downloaded from the RNALocate database,^[^ ^59^ ^]^ as described in a previously published study.^[^ ^12^ ^]^ Considering a single mRNA could have multiple annotated localizations including nucleus, exosome, cytosol, ribosome, membrane, and endoplasmic reticulum (ER), the annotation of cytoplasm was merged with cytosol since these two terms were ambiguous. A previous published method, DM3Loc,^[^ ^12^ ^]^ provided a benchmark dataset containing 17 023 mRNAs, which has removed sequence redundancy (80% cutoff) by running CD‐HIT‐EST.^[^ ^83^ ^]^ The non‐redundant dataset was further partitioned into training and validation set for fivefold cross‐validation, with each fold having similar distribution of subcellular localization categories. The five folds of test data outside the training process were used to evaluate the prediction performance. For each mRNA transcript, the DM3Loc was followed to take the first 4000 nts at the 5' and the last 4000 nts at the 3' and concatenated them into a single sequence; for those mRNA transcripts shorter than 8000 nts, right‐padded with zeros. In addition to benchmarking against the previously annotated dataset provided by DM3Loc, an independent test was also conducted on mRNAs that had annotations in the updated RNALocate v2.0 database but were not included in the DM3Loc dataset.^[^ ^60^ ^]^ This novel dataset consisted of 2615 mRNAs; the detailed sub‐cellular locations could be found in Figure S16 (Supporting Information). After removing homologous sequences using CD‐HIT‐EST with 80% cutoff, a final set of 2551 sequences was derived.

### 3.6 Tokenize mRNA Sequence

An mRNA sequence is a string of nucleotides resembling human text; however, they lack explicit punctuation annotations to form discrete objects. Instead of considering each base as a single token, consecutive nucleotides were merged into *k*‐mers since functional motifs usually span multiple nucleotides.^[^ ^84^ ^]^ Given an RNA sequence such as “AUGACA,” the 3‐mers for this sequence consist of (AUG, UGA, GAC, ACA), or a sequence of two 5‐mers (AUGAC, UGACA). *k* was selected from 3 to 6 to generate token representations of different length. The final *k*‐mer vocabulary consists of a total of 4^*k*^ + 5 tokens including permutations of *k*‐mer and five special encoding symbols. Specifically, classification token [CLS] represents global contextual semantics of the entire sequence; separation token [SEP] represents the end of each input sequence; unknown token [UNK] represents all *k*‐mers that comprised at least one ambiguously sequenced character (e.g., “N”); masked token [MASK] represents the base fragment masked during self‐supervised learning process; and padding token [PAD] represents padding shorter input sequences to the desired length.^[^ ^31^ ^]^

### 3.7 Architecture of 3UTRBERT

3UTRBERT leverages deep representation learning to improve from task‐specific approaches to a more generalized and broad range of post‐transcriptional regulatory tasks. It uses pre‐training on the unannotated 3'UTR data (total 20 362 sequences and 76 435 649 nucleotides) to learn contextual information among nucleotides, akin to learning grammar and syntax of human language; these syntaxes or grammars in turn could reveal potential regulatory elements. The central component of 3UTRBERT architecture is the encoder part of a Transformer, which learns representations of the RNA universe via multi‐head self‐attention mechanism (please refer to Figure 1). First, the sequential segments of *k*‐mer linguistic tokens *t* = 〈*t*_1_, *t*_2_, …, *t*_*n*_〉 were fed into an Embedding layer ($E\in R^{n\times d}$), which independently converted each token *t*_*i*_ of vocabulary size *n* into a numerical vector with dimension *d* = 768. And the positional embedding was then summed with token embedding as the final input of the Transformer encoder network.

Twelve identical Transformer components were stacked as shared networks for mining regulatory characteristics hidden within 3'UTRs. Each layer contained a multi‐head self‐attention module and a position‐wise fully connected feed‐forward layer (FFN), with a residual connection around each sub‐layer followed by a layer‐wise normalization to greatly facilitate training. In particular, for the *k*‐mer token embedding matrix **X**, the dot‐product self‐attention coefficient was calculated as follows:

(1) $$\mathrm{Attention}\left(X\right)=W_{v}X\cdot \mathrm{Softmax}\left[\frac{{\left(W_{k}X\right)}^{T}\left(W_{q}X\right)}{\sqrt{d_{k}}}\right]=W_{v}X\cdot P$$

where $W_{q}\in R^{d_{q}\times d},W_{k}\in R^{d_{k}\times d}$ and $W_{v}\in R^{d_{v}\times d}$ represented the randomly initialized projection parameters associated with the query, key and value respectively. For each single‐head attention unit, *d*_*q*_ = *d*_*k*_ = *d*_*v*_ = *d* was used, and the matrix **P** was designed to capture the contextual relevance of the input for a given token against the remaining tokens in the input sequence. Subsequently, the information flow of weighted average was delivered to the formula below:

(2) $$\mathrm{LN}\left(X+W_{o}\cdot \mathrm{Attention}\left(X\right)\right)$$

Here LN(·) denoted the layer‐normalization operation and $W_{o}\in R^{d\times d}$. To jointly concern global dependencies from different representation subspaces at RNA tokens positions, multi‐head attention was independently performed for linearly project the queries, keys and values *h* times (number of heads = 12) in parallel. This was followed by concatenation of the output hidden state from *h* different heads, resulting in the final values as formulated:

(3) $$\;\text{head}\;{\left(X\right)}_{i}=W_{v}^{i}X\cdot \mathrm{Softmax}\left[{\left(W_{k}^{i}X\right)}^{T}\left(W_{q}^{i}X\right)/\sqrt{\frac{d}{h}}\right]\in R^{\frac{d}{h}\times n}$$

(4) $$\mathrm{Multi}\;\mathrm{Head}\left(X\right)=\;\text{Concat}\;\left[\mathrm{head}{\left(X\right)}_{1},\text{…},\;\text{head}\;{\left(X\right)}_{h}\right]\in R^{d\times n}$$

where the query, key and value projection matrices ${\left\{W_{i}^{Q},W_{i}^{K},W_{i}^{V}\right\}}_{i=0}^{h}$ were $\frac{d}{h}\times d$ matrices. Since each head reduced the dimension to compute the context, the total calculation cost was close to that of a full‐dimensional single‐headed attention. And the final multi‐head attentional context layer then became:

(5) $$Z=\mathrm{LN}\left(X+W_{o}\cdot \mathrm{Multi}\;\mathrm{Head}\left(X\right)\right),W_{o}\in R^{d\times d}$$

Following each attention layer, the discovered transcriptome contextual understanding passed through a fully connected feed‐forward network to each position separately and identically, which consisted of a single linear transformation with size *h* = 3072 and a GeLU activation:

(6) $$\mathrm{FFN}\left(x\right)=\mathrm{GeLU}\left(xW_{1}+b_{1}\right)W_{2}+b_{2}$$

Compared with the CNN‐based approaches which only captured the local context of nucleotide sequences, 3UTRBERT provided the flexibility to extract global dependency information from the entire input sequence. By performing the self‐attention mechanism on all the representations from the previous layer to calculate the hidden states, the model architecture could be easily parallelized; in addition, it could effectively overcome the gradient vanishing problem commonly encountered in RNN‐based methods.

### 3.8 Self‐Supervised Pre‐Training Stage

To empower the language model to learn the syntax and semantics embedded in 3UTRs, the Masked Language Model [MLM] pre‐training task was modified from the original deep representation learning implementation.^[^ ^21^ ^]^ Given that mRNA 3'UTRs were not composed of multiple sentences, the Next Sentence Prediction (NSP) task was discarded. The essence of MLM is a self‐supervised strategy of randomly masking a portion of an input 3'UTR sequence and then trains the network to predict their original values using the surrounding nucleotides. Specifically, 15% of the *k*‐mers was randomly masked, and the model was iteratively optimized to restore the masked parts based on the rest. To reduce mismatches between pre‐training and fine‐tuning stages, 80% of the masked token was replaced by a special token [MASK], 10% was randomly substituted with a *k*‐mer token from the vocabulary and another 10% remained unchanged from their original base. However, unlike human languages, the specific grammar of the *k*‐mer representation introduced a problem in token masking as a shielded *k*‐mer could be trivially inferred from immediate adjacent tokens. Taking a 3‐mer sequence (AUG, UGA, GAC, ACA, CAG) as an example, “GAC” was the concatenation of “GA” in “UGA” and “C” in “ACA.” If we masked 15% of the *k*‐mers randomly and independently, both the previous and the next *k*‐mers of the masked *k*‐mer would remain unmasked in most cases. To prevent the training task becoming simplified and thus affecting the learning of deep semantic relations, instead of masking each *k*‐mer independently, the *k*‐mer was masked for contiguous *k*‐length spans, i.e., (AUG, MASK, MASK, MASK, CAG). After this, by building a classification layer upon the last hidden state of the Transformer, the output probability of each *k*‐mer was calculated by a softmax function with cross‐entropy loss:

(7) $$L_{MLM}\left(\theta ,\theta_{MLM}\right)=-\sum_{i=1}^{V}\mathrm{rlogp}\left(v=v_{i}|\theta ,\theta_{MLM}\right)$$

where θ and θ_*MLM*_ respectively denoted the parameters of the 3UTRBERT pre‐training model and the MLM task layer (multi‐class classifier), and *V* equaled to the number of tokens in the vocabulary. In addition, different *k*‐mer length were experimented with in the model, as short *k*‐mer took shorter time to train than longer *k*‐mer but had less biological content; for example, 6‐mer model takes three times more to train than 3‐mer models. *k* was set as 3, 4, 5, and 6 to generate and evaluate four types of models. Each model followed the same architecture and parameter setting during pre‐training for about 38 days using four NVIDIA Quadro RTX 6000 24G GPUs. Parallel training technique was employed on four GPUs to support the large batch size of 128. 3UTRBERT was optimized following the training tricks described in previous publications^[^ ^22^, ^84^ ^]^ with AdamW (β_1_ = 0.9, β_2_ = 0.98, ϵ = 1*e* − 6), which iterated for a total of 200k steps. The learning rate was linearly increased (i.e., warm‐up) from 0 to 4e‐4 in the first 10k steps and then linearly decreased to 0 after 200k steps with weight decay rate set as 0.01.

### 3.9 Supervised Fine‐Tuning on Several Benchmarks

The agnostic pre‐training step allowed 3UTRBERT to learn the syntax and grammar encoded in the 3'UTR of the mRNA transcripts; next the model was fine‐tuned to perform several downstream tasks (Figure 1). It was showed that 3UTRBERT could transfer knowledge acquired in the pre‐training step to understand specific follow‐up post‐transcriptional regulatory activities. Some of the downstream tasks took longer mRNA sequences as input, e.g., predicting mRNA sub‐cellular localization using entire mRNA transcripts, whereas tasks such as predicting RBP binding sites or m6A sites took short sequence fragments as input. For long mRNA sequences, the parameters were frozen inside 3UTRBERT and then the vector representations were fed from the last hidden state (top right in Figure 1) to the model of multi‐label subcellular localization. It has been shown before that the representations on the feature‐dimension with mean pooling could prevent fusion bias caused by excessive dimensionality.^[^ ^24^ ^]^ In contrast, the short‐sequence fragments were initialized from the pre‐trained parameters and directly fine‐tuned with task‐specific data, which took much less time than pre‐training. We provided the aggregate sequence representation from the special token [CLS] of final hidden state into an output layer for target classifications, this layer was usually composed of a single‐layer feed‐forward neural network and softmax classifier. When experimenting 3UTRBERT with different *k*‐mers, identical hyperparameter settings and optimization tricks were used across all the applications, where AdamW fixed the weight decay (=0.01) and used dropout (=0.1) to alleviate the risk of overfitting. The learning rate was increased linearly to the peak value (=5e‐5) over the warm‐up period and decayed to near 0. Among evaluating different *k*‐mer sizes, the final results were reported for *k*‐mer = 3 since it achieved the best performance; only slight fluctuations on performance across different *k*‐mers was observed.

### 3.10 Visualization of 3UTRBERT and Motif Extraction

The rationale of 3UTRBERT was to identify nucleotide sequences that have high attention scores, which suggest that these nucleotides are potentially under functional constraints, e.g., binding sites for RBPs or m6A modification sites. Leveraging the self‐attentive mechanism, 3UTRBERT was naturally applicable for mapping and deciphering regulatory regions in 3'UTRs. The special classification token [CLS] in first position of the input represents the aggregated representation from the last output layer;^[^ ^21^ ^]^ the contribution score of each embedded *k*‐mer token α_*j*_ accumulated over all attention heads *H* in the context of “entire sequence” was calculated following the formula below:

(8) $$\alpha_{j}=\sum_{h=1}^{H}\frac{\exp\left(q^{*T}k_{j}/\sqrt{d}\right)}{\sum_{l=1}^{J}\exp\left(q^{*T}k_{l}/\sqrt{d}\right)}$$

where q* denotes the query vector of [CLS] token with the dimension of *d*; *k*_*j*_ denotes the key vector for the *j*‐th *k*‐mer token; and *J* was the number of tokens within input sequence. Next, Dodrio^[^ ^72^ ^]^ was employed to track relationships within nucleotide sequences, such as contextual semantics and subsequent meaning between different *k*‐mers. The Attention Head Overview provides a comprehensive analysis of the different attention heads in Transformer‐based models, offering insights into why they perform well on certain tasks. To convert the attention score from *k*‐mer to individual nucleotides, for a particular sequence, we averaged the values of all the *k*‐mers were averaged that the nucleotide was part of. Meanwhile, the Shapely Additive Explanations (SHAP) and Integrated Gradients (IG) explainers were also used to verify the contribution of each position on each *k*‐mer to the overall prediction value of the RNA sequence.^[^ ^76^, ^77^ ^]^ After having identified sequence regions of high attention scores, next it was evaluated whether they were enriched in RBP binding sites or m6A modification sites. The extracted attention weights from [CLS] to each token (α_*CLS*, *j*_) were used to identify high‐attention regions in each positive sequence; sequence motif candidates were identified with 7‐mer length.^[^ ^9^ ^]^ Then hypergeometric test was used to calculate the statistical significance of the enrichment of these identified motifs and conducted Benjamini–Hochberg multi‐testing correction with adjusted *p*‐value < 0.005.^[^ ^85^ ^]^ Finally, pairwise alignment was performed to merge similar motif candidates, the resulting instances with the highest numbers were converted into a position‐weight matrix (PWM) format. These motifs were then compared with the experimentally validated motifs stored in ATtRACT database^[^ ^36^ ^]^ using TOMTOM algorithm.^[^ ^37^ ^]^

## 4 Discussion

The 3'untranslated regions (3'UTRs) of mRNAs are essential for post‐transcriptional gene regulation, which are conferred by evolutionarily conserved (and in some cases less conserved) sequence or structure elements. We hypothesize that these sequence motifs have evolved in a way like human languages, following specific grammar and syntax to confer required biological roles.^[^ ^19^ ^]^ Natural language models have recently been applied to predicting protein 3D structures with great success.^[^ ^27^, ^28^ ^]^ Motived by this, we developed 3UTRBERT, which is a Transformer‐based framework that can be pre‐trained on large body of 3'UTR sequences, learn the semantics and grammar, and be further fine‐tuned for specific biological tasks such as predicting protein‐RNA binding, RNA modifications, and RNA sub‐cellular localizations. Benchmark studies against other state‐of‐the‐art methods for these individual tasks demonstrated that 3UTRBERT generally outperformed or performed equally well. We believe the 3UTRBERT model, and other similar nature language‐based models, have the following major advantages. Once trained on a large amount of data and learned the intrinsic structure and syntax of the data, it is quite flexible to adapt to specific subtasks with minimum fine‐tuning. The pre‐training step can be computationally expensive, but it only needs to be performed once. The pre‐training step is also un‐biased without the need of labeling, which is in contrast to most other probabilistic or rule‐based approaches (the fine‐tuning step does require labeled datasets).

There are several avenues in which 3UTRBERT can be improved or extended. We only trained the model on 3'UTRs from human genes since the 3'UTR plays a crucial role in post‐transcriptional regulation, including mRNA stability, translation efficiency, and subcellular localization. It contains numerous regulatory elements such as RBP binding sites, AU‐rich elements, and polyadenylation signals, which are essential for these regulatory processes, thus our model was specifically designed to interpret these regulatory features. Indeed, the 5'UTR and CDS are also important, but their length and additional regulatory elements increase the complexity. The current 3UTRBERT model can handle sequences of up to 510 nucleotides, but the length of CDS may be much longer than this limit (as described in the Experimental Section). Truncating the CDS destroys their native sequence context, resulting in loss of key information and degradation of model performance. Moreover, the regulatory mechanisms mediated by the 5'UTR and CDS are distinct from those mediated by the 3'UTR. For instance, the 5'UTR is involved in the initiation of translation and can contain upstream open reading frames (uORFs) that regulate translation efficiency. The CDS encodes the protein sequence and can influence translation elongation and co‐translational folding. Integrating these distinct regions would require sophisticated models capable of handling diverse regulatory signals and interactions, which was beyond the scope of our current computational resources. We acknowledge that utilizing both the 5'UTR and 3'UTR regions could enhance the model's ability to predict comprehensive regulatory features. This is an area we plan to explore in future work, i.e., optimizing our computational resources and model architecture to accommodate longer sequences and cover multiple regions of the mRNA. Also, it would be intriguing to include 3'UTRs from other evolutionarily related mammalian or vertebrate species as well. It is reasonable to assume that increased training data will improve prediction accuracy and reveal more subtle sequence features. We will need to experiment with different threshold to investigate the necessity and effect of including or removing 3'UTR from orthologous genes from related species. It is also worthwhile to investigate whether 3UTRBERT or natural language models can effectively discover lineage specific sequence signals, which could be “buried” when large corpus of sequence data from many species is used in the pre‐training stage. Lastly, there is a still relatively paucity of experimentally determined functional data on RNAs, except for in vitro RBP‐RNA binding affinities and selected in vivo RBP‐RNA binding events. With RNA related data becoming increasingly available in the coming years, we expect 3UTRBERT or other similar nature language‐based models will become more effective in training and discovering informative RNA sequence features.

## Conflict of Interest

The authors declare no conflict of interest.

## Supporting information

Supporting Information

## Data Availability

The data that support the findings of this study are openly available in 3UTRBERT_dataset_availability at https://doi.org/10.6084/m9.figshare.26082916.v1.
