# Supplementary material for: Deciphering 3'UTR Mediated Gene Regulation Using Interpretable Deep Representation Learning
Source: Adv Sci (Weinh). 2024 Aug 19;11(39):2407013. doi: 10.1002/advs.202407013 (PMC11497048; doi:10.1002/advs.202407013)
Supplement: Supplementary file 1 — Supporting Information [file ADVS-11-2407013-s001.pdf]

ADVANCED SCIENCE
Open Access

Supporting Information

for *Adv. Sci.*, DOI 10.1002/advs.202407013

Deciphering 3’UTR Mediated Gene Regulation Using Interpretable Deep Representation Learning

Yuning Yang, Gen Li, Kuan Pang, Wuxinhao Cao, Zhaolei Zhang and Xiangtao Li**

Supplementary Information 'Deciphering 3'UTR Mediated Gene Regulation Using Interpretable Deep Representation Learning'

Yuning Yang[1,2] *Gen Li*[2] *Kuan Pang*[2] *Wuxinhao Cao*[2] *Zhaolei Zhang*[2,3,4,*] *Xiangtao Li*[5,*]

1 School of Information Science and Technology, Northeast Normal University, Changchun, Jilin, 130117, China.
2 Donnelly Centre for Cellular and Biomolecular Research, University of Toronto, Toronto, ON, M5S 3E1, Canada.
3 Department of Computer Science, University of Toronto, Toronto, ON, M5S 3E1, Canada.
4 Department of Molecular Genetics, University of Toronto, Toronto, ON, M5S 3E1, Canada.
5 School of Artificial Intelligence, Jilin University, Changchun, Jilin, 130012, China.

*Corresponding authors:
Z.L. Zhang
Email Address: zhaolei.zhang@utoronto.ca
X.T. Li
Email Address: lixt314@jlu.edu.cn

1 Definition of the evaluation metrics

Short name	Full name	Formula
ACC	Accuracy	ACC = $\frac{\mathrm{TP+TN}}{\mathrm{TP+FP+FN+TN}}$
AUROC	Area Under the Receiver Operating Characteristic	ROC AUC is the area under the curve where x is false positive rate(FPR) and the y is true positive rate(TPR).
AUPRC	Area Under the Precision-Recall Curve	PR AUC is the area under the curve where x is recall and y is precision.
MCC	Matthews correlation coefficient	MCC = $\frac{TP*TN-FP*FN}{\sqrt{(TP+FP)*(TP+FN)*(TN+FP)*(TN+FN)}}$
F1	F1-measure (harmonic mean of precision and sensitivity)	F1 = $\frac{2*PPR*TPR}{PPR+TPR} = \frac{2*TP}{2*TP+FP+FN}$
Precision	Precision	Precision = $\frac{TP}{TP+FP}$
Recall	Recall	Recall = $\frac{TP}{TP+FN}$
TPR	True positive rate(sensitivity)	TPR = $\frac{TP}{TP+FN}$
FPR	False positive rate	FPR = $\frac{FP}{TN+FP}$
PPR	Predicted positive rate(precision)	PPR = $\frac{TP}{TP+FP}$
TP	True positive	Number of correctly predicted crosslink/modification sites.
TN	True negative	Number of correctly predicted non-crosslink/non-modification residues.
FP	False positive	Number of non-crosslink/non-modification residues incorrectly predicted as crosslink/modification.
FN	False negative	Number of crosslink/modification residues incorrectly predicted as non-crosslink/non-modification.

For sequence labelling tasks of binary classification, the predictions were generated from the propensities such that nucleotides with propensities greater than a given threshold are identified as crosslinked/modified, and otherwise they are identified as non-crosslinked/non-modified. We evaluated the predictive performance of the binary identifications with the four metrics and thresholds as 0.5: Accuracy, F1, and MCC (Matthews correlation coefficient). Accuracy showed us how comfortable the model was with detecting the positive and negative classes. It was computed by the sum of True Positives and True Negatives divided by the total population. F1 ranged between 0 and 1 where higher value denoted more accurate prediction. MCC ranged between −1 and 1, where −1 represented an inverted prediction (all predictions were flipped compared to the experimental values), 0 denoted a random result and 1 denoted a perfect prediction. The area under the receiver operating characteristic (AUROC) curve to evaluate discriminate quality of the propensities. The AUROC curve was a relation between true positive rates (TPRs) and false-positive rates (FPRs) that was calculated by thresholding the propensities where the thresholds were the set of all unique propensities produced by a given predictor. ROC-AUC ranged between 0.5 (equivalent to a random identification) and 1 (perfect identification). To support multi-label classification, the estimator was wrapped in a OneVsRestClassifier to produce binary comparisons for each class (e.g. the positive case is the class and the negative case is any other class). The precision-recall curve (AUPRC) showed the tradeoff between precision and recall for all classes. The AUPRC curve provided a more accurate assessment of the model's performance than metrics such as accuracy or F1 score, which might be biased towards the majority class.

2 RNA-protein docking and molecular dynamics simulation

In this study, the 3D structures of the filtered RNA sequences were modelled with the DeepFoldRNA software [1], a stand-alone program obtained from the official release at https://github.com/robpearc/DeepFoldRNA. Commencing with an RNA sequence, the program generated a homology-based alignment of numerous sequences sourced from diverse databases (as of March 2023). Subsequently, the software employed deep neural networks to predict spatial restraints, which were transformed into negative log-likelihood potentials. Finally, L-BFGS folding simulations were executed to produce a full-length structure model. Since no experimentally determined structure was available for RBM15, we resorted to computational methods for structure prediction. Specifically, we obtained the protein structure of RBM15 from AlphaFoldDB, a publicly database of protein structures generated by the AlphaFold algorithm [2]. We retrieved the protein structure from AlphaFoldDB (entry Q96T37) and selected the RNA Recognition Motif (RRM) to build the molecular dynamics (MD) system. Crystal structure for SND1 was retrieved from PDB entry 5M9O, and further utilized to perform the analysis. The ClusPro [3] web server was subsequently employed to model the RNA-protein docking, and the populated binding pattern was sent to the MD simulation for evaluating the stability and dynamics. After that, the Charmm-gui webserver was applied to construct the MD systems [4, 5, 6]. The RNA-protein complex was solvated in a water box with a 15 Å buffer around it and the system was neutralized with K+ and Cl- ions at 0.15M concentration. The systems were equilibrated and energy-minimized for 10,000 steps in the NPT ensemble. We then stabilized the temperature at T = 30°C with the Nosé-Hoover method [7] and the coupled pressure with the Parrinello–Rahman method [8]. A switch distance of 10-Å and a cutoff distance of 12-Å for non-bonded interactions were applied. Bond lengths were further constrained with hydrogen atoms using LINCS [9] with a 2-fs time step, and the systems varied in the number of atoms from 216,000 to 332,000. We finally performed energy minimization and production MD simulations with GROMACS (2021.6) [10], using CHARMM36m force field [11] and TIP3P water model [12].

Supplementary Figure 1

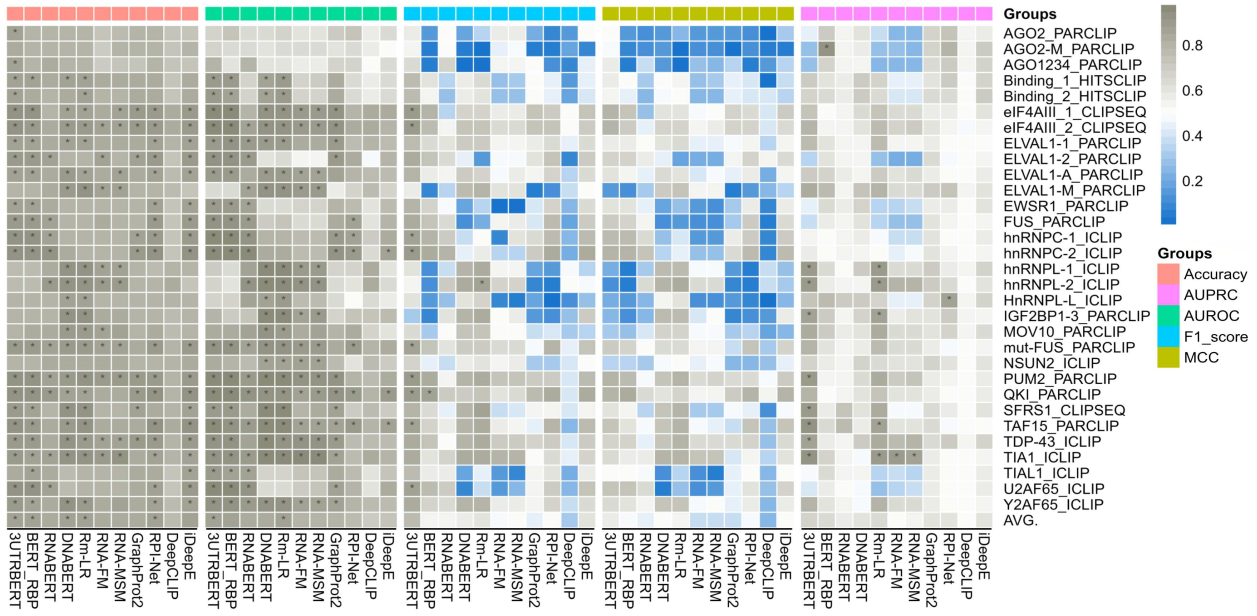

Supplementary Fig. 1: The heatmap plot of five evaluation criteria to demonstrate the robustness among RNA-RBP classification baselines on CLIP/HITS-CLIP, iCLIP and PAR-CLIP from hg19.

Supplementary Figure 2

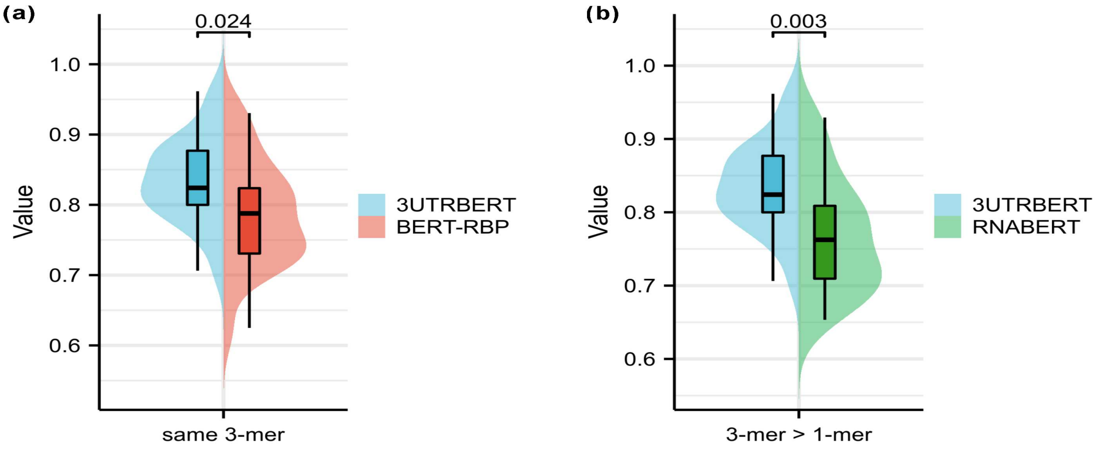

Supplementary Fig. 2: Comparison results of Transformer architecture-based approaches for 22 RBPs under eCLIP protocol. (a) At the same token length, 3UTRBERT injected with prior knowledge of the regulatory regions was superior to BERT_RBP, which was directly fine-tuned on the human reference genome and lacked the pre-training stage, in identifying RNA-protein cross-linking stable sites. (b) Since none of the regulatory motifs in the non-coding region occurred at single resolution, RNABERT thus suffered from a performance bottleneck caused by the loss of local contextual information. More importantly, RNABERT had difficulty in modelling the relationship between UTR and target protein expressions based on the implicit representation learned by non-coding RNAs.

Supplementary Figure 3

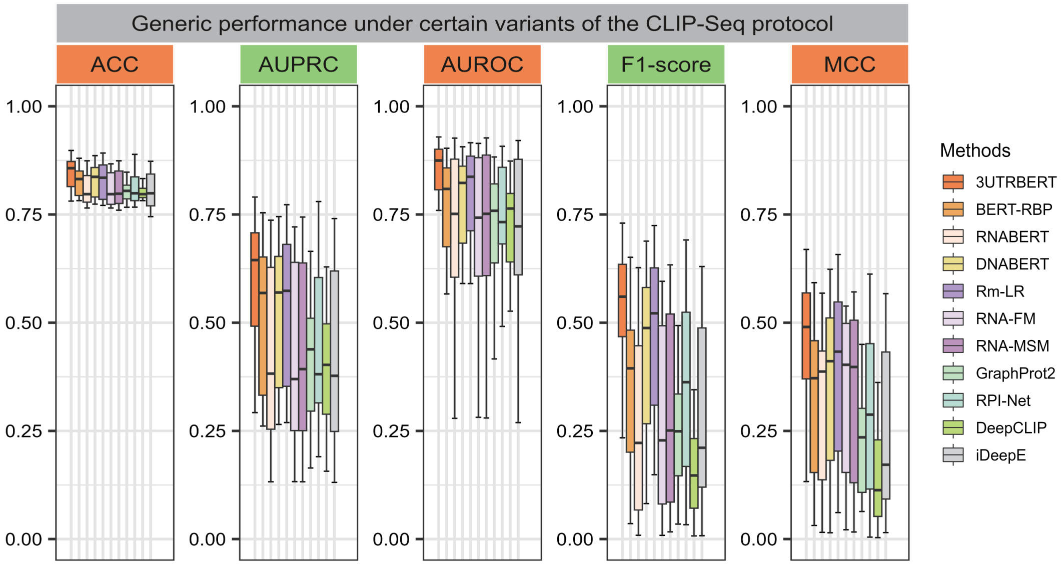

Supplementary Fig. 3: Generalizability comparison with the state-of-the-art methods in 31 CLIP experiments on 19 RBPs. 3UTRBERT-generic model achieved the best performance compared to other methods (AUROC = 0.829, AUPRC = 0.585, F1 = 0.514, MCC = 0.438, and ACC = 0.853), while the second-best results were AUROC = 0.798 (by Rm-LR), AUPRC = 0.527 (by Rm-LR), F1 = 0.467 (by Rm-LR), MCC = 0.381 (by Rm-LR) and ACC = 0.835 (by DNABERT).

Supplementary Figure 4

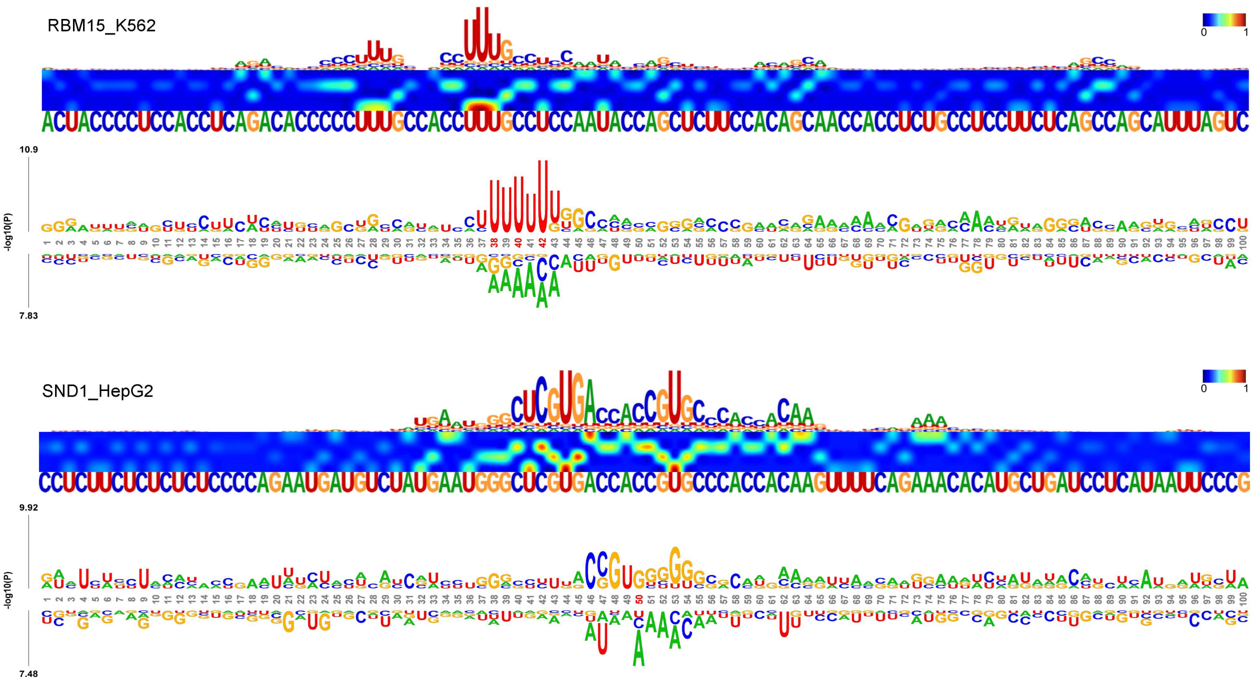

Supplementary Fig. 4: 3UTRBERT can effectively identify RBP target signals on RNA sequences through the self-attention mechanism, which is consistent with the statistics of pairwise enrichment of each position-specific 3-mer calculated by kpLogo.

Supplementary Figure 5

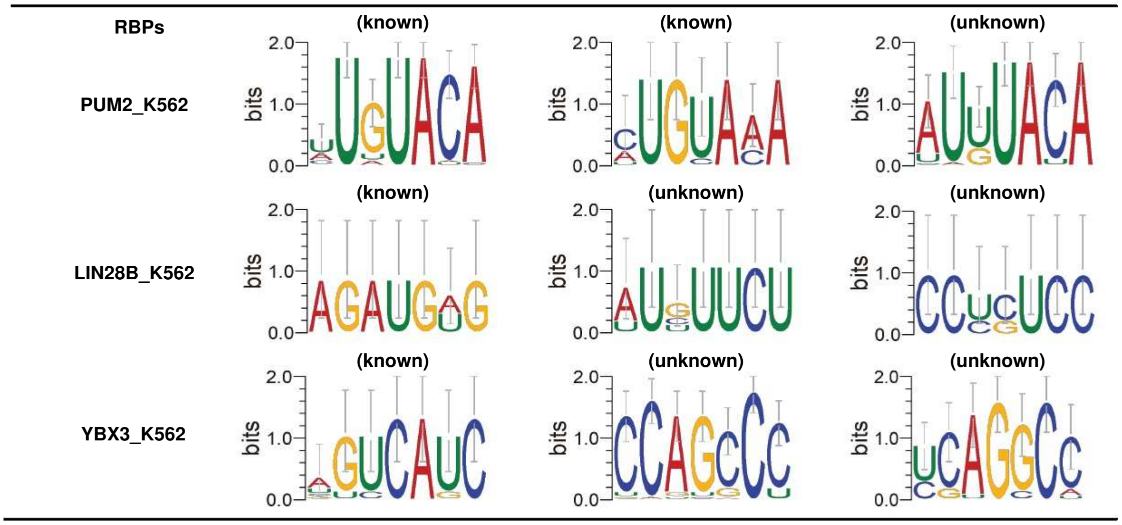

Supplementary Fig. 5: Significant RNA binding signals discovered by 3UTRBERT on PUM2, LIN28B, and YBX3 proteins. In addition to matching recorded motifs, 3UTRBERT also learns other sequence patterns to reduce false positive hits.

Supplementary Figure 6a

Tertiary structure of RBM15 binding RNA

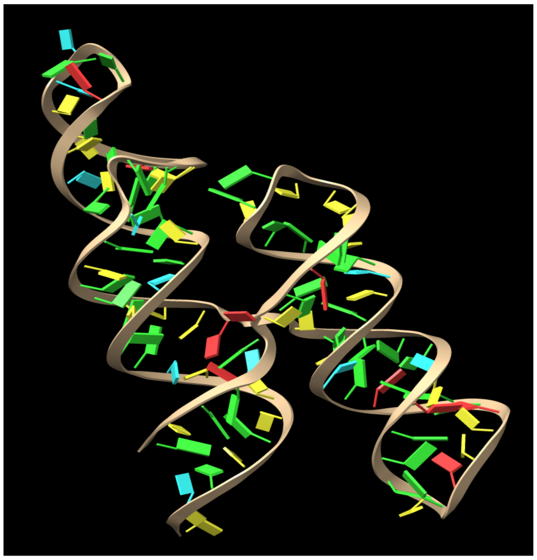

>chr5:134905267-134905367
GCGGUGCGA**GGCCCGG**CGGCGUCGGCUGAGGGACCAGCGGGACU
GGGGCGAACCCGGCGUGGGCCGAGCCUUGGAGCUCGGGCGUCGG
GUCACCGCGUCC

Tertiary structure of RBM15 protein

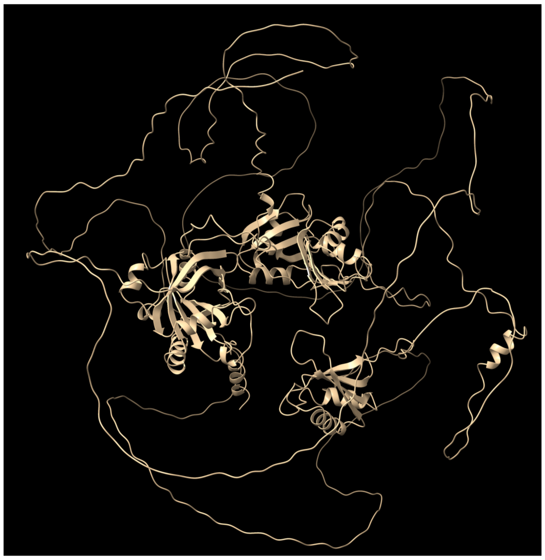

7Z27_Chain A (Q96T37)

Tertiary structure of SND1 binding RNA

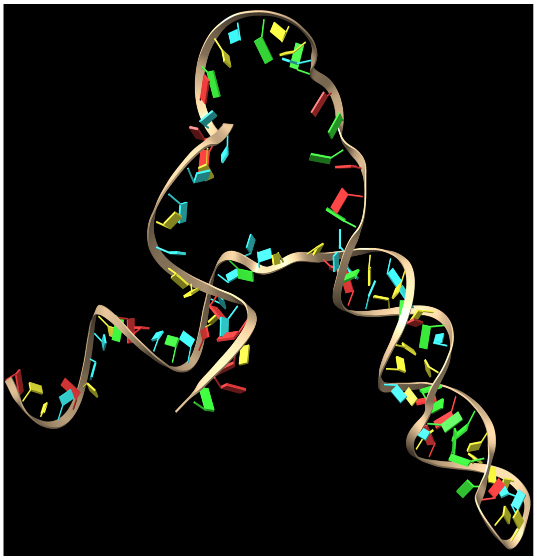

>chr12:52900579-52900679
GAACAUACCUCUUCAUAUAGCUGCCUGAGGAAGUUGAUCUCGUCG
GUCAGCCC**UUCCAGG**CGAGACUCCAGCUCUACCUUGUUCAUGUAA
GCUUCAUCCA

Tertiary structure of SND1 protein

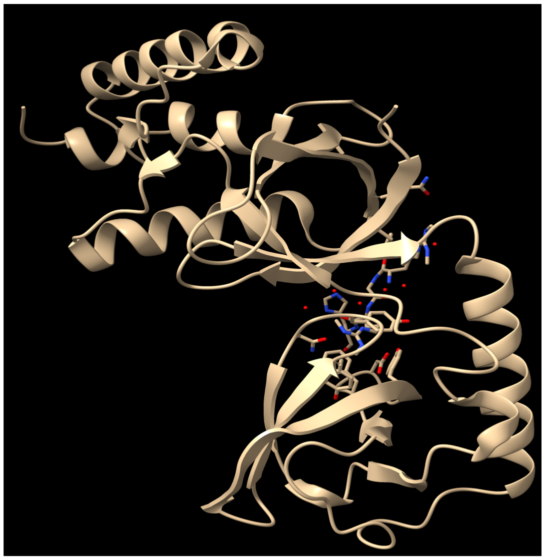

5M9O_Chain A (Q7KZF4)

Supplementary Figure 6b

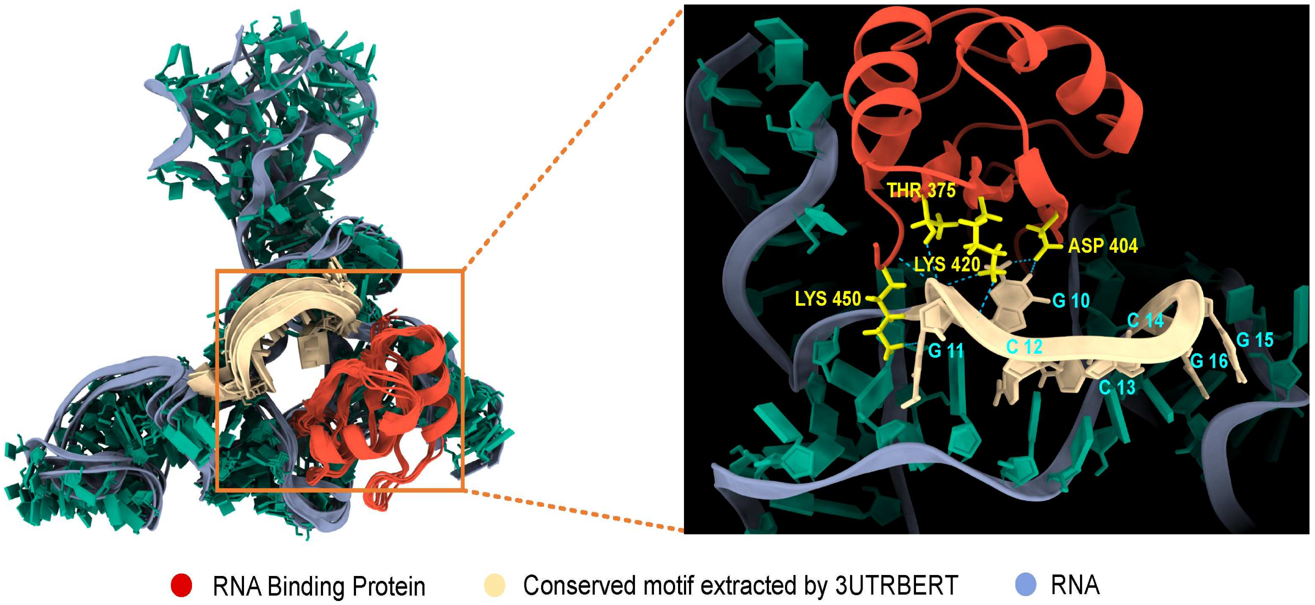

Supplementary Fig. 6: (a) The tertiary structure of RBM15 and SND1 proteins with corresponding binding RNAs, identified motifs are shown in red with specific sequence information. (b) Predicted complex structure between RBM15 and a target RNA sequence (GGCCCG), after protein-RNA docking and all-atom molecular dynamics simulations. Hydrogen bonds between side chain residues of RBM15 and RNA are observed: Thr375-G11, Asp404-G10, Lys420-G10/G11, and Lys450-G11.

Supplementary Figure 7

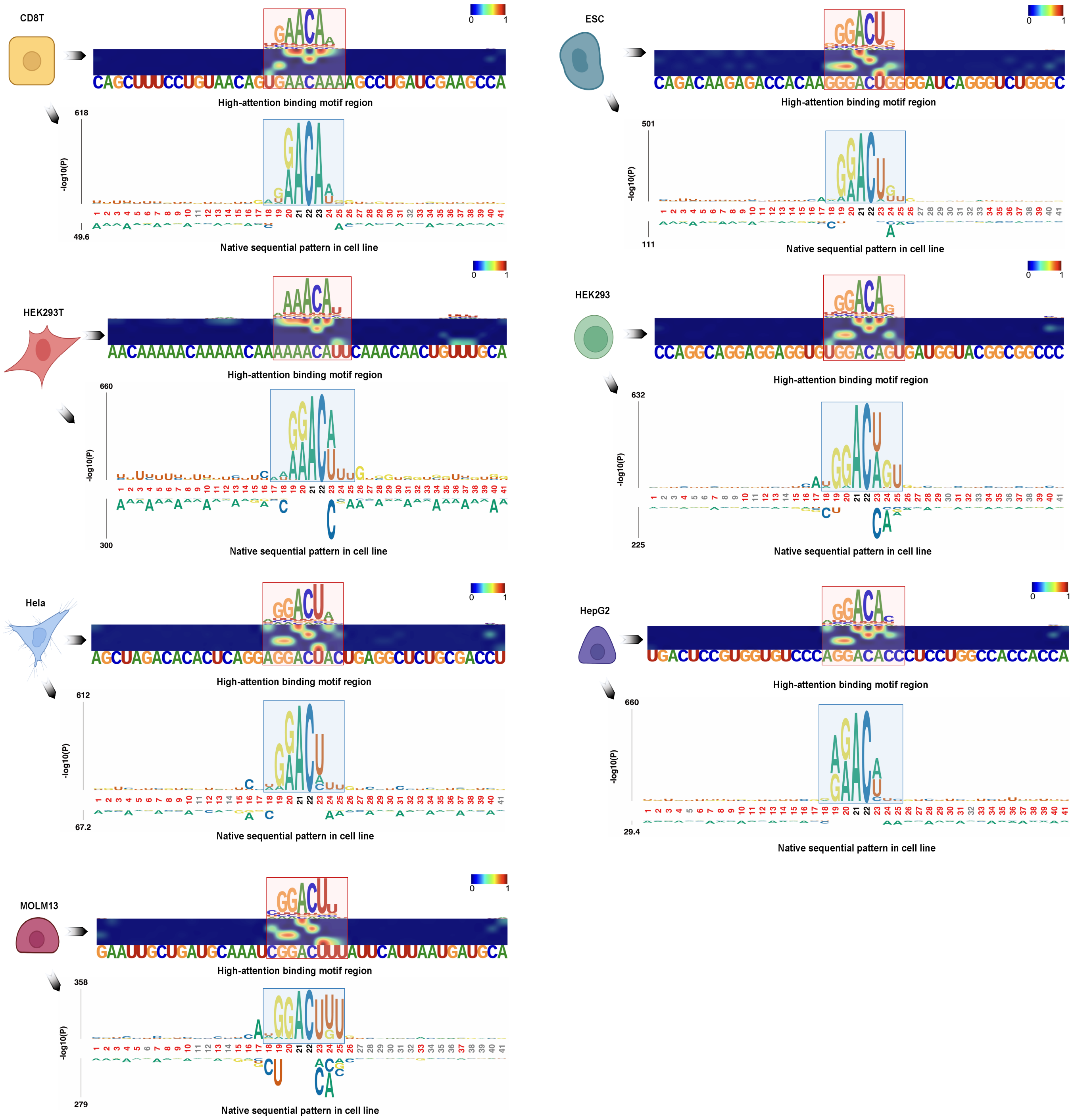

Supplementary Fig. 7: 3UTRBERT explored the consensus patterns of sequential information across various cell lines, thus equipping superior generalizability to dynamically identify cellular epitranscriptomic modifications.

Supplementary Figure 8

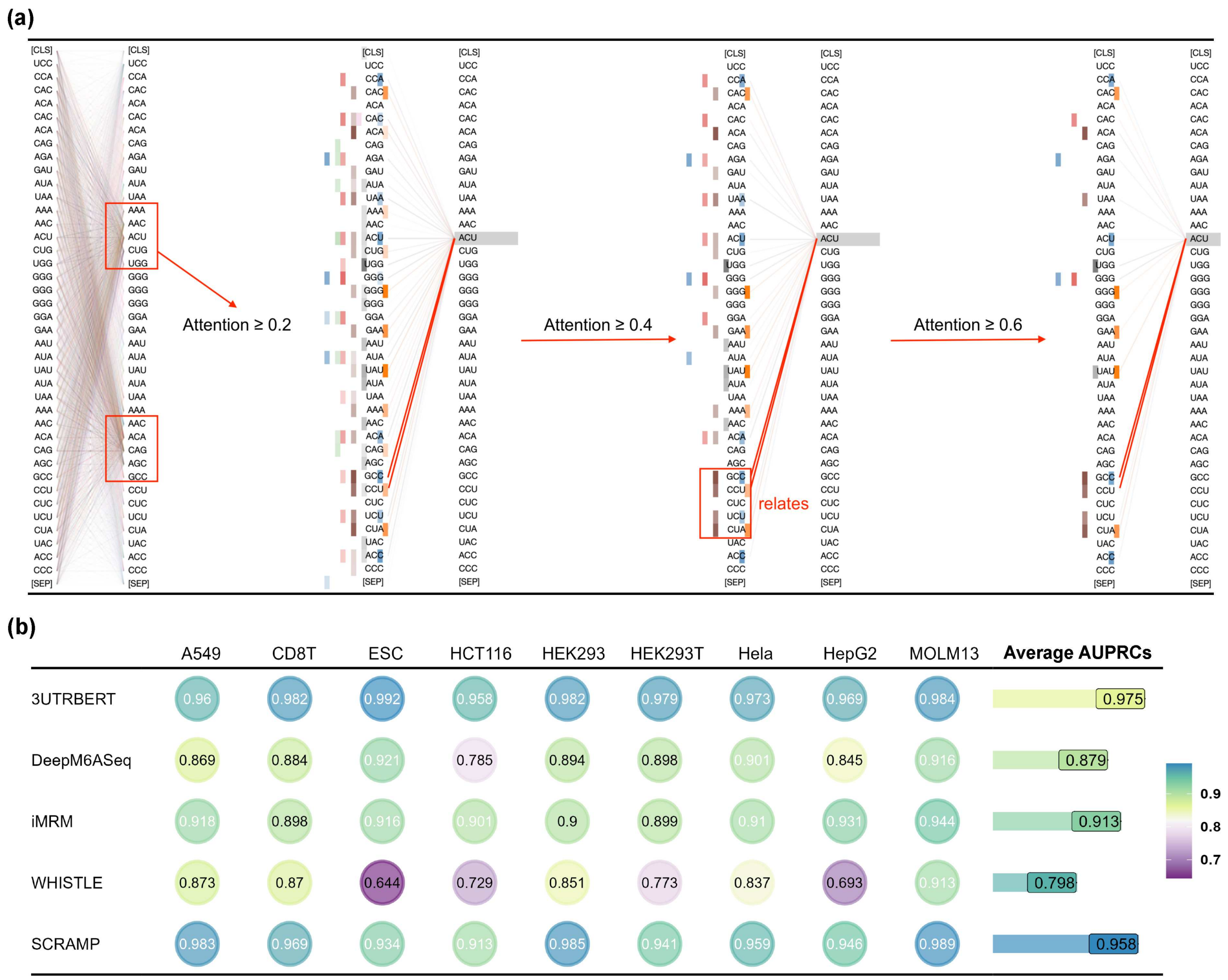

Supplementary Fig. 8: (a) The contextualization plots of a m6A modification site, showing the attention-heads (heatmaps on the left) of the 'ACU' token at sentence level, with self-attention thresholds of 0.2, 0.4, and 0.6 respectively. (b) The bubble chart shows the performance comparison of different methods on non-redundant genetic data (measured by AUPRCs).

Supplementary Figure 9

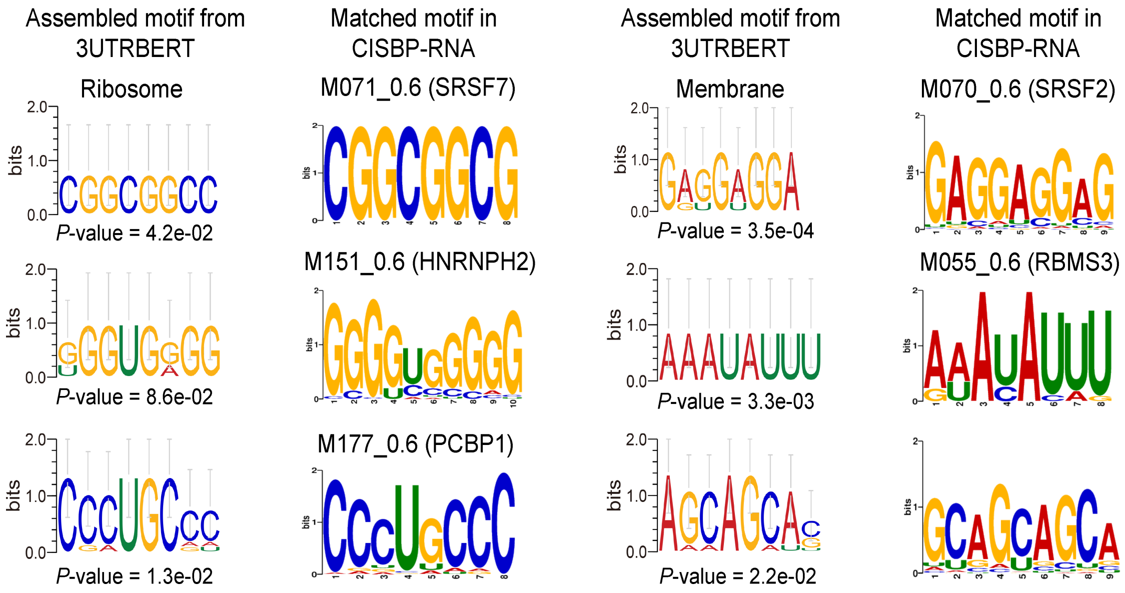

Supplementary Fig. 9: Visualization of class-specific sequence motifs mapped to the known targeting signals from CISBP-RNA database.

Supplementary Figure 10

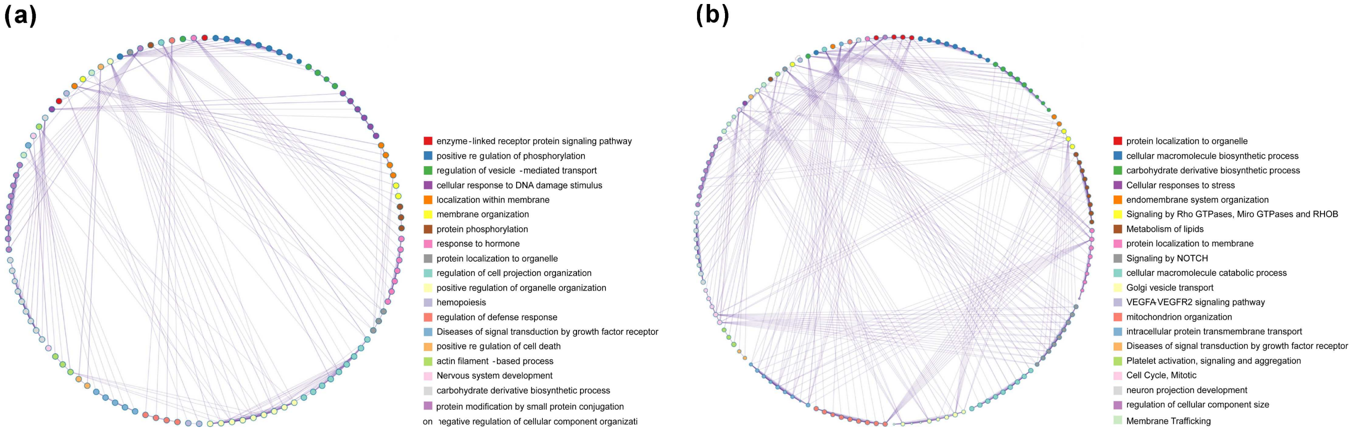

Supplementary Fig. 10: The Gene Ontology Network respectively displayed the relationships contained in Endoplasmic Reticulum (right) and the sets composed of nucleus, exosome, cytosol, and membrane (left) on the independent test set. The contextual semantics embedding generated by 3UTRBERT yielded better performance for ER compartment with more abundant background information.

Supplementary Figure 11

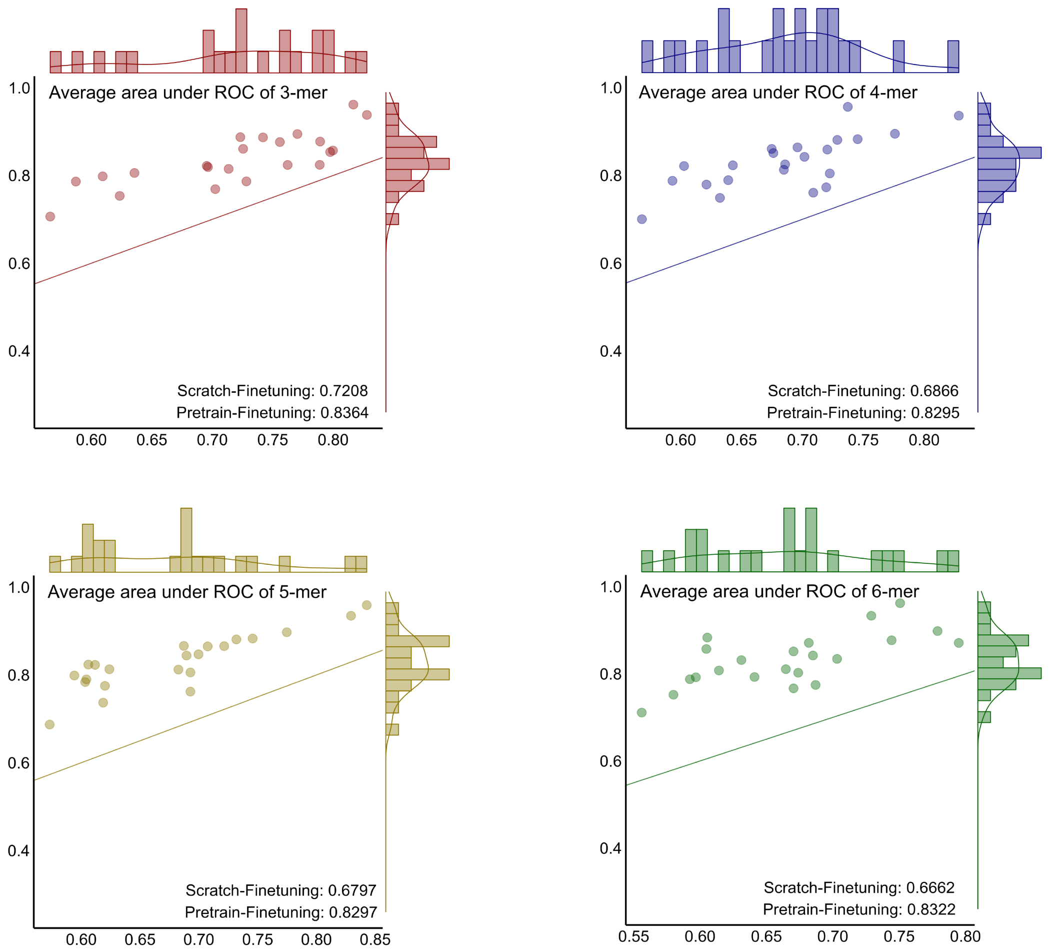

Supplementary Fig. 11: Performance comparison on 22 RBPs between the stage of scratch-finetuning and pretrain-finetuning in terms of AUCs to demonstrate that the task-agnostic self-supervised learning approach captured generalized and transferable understanding from regulatory regions compared with random initialization information.

Supplementary Figure 12a and 12b

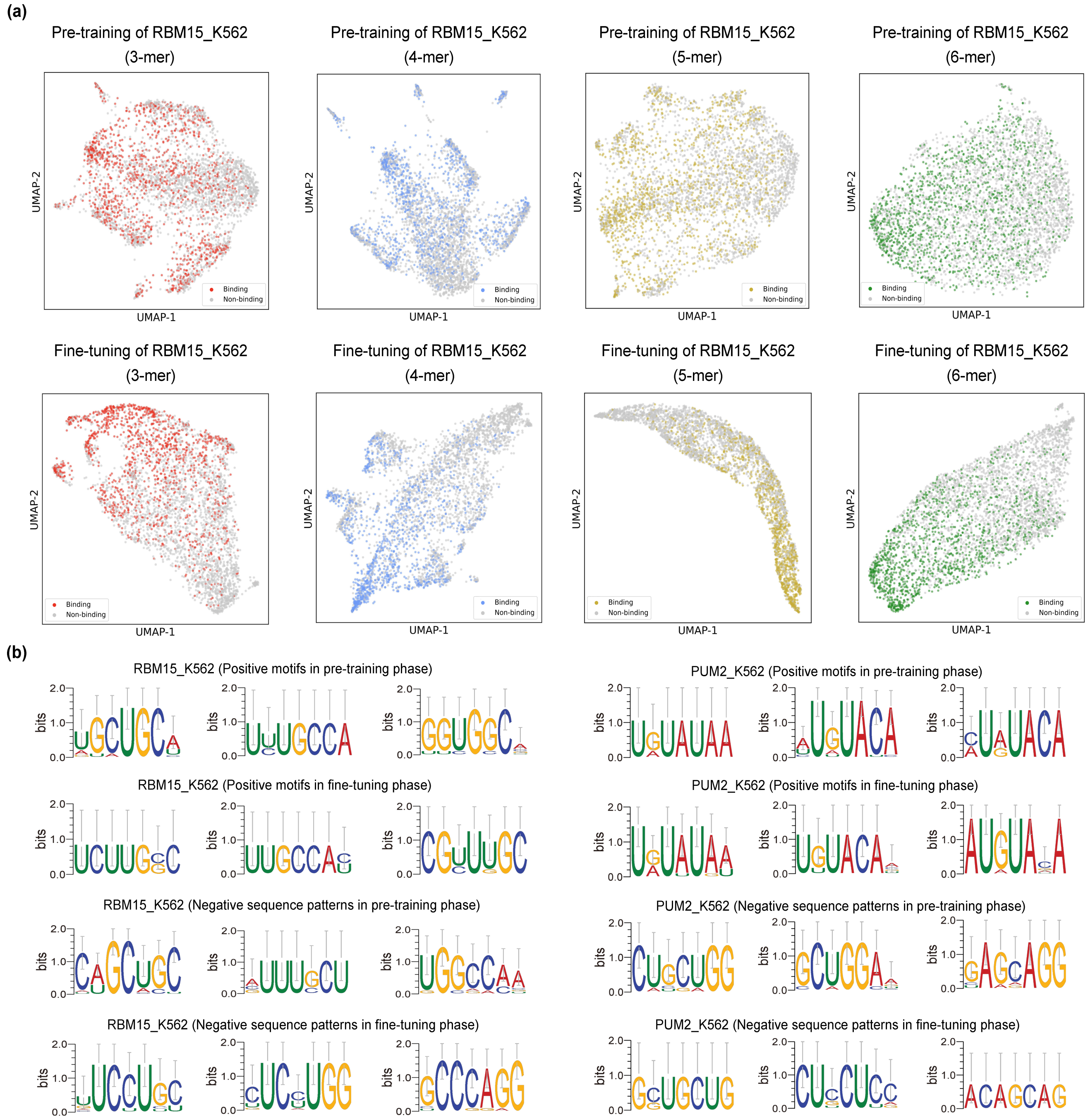

Supplementary Fig. 12: (a) Use UMAP projection to visualize the clustering effect of 3UTRBERT's embedding representation on RBM15 proteins, including pre-training and fine-tuning stages (k-mer size = 3, 4, 5, 6). (b) The potential sequential patterns extracted by 3UTRBERT from the positive and negative samples of RBM15 and PUM2 protein during the pre-training and fine-tuning stages.

Supplementary Figure 12c and 12d

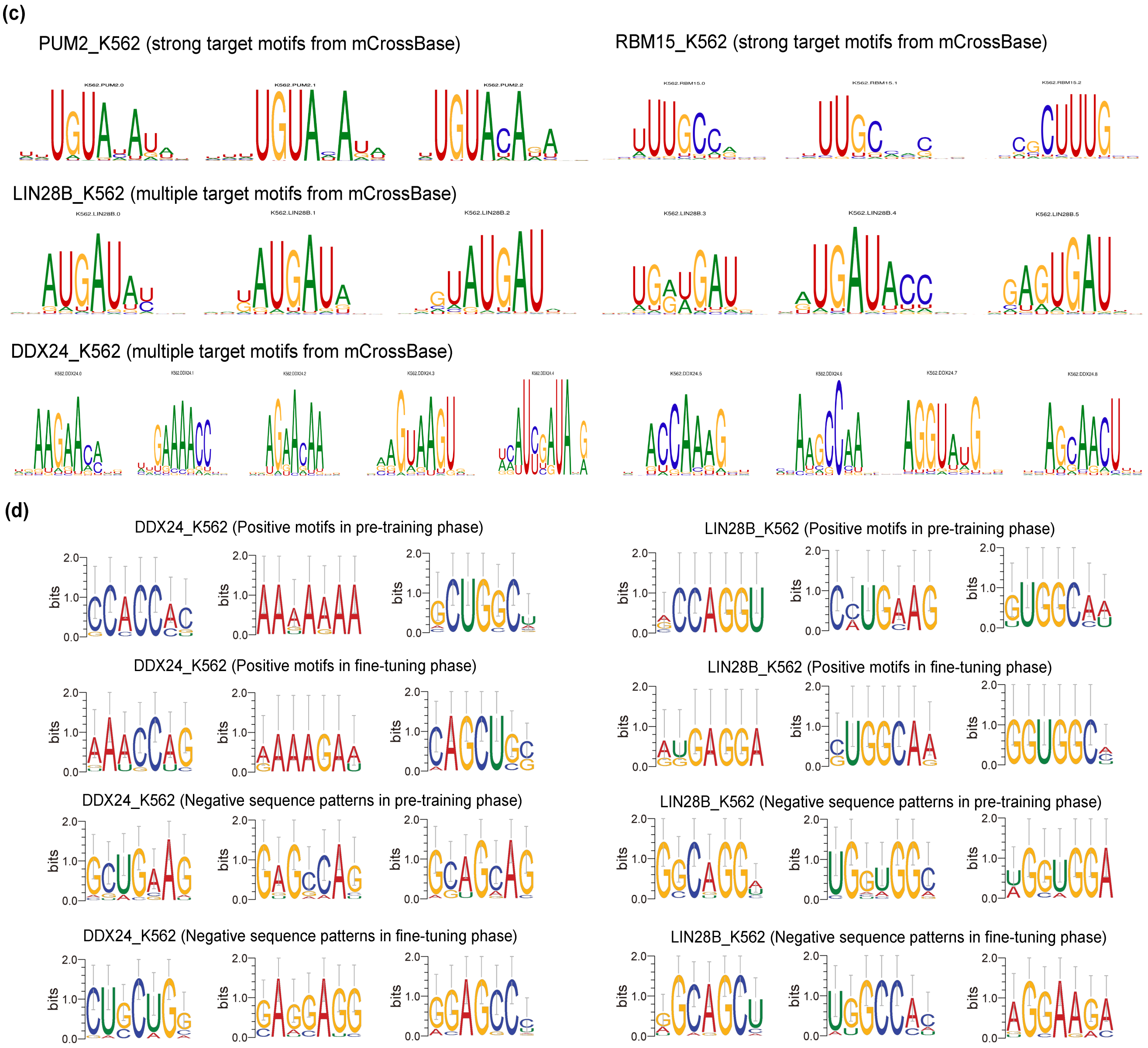

Supplementary Fig. 12. (c) The experimentally target signals of different RBPs documented in the mCrossBase database, RBM15 and PUM2 proteins have specific binding motifs, while LIN28B and DDX24 have more motif types, which require further learning by 3UTRBERT to enhance prediction. (d) Benefiting from the fine-tuning stage, DDX24 and LIN28B proteins gradually matched the known motif patterns in mCrossBase, resulting in more obvious changes on the attention maps.

Supplementary Figure 13

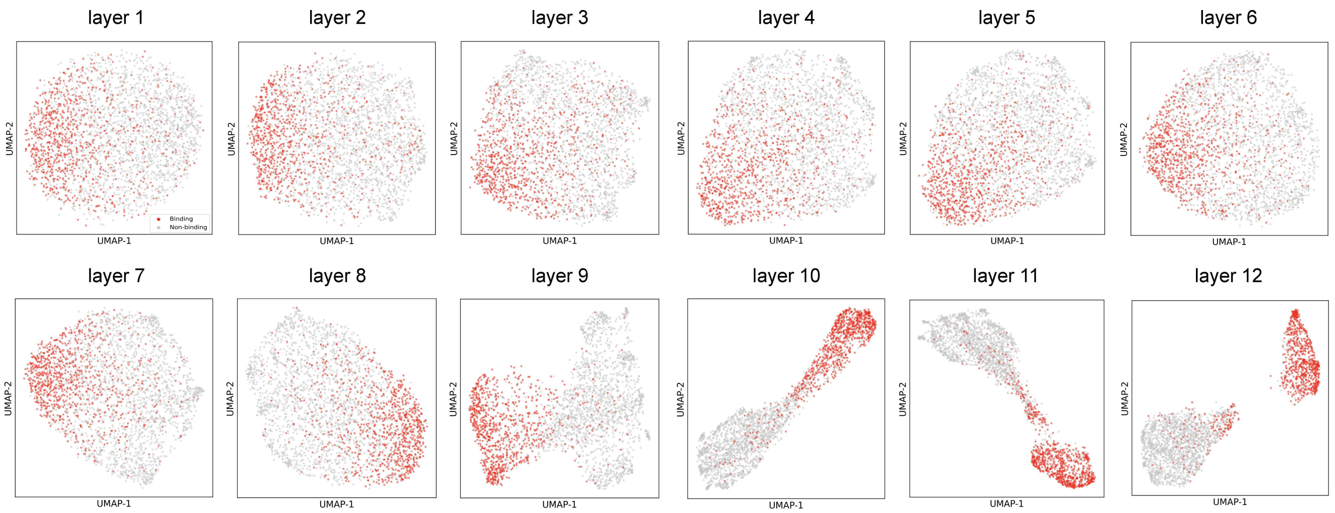

Supplementary Fig. 13: A two-dimensional projection of the embedding vectors generated by 3UTRBERT using UMAP to visualize the clustering effect of each layer on RNA sequences; linkage sites (positive samples) were annotated in colors while non-linkage sites (negative samples) with grey color. As moving up the layers in language model, the representations become increasingly contextualized, which make them more effective for classification tasks.

Supplementary Figure 14

	aopc_compr	aopc_suff	taucorr_loo
Partition SHAP	0.36	0.25	0.30
LIME	0.33	0.34	0.24
Gradient	0.32	0.42	0.12
Integrated Gradient	0.38	0.30	0.28

>chr2:65313675-65313775
UAGUCUCCCUCGGGGUCCGACAUACAGUGAU
AGAGCAUGCUGUCCGCGCACCACAUGCAGCU
CACCCGGCGGAUGCAAGUUCUCACGGAGUCG
GGCGCG

	aopc_compr	aopc_suff	taucorr_loo
Partition SHAP	0.40	0.18	0.34
LIME	0.40	0.32	0.19
Gradient	0.27	0.29	0.11
Integrated Gradient	0.44	0.26	0.29

>chr5:132825818-132825918
UCAGUGGUCCCGGGGUCCCCGGGACCUCUG
UUGGCUGCGGCCCACUGCGGGCUGCAACCG
CGGGCCGGGGCCGCGGGGAUGUGCAAAGGG
CAGCGUCGG

Supplementary Fig. 14: Using Ferret to show that SHAP and Integrated Gradients best follow the internal mechanisms of the Transformer model across different explainers, leading to better interpretability.

Supplementary Figure 15

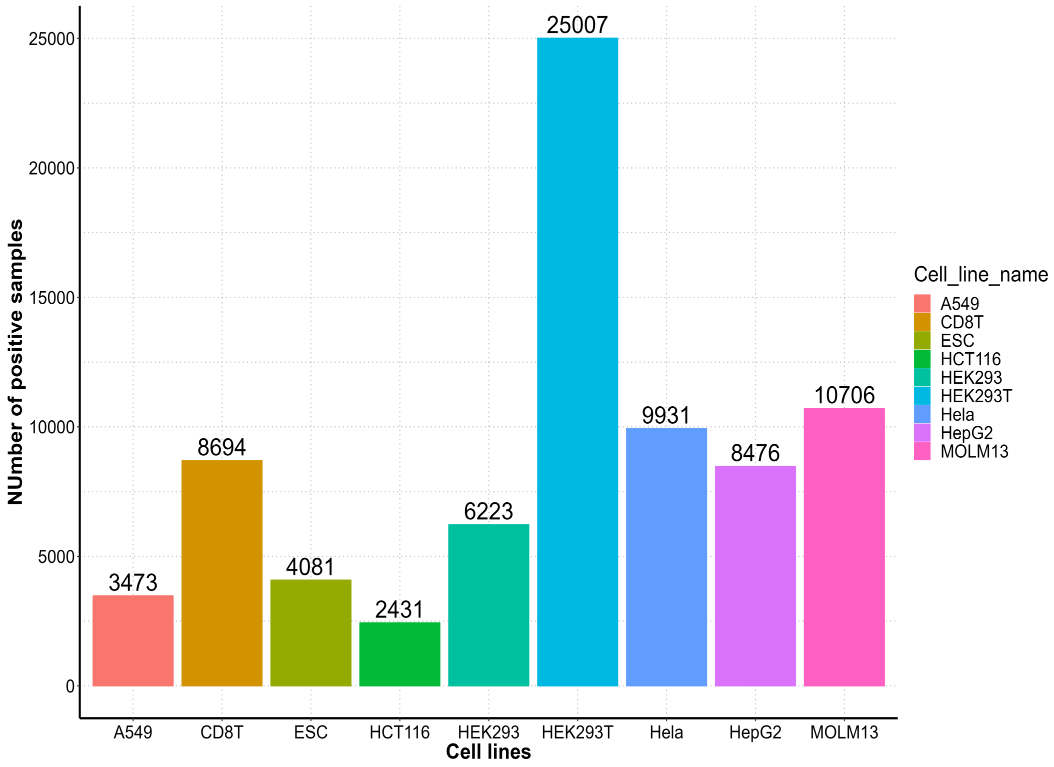

Supplementary Fig. 15: The distribution of human epitranscriptomic modifications across different nine cell lines.

Supplementary Figure 16

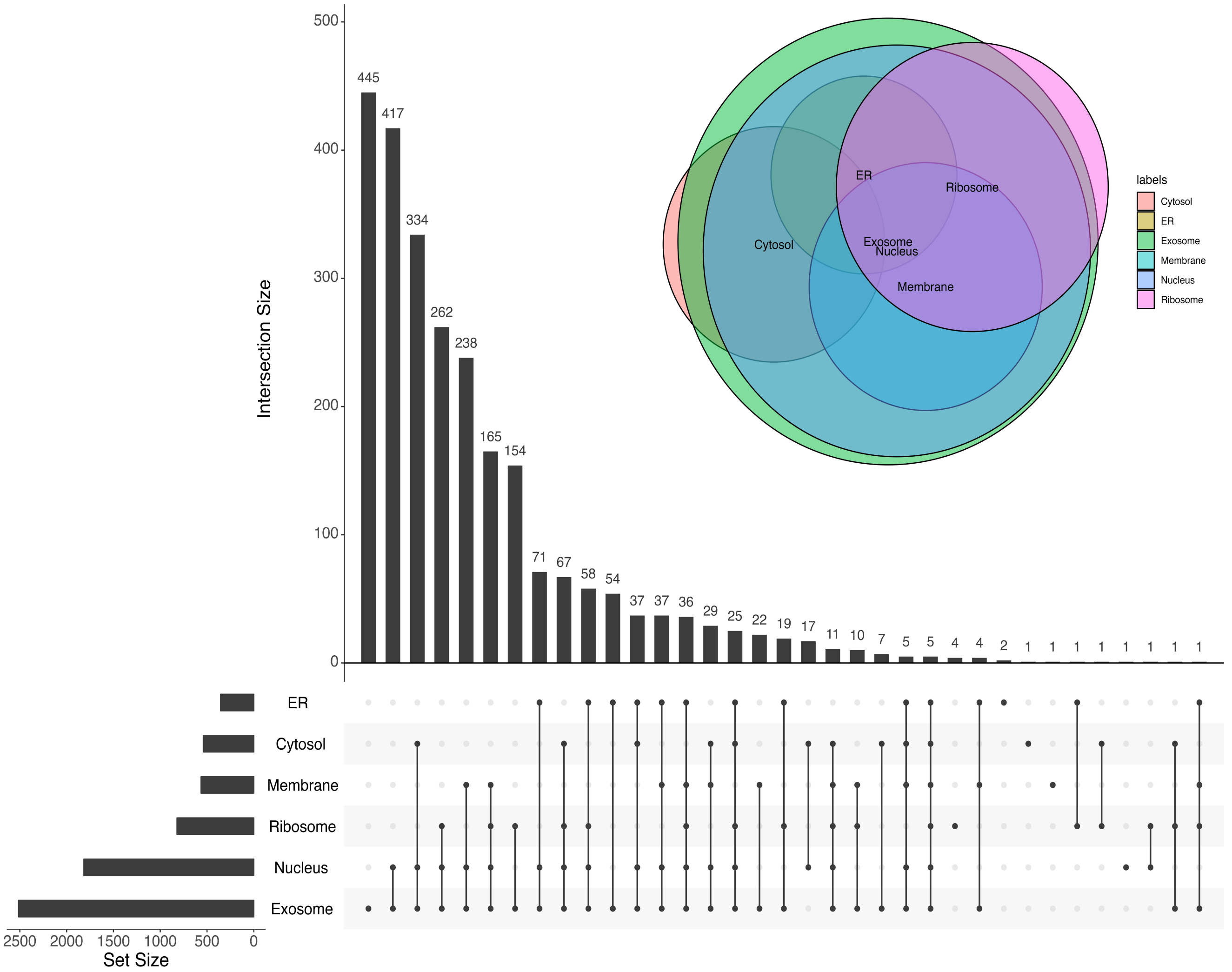

Supplementary Fig. 16: The venn upset showed the detailed number of mRNA sequences in each intersection group on independent test set. Upper bar plot presented the number of mRNAs in each intersection group, while the left bar plot indicated the total number of sequences for each subcompartment. And the bottom dots displayed the components of each group.

Supplementary Table. 1: Performance evaluation in terms of average ACCs, AUCs, AUPRCs, F1-score and MCCs with Std for 3UTRBERT, RmLR, BERT_RBP, DNABERT, RNABERT, RNAMSM, RNAFM, GraphProt2, RPI_Net, DeepCLIP and iDeepE on 22 eCLIP protocols.

Dataset_22_ACCs	3UTRBERT	RmLR	BERT_RBP	DNABERT	RNABERT	RNAMSM	RNAFM	GraphProt2	RPI_Net	DeepCLIP	iDeepE
AKAP1_HepG2	**0.822**±0.004	0.819±0.002	0.798±0.002	0.677±0.007	0.780±0.003	0.714±0.017	0.676±0.002	0.789±0.007	0.798±0.005	0.722±0.003	0.790±0.005
BCLAF1_HepG2	**0.812**±0.002	0.803±0.008	0.759±0.005	0.733±0.004	0.750±0.003	0.768±0.048	0.712±0.003	0.733±0.002	0.772±0.003	0.689±0.003	0.770±0.002
DDX3X_HepG2	0.877±0.003	**0.878**±0.003	0.874±0.003	0.863±0.003	0.873±0.003	0.857±0.009	0.859±0.002	0.869±0.007	0.870±0.001	0.860±0.005	0.870±0.002
DDX3X_K562	**0.856**±0.003	0.727±0.002	0.849±0.002	0.792±0.001	0.845±0.003	0.809±0.012	0.789±0.002	0.839±0.008	0.834±0.005	0.794±0.011	0.843±0.002
DDX24_K562	0.746±0.008	0.754±0.014	0.718±0.007	0.762±0.004	0.694±0.006	**0.807**±0.007	0.686±0.004	0.704±0.002	0.694±0.005	0.671±0.001	0.700±0.007
FAM120A_K562	0.789±0.006	**0.796**±0.004	0.764±0.004	0.687±0.003	0.746±0.020	0.693±0.019	0.680±0.005	0.757±0.009	0.767±0.003	0.700±0.006	0.778±0.010
G3BP1_HepG2	0.735±0.005	0.716±0.027	0.706±0.002	**0.748**±0.003	0.681±0.005	0.667±0.028	0.675±0.000	0.685±0.011	0.692±0.009	0.669±0.002	0.701±0.003
GRWD1_HepG2	**0.748**±0.004	0.724±0.039	0.712±0.005	0.676±0.004	0.698±0.001	0.704±0.015	0.678±0.006	0.694±0.003	0.703±0.003	0.671±0.004	0.710±0.003
IGF2BP1_K562	**0.791**±0.004	0.781±0.006	0.745±0.007	0.725±0.003	0.727±0.006	0.754±0.008	0.712±0.005	0.735±0.010	0.741±0.003	0.704±0.006	0.766±0.006
LARP4_HepG2	0.731±0.005	0.735±0.006	0.720±0.003	**0.748**±0.006	0.712±0.004	0.723±0.024	0.681±0.013	0.719±0.006	0.730±0.003	0.697±0.004	0.722±0.004
LIN28B_K562	0.694±0.004	0.686±0.005	0.675±0.004	**0.695**±0.008	0.675±0.005	0.636±0.063	0.686±0.005	0.680±0.003	0.685±0.004	0.682±0.003	0.675±0.005
PABPC4_K562	**0.753**±0.003	0.717±0.006	0.733±0.004	0.687±0.004	0.721±0.003	0.653±0.015	0.685±0.005	0.728±0.010	0.729±0.003	0.701±0.003	0.731±0.004
PPIG_HepG2	**0.810**±0.001	0.802±0.001	0.767±0.003	0.679±0.001	0.732±0.002	0.668±0.009	0.682±0.007	0.729±0.004	0.755±0.014	0.678±0.002	0.745±0.004
PUM2_K562	**0.913**±0.002	0.910±0.002	0.856±0.006	0.786±0.001	0.844±0.005	0.855±0.013	0.785±0.002	0.878±0.008	0.893±0.007	0.790±0.006	0.872±0.002
RBM15_K562	0.771±0.002	**0.774**±0.004	0.740±0.001	0.711±0.003	0.723±0.006	0.634±0.027	0.708±0.001	0.707±0.009	0.752±0.005	0.686±0.003	0.755±0.001
RPS3_HepG2	**0.758**±0.005	0.748±0.007	0.743±0.002	0.724±0.004	0.726±0.006	0.737±0.015	0.718±0.002	0.708±0.008	0.747±0.004	0.711±0.004	0.736±0.003
SND1_HepG2	**0.760**±0.005	0.753±0.014	0.734±0.003	0.694±0.003	0.712±0.003	0.710±0.012	0.695±0.001	0.703±0.007	0.728±0.008	0.682±0.004	0.736±0.005
UCHL5_K562	0.779±0.002	**0.789**±0.005	0.737±0.005	0.689±0.007	0.699±0.002	0.664±0.016	0.681±0.013	0.724±0.006	0.736±0.004	0.695±0.003	0.740±0.003
UPF1_HepG2	0.820±0.002	**0.822**±0.004	0.806±0.003	0.700±0.004	0.804±0.003	0.762±0.021	0.699±0.004	0.787±0.003	0.810±0.004	0.740±0.006	0.806±0.003
UPF1_K562	0.789±0.003	**0.795**±0.003	0.762±0.002	0.669±0.007	0.758±0.000	0.702±0.010	0.699±0.003	0.749±0.006	0.757±0.010	0.708±0.006	0.767±0.001
YBX3_K562	0.740±0.004	0.715±0.019	0.726±0.002	**0.753**±0.002	0.709±0.002	0.714±0.014	0.704±0.002	0.711±0.008	0.721±0.007	0.709±0.011	0.720±0.001
ZNF622_K562	**0.771**±0.004	0.762±0.006	0.730±0.006	0.689±0.003	0.706±0.005	0.661±0.009	0.677±0.006	0.726±0.009	0.738±0.002	0.690±0.004	0.736±0.003
Avg.±Std.	**0.785**±0.004	0.773±0.009	0.757±0.004	0.722±0.004	0.742±0.004	0.722±0.019	0.708±0.004	0.743±0.007	0.757±0.005	0.711±0.004	0.758±0.004

Dataset_22_AUCs	3UTRBERT	RmLR	BERT_RBP	DNABERT	RNABERT	RNAMSM	RNAFM	GraphProt2	RPI_Net	DeepCLIP	iDeepE
AKAP1_HepG2	**0.887**±0.004	0.886±0.002	0.860±0.001	0.702±0.004	0.841±0.001	0.802±0.012	0.693±0.005	0.844±0.007	0.767±0.006	0.749±0.005	0.744±0.008
BCLAF1_HepG2	**0.877**±0.002	0.872±0.008	0.806±0.003	0.787±0.004	0.805±0.004	0.747±0.062	0.766±0.002	0.787±0.002	0.722±0.009	0.741±0.001	0.719±0.006
DDX3X_HepG2	**0.938**±0.002	0.837±0.001	0.930±0.002	0.926±0.001	0.929±0.001	0.915±0.006	0.925±0.001	0.923±0.010	0.851±0.004	0.919±0.004	0.849±0.005
DDX3X_K562	**0.894**±0.005	0.805±0.004	0.886±0.001	0.828±0.002	0.877±0.003	0.855±0.011	0.828±0.001	0.876±0.004	0.799±0.009	0.829±0.011	0.805±0.004
DDX24_K562	0.798±0.005	0.790±0.020	0.734±0.007	**0.812**±0.005	0.676±0.016	0.640±0.004	0.714±0.009	0.703±0.006	0.611±0.014	0.593±0.008	0.624±0.010
FAM120A_K562	0.861±0.002	**0.866**±0.004	0.825±0.005	0.692±0.003	0.802±0.028	0.765±0.005	0.679±0.007	0.820±0.010	0.724±0.010	0.725±0.009	0.741±0.009
G3BP1_HepG2	0.786±0.006	0.719±0.098	0.724±0.008	**0.804**±0.003	0.653±0.044	0.609±0.006	0.526±0.038	0.668±0.010	0.611±0.015	0.558±0.006	0.606±0.005
GRWD1_HepG2	**0.806**±0.003	0.732±0.091	0.720±0.006	0.575±0.032	0.698±0.003	0.651±0.008	0.643±0.025	0.688±0.005	0.628±0.014	0.597±0.009	0.626±0.008
IGF2BP1_K562	**0.853**±0.003	0.846±0.005	0.806±0.003	0.778±0.002	0.788±0.004	0.729±0.002	0.765±0.003	0.784±0.012	0.697±0.017	0.747±0.008	0.725±0.005
LARP4_HepG2	0.769±0.004	0.761±0.006	0.728±0.006	**0.771**±0.004	0.708±0.008	0.657±0.010	0.594±0.059	0.710±0.012	0.625±0.009	0.652±0.010	0.626±0.004
LIN28B_K562	0.706±0.004	0.637±0.002	0.625±0.019	**0.714**±0.006	0.696±0.004	0.565±0.008	0.517±0.002	0.610±0.011	0.539±0.023	0.561±0.007	0.558±0.004
PABPC4_K562	**0.786**±0.003	0.786±0.003	0.730±0.008	0.528±0.008	0.713±0.003	0.680±0.012	0.512±0.001	0.725±0.012	0.633±0.010	0.658±0.011	0.634±0.010
PPIG_HepG2	**0.876**±0.003	0.870±0.001	0.821±0.005	0.710±0.001	0.780±0.005	0.746±0.010	0.713±0.007	0.760±0.003	0.692±0.032	0.689±0.008	0.685±0.007
PUM2_K562	0.962±0.001	**0.964**±0.002	0.924±0.002	0.828±0.000	0.916±0.002	0.926±0.007	0.827±0.000	0.941±0.004	0.877±0.011	0.837±0.008	0.855±0.007
RBM15_K562	**0.824**±0.002	0.822±0.004	0.771±0.004	0.727±0.002	0.745±0.007	0.681±0.018	0.724±0.002	0.721±0.007	0.692±0.013	0.684±0.008	0.687±0.004
RPS3_HepG2	**0.824**±0.004	0.817±0.005	0.804±0.003	0.781±0.002	0.784±0.003	0.706±0.021	0.776±0.002	0.752±0.008	0.698±0.012	0.744±0.011	0.677±0.014
SND1_HepG2	**0.815**±0.002	0.804±0.026	0.764±0.005	0.714±0.002	0.734±0.004	0.660±0.008	0.708±0.009	0.714±0.008	0.654±0.009	0.635±0.003	0.652±0.014
UCHL5_K562	0.822±0.004	**0.835**±0.002	0.749±0.005	0.645±0.023	0.736±0.001	0.702±0.007	0.594±0.059	0.726±0.004	0.657±0.009	0.660±0.003	0.659±0.003
UPF1_HepG2	**0.887**±0.002	0.887±0.003	0.865±0.002	0.740±0.001	0.860±0.002	0.832±0.016	0.736±0.002	0.843±0.006	0.772±0.008	0.773±0.006	0.770±0.003
UPF1_K562	0.857±0.003	**0.860**±0.001	0.820±0.002	0.581±0.038	0.810±0.002	0.763±0.008	0.739±0.002	0.799±0.009	0.723±0.015	0.725±0.006	0.722±0.005
YBX3_K562	0.753±0.004	0.586±0.135	0.702±0.004	**0.766**±0.001	0.668±0.006	0.638±0.005	0.533±0.001	0.651±0.010	0.589±0.023	0.559±0.007	0.591±0.004
ZNF622_K562	**0.819**±0.006	0.807±0.011	0.738±0.005	0.669±0.009	0.713±0.007	0.701±0.003	0.641±0.014	0.731±0.005	0.653±0.006	0.650±0.006	0.662±0.005
Avg.±Std.	**0.836**±0.003	0.809±0.020	0.788±0.005	0.731±0.007	0.770±0.007	0.726±0.011	0.689±0.011	0.762±0.007	0.692±0.013	0.695±0.007	0.692±0.007

Dataset_22_AUPRCs	3UTRBERT	RmLR	BERT_RBP	DNABERT	RNABERT	RNAMSM	RNAFM	GraphProt2	RPI_Net	DeepCLIP	iDeepE
AKAP1_HepG2	**0.782**±0.005	0.646±0.003	0.509±0.020	0.460±0.004	0.532±0.019	0.485±0.003	0.449±0.005	0.582±0.038	0.521±0.018	0.497±0.022	0.560±0.017
BCLAF1_HepG2	**0.787**±0.002	0.594±0.016	0.668±0.012	0.619±0.006	0.592±0.020	0.673±0.008	0.559±0.004	0.576±0.025	0.575±0.021	0.503±0.020	0.564±0.014
DDX3X_HepG2	0.656±0.004	0.529±0.003	0.533±0.013	0.856±0.002	0.521±0.006	**0.857**±0.001	0.853±0.003	0.527±0.014	0.532±0.017	0.506±0.014	0.524±0.013
DDX3X_K562	**0.868**±0.004	0.590±0.011	0.566±0.011	0.722±0.002	0.556±0.014	0.726±0.002	0.720±0.001	0.562±0.026	0.543±0.012	0.508±0.022	0.567±0.009
DDX24_K562	**0.837**±0.012	0.716±0.036	0.602±0.022	0.711±0.006	0.676±0.029	0.774±0.011	0.485±0.033	0.627±0.017	0.597±0.028	0.504±0.020	0.574±0.016
FAM120A_K562	**0.776**±0.007	0.654±0.003	0.587±0.034	0.540±0.004	0.571±0.046	0.569±0.005	0.517±0.012	0.556±0.023	0.564±0.035	0.503±0.015	0.538±0.020
G3BP1_HepG2	0.552±0.006	**0.702**±0.106	0.577±0.019	0.663±0.006	0.687±0.080	0.644±0.010	0.318±0.020	0.620±0.028	0.597±0.043	0.509±0.015	0.616±0.014
GRWD1_HepG2	0.576±0.005	**0.752**±0.124	0.608±0.018	0.370±0.031	0.647±0.030	0.421±0.023	0.471±0.026	0.647±0.021	0.574±0.041	0.497±0.042	0.598±0.018
IGF2BP1_K562	**0.719**±0.007	0.605±0.008	0.625±0.029	0.594±0.001	0.620±0.035	0.601±0.003	0.566±0.007	0.573±0.028	0.557±0.041	0.509±0.017	0.539±0.030
LARP4_HepG2	0.600±0.005	**0.745**±0.006	0.669±0.018	0.619±0.006	0.663±0.022	0.693±0.005	0.426±0.080	0.655±0.036	0.652±0.030	0.501±0.024	0.635±0.015
LIN28B_K562	0.383±0.007	0.669±0.005	0.692±0.079	0.542±0.007	0.524±0.026	0.556±0.002	0.383±0.005	0.663±0.028	**0.718**±0.056	0.510±0.087	0.633±0.017
PABPC4_K562	0.669±0.005	**0.808**±0.005	0.650±0.011	0.391±0.009	0.648±0.037	0.483±0.005	0.377±0.001	0.633±0.033	0.617±0.026	0.510±0.021	0.646±0.016
PPIG_HepG2	**0.780**±0.003	0.576±0.004	0.544±0.014	0.478±0.001	0.572±0.029	0.487±0.007	0.494±0.024	0.585±0.035	0.613±0.053	0.502±0.018	0.577±0.019
PUM2_K562	**0.934**±0.002	0.663±0.005	0.520±0.019	0.720±0.000	0.474±0.017	0.721±0.001	0.719±0.001	0.530±0.017	0.527±0.021	0.495±0.023	0.521±0.023
RBM15_K562	**0.688**±0.003	0.618±0.004	0.620±0.033	0.539±0.004	0.639±0.028	0.543±0.002	0.535±0.002	0.623±0.032	0.572±0.035	0.509±0.028	0.592±0.011
RPS3_HepG2	0.644±0.004	**0.667**±0.009	0.533±0.017	0.607±0.003	0.564±0.025	0.609±0.003	0.596±0.004	0.601±0.051	0.548±0.031	0.502±0.016	0.568±0.035
SND1_HepG2	0.625±0.008	**0.689**±0.039	0.588±0.064	0.443±0.008	0.606±0.046	0.536±0.007	0.436±0.007	0.622±0.044	0.557±0.032	0.504±0.018	0.592±0.028
UCHL5_K562	**0.747**±0.004	0.647±0.004	0.666±0.029	0.490±0.024	0.535±0.041	0.526±0.000	0.426±0.080	0.644±0.025	0.615±0.022	0.510±0.014	0.624±0.010
UPF1_HepG2	**0.783**±0.003	0.699±0.006	0.527±0.012	0.522±0.001	0.534±0.020	0.523±0.003	0.518±0.003	0.591±0.019	0.546±0.028	0.501±0.030	0.544±0.012
UPF1_K562	0.747±0.004	**0.833**±0.003	0.539±0.006	0.419±0.036	0.558±0.014	0.453±0.006	0.523±0.003	0.579±0.024	0.545±0.072	0.500±0.145	0.553±0.010
YBX3_K562	0.390±0.006	0.694±0.133	0.685±0.031	0.608±0.002	**0.698**±0.039	0.632±0.009	0.301±0.010	0.651±0.040	0.631±0.057	0.516±0.036	0.639±0.011
ZNF622_K562	**0.742**±0.004	0.671±0.018	0.660±0.019	0.502±0.009	0.676±0.024	0.647±0.006	0.472±0.017	0.619±0.042	0.628±0.020	0.506±0.031	0.615±0.018
Avg.±Std.	**0.695**±0.005	0.671±0.025	0.599±0.024	0.564±0.008	0.595±0.029	0.598±0.006	0.507±0.016	0.603±0.029	0.583±0.033	0.505±0.031	0.583±0.017

Dataset_22_F1s	3UTRBERT	RmLR	BERT_RBP	DNABERT	RNABERT	RNAMSM	RNAFM	GraphProt2	RPI_Net	DeepCLIP	iDeepE
AKAP1_HepG2	**0.799**±0.005	0.722±0.009	0.691±0.008	0.434±0.073	0.652±0.009	0.714±0.017	0.458±0.034	0.660±0.023	0.691±0.007	0.450±0.022	0.655±0.012
BCLAF1_HepG2	**0.785**±0.002	0.698±0.014	0.537±0.013	0.553±0.009	0.565±0.011	0.668±0.048	0.504±0.017	0.550±0.017	0.626±0.015	0.445±0.026	0.619±0.009
DDX3X_HepG2	**0.862**±0.004	0.814±0.008	0.806±0.006	0.785±0.006	0.804±0.005	0.857±0.009	0.776±0.009	0.797±0.010	0.803±0.003	0.783±0.011	0.802±0.005
DDX3X_K562	**0.834**±0.003	0.775±0.007	0.757±0.003	0.657±0.010	0.753±0.007	0.809±0.012	0.638±0.018	0.728±0.019	0.735±0.012	0.643±0.018	0.745±0.005
DDX24_K562	**0.708**±0.012	0.579±0.053	0.488±0.018	0.628±0.003	0.317±0.040	0.607±0.007	0.674±0.054	0.432±0.016	0.438±0.031	0.100±0.028	0.469±0.017
FAM120A_K562	**0.765**±0.007	0.701±0.004	0.619±0.022	0.351±0.053	0.597±0.050	0.693±0.019	0.309±0.070	0.598±0.015	0.632±0.017	0.401±0.016	0.658±0.012
G3BP1_HepG2	**0.692**±0.004	0.431±0.186	0.486±0.016	0.601±0.011	0.240±0.140	0.567±0.028	0.605±0.030	0.373±0.018	0.443±0.037	0.078±0.024	0.422±0.013
GRWD1_HepG2	**0.717**±0.004	0.441±0.257	0.452±0.013	0.400±0.001	0.379±0.040	0.604±0.015	0.369±0.069	0.375±0.023	0.473±0.034	0.072±0.039	0.463±0.018
IGF2BP1_K562	**0.760**±0.007	0.650±0.017	0.539±0.032	0.533±0.014	0.510±0.031	0.654±0.008	0.470±0.060	0.546±0.027	0.590±0.030	0.451±0.012	0.632±0.008
LARP4_HepG2	**0.691**±0.004	0.528±0.018	0.412±0.021	0.561±0.021	0.398±0.034	0.623±0.024	0.468±0.161	0.396±0.028	0.444±0.024	0.183±0.032	0.451±0.008
LIN28B_K562	**0.625**±0.008	0.191±0.084	0.206±0.100	0.495±0.010	0.476±0.020	0.536±0.063	0.491±0.084	0.200±0.040	0.198±0.111	0.026±0.016	0.311±0.015
PABPC4_K562	**0.708**±0.004	0.571±0.016	0.461±0.014	0.408±0.064	0.442±0.035	0.653±0.015	0.378±0.075	0.449±0.027	0.465±0.022	0.165±0.021	0.463±0.022
PPIG_HepG2	**0.784**±0.003	0.698±0.006	0.625±0.007	0.453±0.024	0.550±0.019	0.668±0.009	0.450±0.045	0.514±0.023	0.572±0.060	0.290±0.058	0.568±0.013
PUM2_K562	**0.905**±0.002	0.872±0.002	0.787±0.013	0.656±0.010	0.780±0.004	0.855±0.013	0.638±0.018	0.812±0.013	0.840±0.012	0.659±0.013	0.810±0.006
RBM15_K562	**0.731**±0.003	0.620±0.013	0.522±0.028	0.405±0.027	0.460±0.037	0.634±0.027	0.380±0.021	0.440±0.035	0.578±0.024	0.330±0.031	0.567±0.008
RPS3_HepG2	**0.724**±0.003	0.609±0.022	0.590±0.010	0.524±0.023	0.534±0.018	0.637±0.015	0.530±0.018	0.462±0.051	0.587±0.021	0.447±0.034	0.555±0.026
SND1_HepG2	**0.715**±0.008	0.564±0.083	0.505±0.058	0.442±0.194	0.425±0.049	0.610±0.012	0.361±0.136	0.355±0.044	0.512±0.019	0.087±0.027	0.501±0.027
UCHL5_K562	**0.744**±0.003	0.665±0.006	0.492±0.024	0.545±0.078	0.512±0.040	0.664±0.016	0.468±0.161	0.475±0.021	0.515±0.020	0.333±0.023	0.516±0.006
UPF1_HepG2	**0.798**±0.003	0.728±0.009	0.700±0.005	0.489±0.033	0.695±0.011	0.762±0.021	0.496±0.025	0.636±0.011	0.695±0.009	0.503±0.017	0.695±0.004
UPF1_K562	**0.765**±0.003	0.692±0.005	0.626±0.004	0.607±0.115	0.607±0.008	0.703±0.010	0.572±0.009	0.585±0.018	0.630±0.025	0.388±0.037	0.627±0.008
YBX3_K562	**0.665**±0.005	0.144±0.224	0.348±0.044	0.535±0.006	0.206±0.064	0.614±0.014	0.472±0.050	0.222±0.043	0.354±0.069	0.001±0.001	0.367±0.012
ZNF622_K562	**0.737**±0.003	0.654±0.018	0.478±0.027	0.430±0.055	0.407±0.037	0.661±0.009	0.587±0.105	0.473±0.023	0.504±0.014	0.290±0.036	0.526±0.012
Avg.±Std.	**0.750**±0.004	0.607±0.048	0.551±0.022	0.522±0.038	0.514±0.032	0.672±0.019	0.504±0.058	0.503±0.025	0.560±0.028	0.324±0.025	0.565±0.012

Dataset_22_MCCs	3UTRBERT	RmLR	BERT_RBP	DNABERT	RNABERT	RNAMSM	RNAFM	GraphProt2	RPI_Net	DeepCLIP	iDeepE
AKAP1_HepG2	**0.599**±0.010	0.588±0.008	0.541±0.006	0.425±0.037	0.493±0.009	0.529±0.240	0.233±0.020	0.512±0.019	0.542±0.010	0.315±0.012	0.508±0.014
BCLAF1_HepG2	**0.571**±0.004	0.552±0.019	0.418±0.013	0.371±0.006	0.404±0.005	0.500±0.005	0.314±0.011	0.373±0.007	0.472±0.011	0.250±0.017	0.461±0.007
DDX3X_HepG2	**0.724**±0.008	0.723±0.008	0.713±0.006	0.687±0.006	0.711±0.008	0.605±0.283	0.677±0.006	0.701±0.015	0.707±0.002	0.681±0.013	0.706±0.006
DDX3X_K562	0.670±0.006	**0.672**±0.005	0.652±0.005	0.516±0.003	0.644±0.008	0.551±0.118	0.505±0.008	0.619±0.021	0.616±0.013	0.508±0.026	0.636±0.005
DDX24_K562	0.417±0.021	0.414±0.046	0.311±0.019	0.454±0.006	0.201±0.025	0.715±0.011	0.258±0.039	0.269±0.006	0.255±0.021	**0.773**±0.013	0.272±0.020
FAM120A_K562	0.531±0.013	**0.548**±0.005	0.459±0.012	0.429±0.017	0.418±0.054	0.507±0.018	0.203±0.025	0.430±0.020	0.467±0.008	0.255±0.018	0.498±0.021
G3BP1_HepG2	0.385±0.007	0.284±0.130	0.290±0.006	**0.417**±0.009	0.144±0.061	0.407±0.121	0.411±0.037	0.207±0.028	0.251±0.024	0.148±0.009	0.252±0.008
GRWD1_HepG2	**0.434**±0.008	0.297±0.185	0.285±0.013	0.402±0.004	0.229±0.014	0.330±0.011	0.365±0.030	0.227±0.011	0.286±0.013	0.257±0.023	0.286±0.012
IGF2BP1_K562	**0.522**±0.013	0.495±0.017	0.390±0.022	0.350±0.010	0.347±0.019	0.474±0.029	0.304±0.028	0.369±0.028	0.405±0.018	0.275±0.013	0.464±0.006
LARP4_HepG2	0.382±0.008	0.358±0.006	0.287±0.013	**0.394**±0.020	0.265±0.018	0.235±0.133	0.316±0.108	0.263±0.015	0.311±0.008	0.143±0.022	0.303±0.009
LIN28B_K562	0.259±0.014	0.181±0.074	0.116±0.029	0.380±0.016	0.243±0.009	0.343±0.046	**0.381**±0.074	0.102±0.019	0.132±0.046	0.128±0.023	0.154±0.010
PABPC4_K562	**0.418**±0.006	0.412±0.011	0.327±0.013	0.310±0.057	0.295±0.013	0.340±0.022	0.274±0.069	0.306±0.026	0.316±0.011	0.168±0.016	0.323±0.016
PPIG_HepG2	**0.568**±0.006	0.550±0.004	0.459±0.007	0.234±0.015	0.368±0.006	0.374±0.017	0.239±0.027	0.344±0.006	0.415±0.047	0.158±0.025	0.396±0.010
PUM2_K562	**0.811**±0.004	0.803±0.003	0.679±0.016	0.508±0.006	0.661±0.007	0.351±0.196	0.500±0.009	0.722±0.018	0.760±0.016	0.513±0.015	0.715±0.005
RBM15_K562	0.464±0.005	**0.465**±0.010	0.367±0.011	0.267±0.011	0.310±0.025	0.356±0.085	0.255±0.005	0.273±0.030	0.412±0.015	0.191±0.011	0.410±0.005
RPS3_HepG2	**0.449**±0.006	0.424±0.022	0.405±0.006	0.340±0.016	0.349±0.013	0.345±0.040	0.335±0.009	0.280±0.030	0.408±0.014	0.278±0.019	0.375±0.015
SND1_HepG2	0.430±0.015	0.400±0.066	0.338±0.027	0.368±0.107	0.258±0.018	0.398±0.016	**0.432**±0.072	0.213±0.015	0.333±0.011	0.333±0.008	0.334±0.020
UCHL5_K562	0.490±0.005	**0.514**±0.010	0.359±0.013	0.387±0.035	0.301±0.027	0.337±0.308	0.316±0.108	0.322±0.017	0.361±0.013	0.218±0.011	0.370±0.008
UPF1_HepG2	**0.596**±0.005	0.596±0.010	0.557±0.006	0.388±0.023	0.552±0.010	0.477±0.006	0.290±0.019	0.497±0.008	0.561±0.008	0.365±0.016	0.555±0.006
UPF1_K562	0.530±0.006	**0.539**±0.004	0.455±0.004	0.355±0.079	0.438±0.004	0.307±0.118	0.328±0.009	0.415±0.016	0.457±0.006	0.278±0.020	0.462±0.005
YBX3_K562	0.340±0.008	0.105±0.157	0.243±0.018	0.375±0.004	0.147±0.022	**0.404**±0.008	0.260±0.024	0.147±0.006	0.230±0.028	0.304±0.006	0.236±0.003
ZNF622_K562	0.475±0.007	**0.498**±0.017	0.341±0.019	0.304±0.019	0.271±0.019	0.385±0.087	0.314±0.054	0.327±0.016	0.361±0.006	0.195±0.011	0.368±0.006
Avg.±Std.	**0.503**±0.008	0.473±0.037	0.409±0.013	0.394±0.023	0.366±0.018	0.421±0.087	0.341±0.036	0.360±0.017	0.412±0.016	0.306±0.016	0.413±0.010

Supplementary Table. 2: Performance evaluation in terms of average ACCs, AUCs, AUPRCs, F1-score and MCCs with Std for 3UTRBERT, RmLR, BERT_RBP, DNABERT, RNABERT, RNAMSM, RNAFM, GraphProt2, RPI_Net, DeepCLIP and iDeepE on 31 datasets covering CLIP/HITS-CLIP, iCLIP and PAR-CLIP from hg19.

Dataset_31_ACCs	3UTRBERT	RmLR	BERT_RBP	DNABERT	RNABERT	RNAMSM	RNAFM	GraphProt2	RPI_Net	DeepCLIP	iDeepE
AGO2	**0.856**±0.003	0.800±0.001	0.803±0.002	0.799±0.001	0.794±0.008	0.800±0.000	0.701±0.008	0.796±0.006	0.804±0.002	0.740±0.008	0.786±0.010
AGO2-M	**0.841**±0.006	0.799±0.002	0.802±0.003	0.798±0.005	0.792±0.006	0.741±0.004	0.703±0.005	0.791±0.005	0.786±0.016	0.733±0.006	0.737±0.006
AGO1234	**0.867**±0.006	0.802±0.009	0.795±0.003	0.798±0.007	0.822±0.006	0.782±0.000	0.742±0.004	0.794±0.007	0.801±0.002	0.736±0.009	0.720±0.004
Binding_1	**0.864**±0.005	0.862±0.007	0.859±0.006	0.858±0.007	0.821±0.003	0.822±0.007	0.820±0.005	0.844±0.004	0.841±0.005	0.745±0.011	0.835±0.003
Binding_2	**0.856**±0.004	0.854±0.011	0.844±0.005	0.843±0.007	0.810±0.003	0.809±0.004	0.808±0.003	0.840±0.007	0.837±0.006	0.746±0.006	0.830±0.007
eIF4AIII_1	0.932±0.003	**0.940**±0.004	0.918±0.004	0.915±0.008	0.804±0.004	0.807±0.003	0.807±0.007	0.897±0.006	0.891±0.005	0.801±0.003	0.905±0.007
eIF4AIII_2	0.930±0.004	**0.936**±0.003	0.911±0.003	0.912±0.003	0.841±0.005	0.841±0.003	0.845±0.002	0.897±0.007	0.897±0.005	0.818±0.006	0.888±0.001
ELVAL1-1	0.860±0.006	0.871±0.003	0.871±0.003	**0.872**±0.003	0.846±0.002	0.851±0.005	0.846±0.003	0.845±0.017	0.859±0.008	0.818±0.009	0.856±0.004
ELVAL1-2	0.898±0.004	0.901±0.005	**0.903**±0.005	0.895±0.007	0.866±0.003	0.869±0.004	0.863±0.002	0.865±0.002	0.892±0.005	0.820±0.010	0.890±0.007
ELVAL1-A	0.872±0.004	0.874±0.009	0.866±0.012	**0.880**±0.002	0.831±0.004	0.830±0.002	0.828±0.003	0.839±0.009	0.870±0.003	0.800±0.005	0.864±0.005
ELVAL1-M	0.787±0.009	0.784±0.005	0.803±0.003	0.799±0.005	0.821±0.013	0.809±0.004	**0.872**±0.012	0.798±0.003	0.804±0.004	0.748±0.010	0.733±0.006
EWSR1	0.871±0.008	0.868±0.006	0.879±0.012	**0.885**±0.004	0.842±0.010	0.852±0.010	0.840±0.004	0.810±0.007	0.873±0.005	0.773±0.007	0.870±0.008
FUS	0.902±0.005	**0.918**±0.005	0.895±0.008	0.908±0.005	0.860±0.006	0.860±0.007	0.861±0.007	0.814±0.008	0.900±0.007	0.780±0.009	0.891±0.003
hnRNPC-1	**0.922**±0.003	0.910±0.004	0.922±0.004	0.916±0.003	0.878±0.004	0.866±0.017	0.867±0.009	0.873±0.008	0.911±0.005	0.790±0.007	0.910±0.007
hnRNPC-2	**0.938**±0.006	0.915±0.005	0.935±0.003	0.934±0.005	0.917±0.004	0.915±0.009	0.913±0.009	0.892±0.009	0.929±0.003	0.801±0.009	0.933±0.003
hnRNPL-1	0.781±0.012	0.789±0.016	0.777±0.014	0.762±0.028	**0.821**±0.004	0.799±0.001	0.799±0.002	0.800±0.004	0.764±0.017	0.737±0.011	0.809±0.008
hnRNPL-2	0.779±0.011	0.788±0.022	0.773±0.018	0.771±0.017	**0.883**±0.006	0.793±0.005	0.791±0.010	0.795±0.005	0.792±0.010	0.745±0.023	0.733±0.003
HnRNPL-L	0.791±0.008	0.812±0.004	0.803±0.004	0.785±0.006	**0.813**±0.007	0.800±0.001	0.801±0.001	0.801±0.005	0.803±0.002	0.746±0.010	0.805±0.007
IGF2BP1-3	0.808±0.006	0.791±0.010	0.799±0.003	0.794±0.013	0.803±0.004	0.803±0.003	**0.833**±0.001	0.785±0.011	0.801±0.004	0.735±0.012	0.811±0.007
MOV10	0.808±0.006	0.810±0.003	0.817±0.005	0.809±0.007	**0.831**±0.005	0.802±0.002	0.790±0.004	0.806±0.014	0.820±0.007	0.741±0.017	0.801±0.006
mut-FUS	0.904±0.006	0.905±0.008	0.902±0.011	**0.913**±0.007	0.854±0.001	0.849±0.006	0.846±0.003	0.818±0.009	0.910±0.004	0.778±0.009	0.898±0.005
NSUN2	0.813±0.002	0.803±0.018	0.813±0.007	0.816±0.026	0.818±0.005	0.814±0.003	0.810±0.006	0.802±0.007	0.806±0.006	0.747±0.009	**0.836**±0.005
PUM2	**0.934**±0.004	0.927±0.005	0.931±0.003	0.927±0.007	0.854±0.002	0.854±0.002	0.851±0.005	0.888±0.010	0.889±0.006	0.809±0.015	0.910±0.002
QKI	0.935±0.004	0.931±0.001	**0.943**±0.011	0.936±0.005	0.894±0.001	0.888±0.003	0.891±0.004	0.912±0.007	0.940±0.004	0.817±0.013	0.934±0.007
SFRS1	**0.881**±0.004	0.873±0.007	0.867±0.004	0.858±0.010	0.816±0.003	0.805±0.004	0.805±0.003	0.851±0.011	0.840±0.010	0.799±0.002	0.853±0.008
TAF1S	0.915±0.006	**0.926**±0.006	0.886±0.016	0.925±0.004	0.845±0.011	0.848±0.015	0.833±0.015	0.826±0.003	0.916±0.005	0.754±0.011	0.914±0.008
TDP-43	0.899±0.003	0.884±0.004	0.900±0.003	**0.901**±0.002	0.847±0.005	0.850±0.009	0.850±0.004	0.890±0.004	0.899±0.006	0.765±0.008	0.895±0.006
TIA1	0.888±0.003	**0.890**±0.006	0.873±0.005	0.884±0.006	0.857±0.008	0.857±0.005	0.854±0.006	0.832±0.007	0.861±0.006	0.797±0.009	0.874±0.005
TIAL1	0.847±0.005	0.836±0.005	**0.860**±0.004	0.840±0.007	0.830±0.008	0.829±0.005	0.821±0.009	0.834±0.013	0.844±0.008	0.784±0.007	0.852±0.008
U2AF65	0.916±0.004	**0.919**±0.005	0.917±0.008	0.912±0.007	0.877±0.007	0.882±0.008	0.885±0.003	0.832±0.011	0.879±0.005	0.786±0.010	0.902±0.009
Y2AF65	**0.888**±0.003	0.870±0.005	0.878±0.007	0.873±0.005	0.842±0.008	0.846±0.008	0.847±0.006	0.841±0.009	0.864±0.009	0.789±0.007	0.866±0.001
Avg.±Std.	**0.871**±0.005	0.864±0.007	0.863±0.006	0.862±0.007	0.840±0.005	0.831±0.005	0.827±0.005	0.836±0.008	0.856±0.006	0.773±0.009	0.850±0.006

Dataset_31_AUCs	3UTRBERT	RmLR	BERT_RBP	DNABERT	RNABERT	RNAMSM	RNAFM	GraphProt2	RPI_Net	DeepCLIP	iDeepE
AGO2	0.684±0.012	0.639±0.005	**0.720**±0.014	0.617±0.021	0.649±0.006	0.624±0.003	0.626±0.001	0.712±0.022	0.633±0.008	0.579±0.021	0.513±0.012
AGO2-M	**0.671**±0.010	0.609±0.016	0.633±0.015	0.591±0.020	0.627±0.005	0.572±0.001	0.571±0.001	0.600±0.017	0.669±0.008	0.518±0.009	0.631±0.028
AGO1234	**0.813**±0.008	0.668±0.016	0.609±0.015	0.626±0.014	0.792±0.004	0.612±0.001	0.611±0.001	0.585±0.012	0.745±0.010	0.572±0.016	0.654±0.013
Binding_1	0.893±0.005	**0.899**±0.010	0.888±0.008	0.872±0.011	0.685±0.002	0.688±0.002	0.679±0.010	0.841±0.016	0.638±0.011	0.558±0.020	0.641±0.004
Binding_2	**0.893**±0.004	0.881±0.008	0.871±0.004	0.865±0.008	0.674±0.003	0.675±0.005	0.678±0.009	0.845±0.011	0.652±0.019	0.578±0.011	0.624±0.010
eIF4AIII_1	**0.965**±0.002	0.965±0.002	0.947±0.004	0.950±0.003	0.813±0.002	0.820±0.005	0.812±0.005	0.930±0.006	0.810±0.021	0.752±0.022	0.823±0.014
eIF4AIII_2	**0.964**±0.003	0.961±0.009	0.955±0.005	0.952±0.002	0.865±0.001	0.868±0.002	0.867±0.002	0.938±0.008	0.826±0.013	0.810±0.004	0.806±0.013
ELVAL1-1	0.910±0.004	0.913±0.006	**0.919**±0.004	0.919±0.005	0.898±0.001	0.900±0.001	0.897±0.001	0.883±0.018	0.775±0.014	0.841±0.008	0.763±0.020
ELVAL1-2	**0.925**±0.003	0.919±0.004	0.925±0.004	0.916±0.005	0.898±0.001	0.901±0.002	0.897±0.001	0.906±0.013	0.840±0.019	0.846±0.011	0.813±0.019
ELVAL1-A	0.892±0.004	0.886±0.004	**0.894**±0.006	0.892±0.007	0.836±0.001	0.835±0.002	0.833±0.001	0.850±0.007	0.789±0.017	0.804±0.009	0.761±0.018
ELVAL1-M	0.639±0.013	0.635±0.009	0.627±0.014	0.602±0.011	**0.852**±0.008	0.542±0.002	0.542±0.003	0.582±0.020	0.662±0.020	0.475±0.015	0.660±0.039
EWSR1	0.893±0.006	0.883±0.003	**0.900**±0.007	0.900±0.004	0.876±0.007	0.880±0.006	0.872±0.006	0.810±0.009	0.774±0.005	0.752±0.015	0.755±0.011
FUS	0.930±0.008	**0.941**±0.004	0.932±0.005	0.938±0.002	0.890±0.002	0.890±0.004	0.889±0.003	0.823±0.015	0.850±0.020	0.762±0.013	0.815±0.010
hnRNPC-1	0.959±0.002	0.945±0.004	**0.960**±0.002	0.954±0.002	0.931±0.002	0.930±0.002	0.931±0.002	0.903±0.011	0.857±0.012	0.778±0.009	0.840±0.020
hnRNPC-2	**0.978**±0.001	0.978±0.003	0.976±0.002	0.974±0.002	0.960±0.002	0.959±0.002	0.960±0.001	0.937±0.007	0.884±0.015	0.818±0.012	0.877±0.003
hnRNPL-1	0.695±0.014	0.669±0.018	0.654±0.013	0.677±0.042	**0.849**±0.002	0.719±0.050	0.736±0.038	0.663±0.016	0.663±0.011	0.602±0.012	0.596±0.014
hnRNPL-2	0.665±0.011	0.638±0.019	0.613±0.011	0.633±0.057	**0.931**±0.002	0.735±0.011	0.713±0.014	0.651±0.012	0.616±0.008	0.621±0.021	0.642±0.019
HnRNPL-L	0.675±0.009	0.627±0.012	0.658±0.008	0.652±0.024	**0.849**±0.005	0.685±0.013	0.668±0.028	0.647±0.016	0.509±0.006	0.603±0.023	0.578±0.017
IGF2BP1-3	**0.804**±0.003	0.733±0.008	0.707±0.019	0.709±0.024	0.746±0.005	0.652±0.001	0.654±0.002	0.676±0.031	0.737±0.005	0.620±0.010	0.777±0.006
MOV10	0.819±0.006	0.810±0.006	0.836±0.004	0.813±0.009	**0.849**±0.003	0.781±0.001	0.777±0.001	0.792±0.005	0.844±0.004	0.696±0.033	0.601±0.016
mut-FUS	0.937±0.003	**0.942**±0.004	0.940±0.002	0.942±0.003	0.872±0.006	0.875±0.003	0.873±0.003	0.846±0.012	0.890±0.013	0.762±0.018	0.821±0.015
NSUN2	0.823±0.006	**0.824**±0.023	0.807±0.009	0.818±0.038	0.781±0.008	0.775±0.004	0.767±0.008	0.762±0.010	0.593±0.028	0.626±0.020	0.691±0.011
PUM2	0.967±0.001	0.968±0.002	**0.974**±0.002	0.966±0.003	0.890±0.001	0.891±0.001	0.890±0.001	0.931±0.010	0.815±0.020	0.832±0.018	0.839±0.009
QKI	0.956±0.005	**0.958**±0.003	0.956±0.004	0.954±0.002	0.928±0.001	0.928±0.002	0.929±0.002	0.936±0.011	0.902±0.011	0.827±0.013	0.885±0.020
SFRS1	**0.910**±0.003	0.905±0.004	0.891±0.005	0.880±0.004	0.827±0.005	0.776±0.001	0.773±0.001	0.856±0.013	0.695±0.015	0.626±0.038	0.706±0.015
TAF1S	0.950±0.005	0.948±0.008	0.936±0.013	**0.954**±0.006	0.882±0.011	0.885±0.011	0.869±0.015	0.853±0.006	0.894±0.008	0.723±0.023	0.861±0.012
TDP-43	0.914±0.009	0.902±0.010	**0.918**±0.004	0.918±0.007	0.850±0.002	0.849±0.002	0.848±0.002	0.882±0.008	0.773±0.026	0.668±0.019	0.776±0.011
TIA1	**0.933**±0.003	0.932±0.003	0.918±0.004	0.922±0.005	0.896±0.005	0.892±0.003	0.886±0.003	0.870±0.012	0.770±0.009	0.793±0.013	0.789±0.020
TIAL1	**0.883**±0.003	0.876±0.003	0.878±0.006	0.865±0.005	0.869±0.008	0.868±0.001	0.864±0.007	0.836±0.011	0.752±0.011	0.771±0.008	0.731±0.008
U2AF65	0.958±0.004	**0.968**±0.003	0.960±0.005	0.957±0.004	0.924±0.001	0.928±0.002	0.926±0.004	0.857±0.009	0.807±0.005	0.782±0.010	0.825±0.023
Y2AF65	0.930±0.003	**0.931**±0.005	0.922±0.005	0.920±0.004	0.882±0.006	0.886±0.003	0.880±0.005	0.857±0.011	0.785±0.015	0.791±0.013	0.766±0.008
Avg.±Std.	**0.865**±0.006	0.850±0.008	0.849±0.007	0.844±0.011	0.841±0.004	0.801±0.005	0.797±0.006	0.808±0.012	0.756±0.013	0.703±0.016	0.737±0.015

Dataset_31_AUPRCs	3UTRBERT	RmLR	BERT_RBP	DNABERT	RNABERT	RNAMSM	RNAFM	GraphProt2	RPI_Net	DeepCLIP	iDeepE
AGO2	0.448±0.022	0.288±0.011	0.712±0.026	0.583±0.010	0.544±0.047	0.265±0.004	0.268±0.002	0.675±0.046	**0.733**±0.031	0.541±0.024	0.695±0.063
AGO2-M	0.288±0.015	0.265±0.024	**0.926**±0.094	0.528±0.019	0.556±0.034	0.244±0.002	0.245±0.002	0.686±0.082	0.808±0.037	0.529±0.021	0.656±0.087
AGO1234	0.282±0.014	0.383±0.012	0.669±0.105	0.619±0.021	0.548±0.042	0.246±0.001	0.245±0.001	0.647±0.113	**0.687**±0.061	0.538±0.016	0.570±0.042
Binding_1	0.709±0.012	0.678±0.031	0.608±0.027	0.710±0.021	0.575±0.032	0.439±0.003	0.429±0.008	0.622±0.018	**0.716**±0.033	0.516±0.025	0.682±0.015
Binding_2	0.665±0.009	0.649±0.023	0.624±0.027	0.706±0.017	0.565±0.036	0.426±0.006	0.427±0.003	0.636±0.049	0.646±0.062	0.505±0.020	**0.712**±0.032
eIF4AIII_1	0.734±0.007	**0.842**±0.010	0.520±0.023	0.608±0.017	0.556±0.054	0.491±0.009	0.484±0.011	0.588±0.043	0.565±0.042	0.516±0.056	0.597±0.033
eIF4AIII_2	0.804±0.007	**0.857**±0.006	0.544±0.022	0.558±0.005	0.548±0.044	0.613±0.004	0.609±0.006	0.596±0.039	0.537±0.021	0.496±0.068	0.553±0.026
ELVAL1-1	**0.752**±0.015	0.741±0.018	0.611±0.036	0.642±0.011	0.539±0.042	0.640±0.003	0.636±0.002	0.580±0.068	0.520±0.038	0.493±0.022	0.514±0.030
ELVAL1-2	0.352±0.007	**0.781**±0.005	0.590±0.021	0.598±0.013	0.546±0.027	0.692±0.005	0.683±0.004	0.629±0.030	0.487±0.042	0.491±0.026	0.535±0.029
ELVAL1-A	0.706±0.011	**0.756**±0.011	0.675±0.049	0.545±0.014	0.561±0.029	0.543±0.007	0.537±0.004	0.586±0.052	0.523±0.026	0.501±0.018	0.569±0.026
ELVAL1-M	**0.825**±0.012	0.285±0.016	0.753±0.080	0.527±0.016	0.552±0.078	0.224±0.003	0.224±0.002	0.687±0.093	0.742±0.049	0.511±0.018	0.598±0.094
EWSR1	0.455±0.014	**0.711**±0.006	0.625±0.052	0.540±0.010	0.563±0.047	0.626±0.014	0.613±0.016	0.589±0.046	0.558±0.016	0.509±0.031	0.591±0.023
FUS	0.377±0.008	**0.805**±0.014	0.560±0.028	0.735±0.010	0.550±0.066	0.653±0.019	0.646±0.012	0.612±0.046	0.525±0.043	0.540±0.026	0.542±0.024
hnRNPC-1	0.530±0.009	**0.822**±0.009	0.523±0.014	0.705±0.007	0.536±0.067	0.787±0.006	0.781±0.004	0.594±0.035	0.519±0.028	0.545±0.016	0.557±0.029
hnRNPC-2	0.564±0.011	**0.903**±0.006	0.525±0.014	0.490±0.020	0.550±0.021	0.880±0.006	0.882±0.005	0.599±0.051	0.526±0.029	0.518±0.024	0.544±0.014
hnRNPL-1	**0.897**±0.013	0.336±0.026	0.645±0.050	0.538±0.072	0.524±0.043	0.360±0.055	0.374±0.044	0.688±0.052	0.577±0.035	0.516±0.020	0.662±0.038
hnRNPL-2	**0.902**±0.031	0.294±0.047	0.572±0.048	0.465±0.092	0.550±0.041	0.387±0.020	0.352±0.023	0.699±0.060	0.639±0.091	0.506±0.026	0.597±0.045
HnRNPL-L	0.750±0.019	0.354±0.018	0.776±0.038	0.683±0.021	0.713±0.082	0.367±0.016	0.348±0.028	0.687±0.053	**0.858**±0.062	0.505±0.031	0.678±0.038
IGF2BP1-3	**0.881**±0.013	0.353±0.016	0.690±0.039	0.592±0.027	0.538±0.024	0.286±0.001	0.287±0.002	0.619±0.059	0.692±0.063	0.505±0.026	0.532±0.014
MOV10	**0.828**±0.021	0.482±0.018	0.714±0.056	0.563±0.022	0.564±0.034	0.382±0.002	0.379±0.002	0.604±0.067	0.657±0.044	0.511±0.053	0.622±0.036
mut-FUS	0.788±0.016	**0.798**±0.020	0.614±0.039	0.491±0.007	0.551±0.046	0.627±0.004	0.625±0.007	0.589±0.048	0.466±0.032	0.505±0.032	0.552±0.027
NSUN2	**0.678**±0.004	0.522±0.076	0.580±0.057	0.535±0.112	0.532±0.061	0.467±0.009	0.449±0.018	0.657±0.034	0.621±0.062	0.513±0.022	0.592±0.021
PUM2	**0.901**±0.007	0.889±0.006	0.536±0.016	0.694±0.007	0.543±0.064	0.650±0.003	0.645±0.002	0.574±0.038	0.550±0.035	0.526±0.036	0.562±0.019
QKI	0.807±0.008	**0.893**±0.008	0.515±0.032	0.529±0.014	0.545±0.032	0.788±0.010	0.792±0.008	0.558±0.033	0.523±0.022	0.523±0.038	0.561±0.033
SFRS1	**0.901**±0.008	0.685±0.009	0.578±0.023	0.692±0.009	0.727±0.014	0.457±0.006	0.451±0.004	0.640±0.046	0.588±0.049	0.526±0.200	0.617±0.040
TAF1S	**0.913**±0.012	0.853±0.008	0.636±0.070	0.671±0.008	0.766±0.070	0.619±0.032	0.572±0.035	0.640±0.028	0.472±0.025	0.557±0.023	0.519±0.024
TDP-43	**0.855**±0.011	0.818±0.006	0.586±0.029	0.603±0.008	0.550±0.071	0.649±0.002	0.647±0.002	0.637±0.048	0.692±0.058	0.529±0.034	0.656±0.025
TIA1	**0.923**±0.009	0.769±0.010	0.660±0.050	0.570±0.010	0.544±0.067	0.649±0.007	0.638±0.005	0.583±0.030	0.516±0.020	0.514±0.030	0.547±0.028
TIAL1	0.478±0.009	0.631±0.018	0.651±0.031	**0.671**±0.013	0.549±0.047	0.616±0.007	0.604±0.019	0.589±0.051	0.498±0.029	0.521±0.022	0.585±0.022
U2AF65	0.428±0.010	**0.845**±0.006	0.586±0.032	0.629±0.009	0.523±0.031	0.723±0.004	0.715±0.008	0.565±0.045	0.514±0.019	0.503±0.038	0.561±0.034
Y2AF65	**0.803**±0.009	0.724±0.004	0.597±0.047	0.602±0.016	0.543±0.049	0.605±0.008	0.598±0.013	0.578±0.028	0.495±0.029	0.503±0.033	0.552±0.017
Avg.±Std.	**0.685**±0.012	0.646±0.016	0.626±0.041	0.601±0.021	0.566±0.047	0.529±0.009	0.522±0.010	0.620±0.049	0.595±0.040	0.516±0.035	0.591±0.033

Dataset_31_F1s	3UTRBERT	RmLR	BERT_RBP	DNABERT	RNABERT	RNAMSM	RNAFM	GraphProt2	RPI_Net	DeepCLIP	iDeepE
AGO2	**0.573**±0.028	0.008±0.017	0.149±0.028	0.061±0.050	0.518±0.038	0.465±0.075	0.441±0.008	0.217±0.066	0.072±0.042	0.191±0.023	0.098±0.028
AGO2-M	**0.546**±0.007	0.022±0.026	0.053±0.019	0.028±0.018	0.524±0.033	0.528±0.052	0.543±0.005	0.064±0.056	0.106±0.022	0.098±0.020	0.444±0.071
AGO1234	0.651±0.012	0.343±0.032	0.056±0.065	0.180±0.021	**0.684**±0.020	0.570±0.004	0.582±0.004	0.045±0.031	0.099±0.020	0.165±0.013	0.518±0.029
Binding_1	**0.779**±0.011	0.631±0.024	0.594±0.029	0.585±0.025	0.342±0.040	0.310±0.077	0.295±0.081	0.524±0.019	0.430±0.025	0.144±0.021	0.435±0.010
Binding_2	**0.771**±0.007	0.608±0.036	0.528±0.021	0.557±0.030	0.274±0.023	0.279±0.012	0.286±0.003	0.520±0.048	0.453±0.031	0.171±0.020	0.398±0.023
eIF4AIII_1	**0.896**±0.005	0.850±0.010	0.792±0.013	0.798±0.015	0.344±0.064	0.407±0.062	0.391±0.058	0.722±0.022	0.710±0.021	0.396±0.043	0.743±0.019
eIF4AIII_2	**0.892**±0.005	0.845±0.007	0.770±0.010	0.787±0.008	0.535±0.039	0.541±0.028	0.561±0.027	0.722±0.028	0.732±0.017	0.263±0.101	0.705±0.011
ELVAL1-1	**0.782**±0.014	0.677±0.010	0.633±0.019	0.677±0.018	0.594±0.031	0.584±0.023	0.570±0.032	0.559±0.073	0.641±0.015	0.509±0.011	0.628±0.022
ELVAL1-2	**0.839**±0.005	0.747±0.017	0.735±0.017	0.735±0.017	0.652±0.013	0.640±0.051	0.630±0.041	0.608±0.016	0.734±0.015	0.518±0.024	0.714±0.024
ELVAL1-A	**0.795**±0.008	0.712±0.016	0.580±0.060	0.688±0.018	0.548±0.017	0.544±0.020	0.535±0.034	0.536±0.024	0.666±0.018	0.460±0.018	0.634±0.024
ELVAL1-M	**0.558**±0.016	0.168±0.064	0.102±0.044	0.442±0.044	0.331±0.147	0.482±0.012	0.472±0.012	0.055±0.030	0.132±0.037	0.089±0.019	0.509±0.087
EWSR1	**0.796**±0.013	0.676±0.023	0.656±0.051	0.700±0.006	0.484±0.071	0.562±0.048	0.462±0.051	0.408±0.043	0.656±0.010	0.356±0.033	0.634±0.021
FUS	**0.844**±0.008	0.796±0.018	0.722±0.025	0.768±0.019	0.622±0.042	0.599±0.030	0.600±0.031	0.403±0.049	0.752±0.018	0.387±0.025	0.717±0.009
hnRNPC-1	**0.877**±0.006	0.829±0.009	0.801±0.010	0.800±0.003	0.696±0.028	0.698±0.008	0.707±0.005	0.646±0.021	0.773±0.012	0.421±0.010	0.762±0.025
hnRNPC-2	**0.901**±0.010	0.841±0.015	0.834±0.008	0.837±0.012	0.789±0.007	0.789±0.011	0.784±0.007	0.710±0.034	0.818±0.011	0.459±0.021	0.823±0.007
hnRNPL-1	**0.599**±0.013	0.449±0.050	0.121±0.021	0.190±0.118	0.365±0.041	0.091±0.192	0.152±0.210	0.172±0.049	0.158±0.024	0.210±0.017	0.336±0.032
hnRNPL-2	0.547±0.030	0.389±0.037	0.135±0.021	0.100±0.103	**0.702**±0.014	0.236±0.137	0.164±0.156	0.092±0.042	0.061±0.020	0.240±0.011	0.480±0.041
HnRNPL-L	**0.592**±0.018	0.333±0.076	0.156±0.018	0.194±0.079	0.292±0.111	0.029±0.022	0.028±0.035	0.177±0.041	0.039±0.026	0.239±0.029	0.289±0.041
IGF2BP1-3	0.329±0.016	0.209±0.082	0.094±0.029	0.137±0.078	0.592±0.015	0.563±0.010	0.533±0.001	0.154±0.035	0.120±0.035	0.194±0.026	**0.702**±0.009
MOV10	**0.652**±0.027	0.515±0.031	0.324±0.070	0.379±0.045	0.501±0.025	0.610±0.072	0.105±0.072	0.391±0.028	0.414±0.039	0.248±0.050	0.349±0.036
mut-FUS	**0.851**±0.014	0.794±0.018	0.725±0.046	0.785±0.021	0.607±0.029	0.578±0.031	0.569±0.033	0.474±0.052	0.790±0.005	0.384±0.032	0.730±0.017
NSUN2	**0.677**±0.003	0.558±0.059	0.459±0.039	0.460±0.100	0.393±0.080	0.356±0.041	0.334±0.027	0.304±0.036	0.322±0.066	0.220±0.019	0.523±0.019
PUM2	**0.898**±0.006	0.820±0.009	0.821±0.009	0.814±0.018	0.554±0.048	0.554±0.034	0.554±0.063	0.705±0.026	0.711±0.022	0.485±0.032	0.762±0.008
QKI	**0.900**±0.006	0.857±0.003	0.857±0.026	0.845±0.012	0.701±0.015	0.678±0.017	0.681±0.026	0.770±0.015	0.847±0.011	0.501±0.036	0.829±0.021
SFRS1	**0.806**±0.005	0.678±0.016	0.629±0.010	0.608±0.022	0.430±0.010	0.069±0.064	0.077±0.054	0.542±0.035	0.530±0.022	0.108±0.010	0.556±0.026
TAF1S	**0.871**±0.011	0.827±0.010	0.669±0.071	0.815±0.014	0.580±0.062	0.583±0.055	0.497±0.081	0.449±0.035	0.801±0.006	0.296±0.020	0.782±0.018
TDP-43	**0.823**±0.008	0.736±0.004	0.725±0.008	0.725±0.005	0.497±0.051	0.512±0.030	0.507±0.020	0.680±0.018	0.688±0.034	0.301±0.034	0.688±0.018
TIA1	**0.817**±0.005	0.715±0.013	0.619±0.035	0.695±0.029	0.656±0.036	0.638±0.021	0.622±0.016	0.519±0.027	0.638±0.014	0.440±0.030	0.673±0.023
TIAL1	**0.752**±0.007	0.614±0.018	0.567±0.029	0.589±0.008	0.566±0.030	0.584±0.032	0.556±0.048	0.526±0.033	0.603±0.014	0.411±0.018	0.589±0.016
U2AF65	**0.869**±0.008	0.829±0.009	0.774±0.030	0.787±0.018	0.716±0.005	0.726±0.006	0.723±0.012	0.528±0.032	0.692±0.007	0.416±0.034	0.740±0.031
Y2AF65	**0.826**±0.006	0.740±0.013	0.656±0.034	0.691±0.019	0.622±0.034	0.635±0.022	0.621±0.035	0.537±0.020	0.656±0.022	0.424±0.034	0.641±0.008
Avg.±Std.	**0.752**±0.011	0.607±0.025	0.527±0.029	0.563±0.032	0.539±0.039	0.498±0.042	0.470±0.042	0.444±0.035	0.511±0.022	0.314±0.027	0.594±0.025

Dataset_31_MCCs	3UTRBERT	RmLR	BERT_RBP	DNABERT	RNABERT	RNAMSM	RNAFM	GraphProt2	RPI_Net	DeepCLIP	iDeepE
AGO2	**0.504**±0.043	0.012±0.037	0.155±0.013	0.169±0.053	0.121±0.033	0.117±0.029	0.107±0.026	0.168±0.046	0.121±0.035	0.049±0.027	0.058±0.052
AGO2-M	**0.461**±0.021	0.033±0.032	0.101±0.042	0.153±0.022	0.127±0.031	0.236±0.022	0.274±0.026	0.033±0.060	0.070±0.043	0.139±0.021	0.340±0.022
AGO1234	**0.572**±0.021	0.261±0.027	0.044±0.051	0.156±0.025	0.382±0.020	0.165±0.003	0.140±0.035	0.043±0.038	0.117±0.021	0.023±0.018	0.330±0.011
Binding_1	**0.560**±0.020	0.549±0.026	0.520±0.028	0.513±0.028	0.314±0.023	0.300±0.057	0.291±0.047	0.451±0.019	0.406±0.026	0.016±0.029	0.392±0.013
Binding_2	**0.544**±0.014	0.521±0.039	0.455±0.019	0.470±0.029	0.245±0.008	0.244±0.011	0.245±0.007	0.444±0.037	0.404±0.003	0.400±0.019	0.363±0.029
eIF4AIII_1	0.792±0.010	**0.812**±0.012	0.741±0.015	0.746±0.020	0.271±0.032	0.315±0.036	0.306±0.030	0.663±0.022	0.645±0.021	0.115±0.039	0.689±0.021
eIF4AIII_2	0.785±0.010	**0.805**±0.009	0.717±0.011	0.732±0.010	0.454±0.031	0.457±0.021	0.476±0.019	0.664±0.027	0.669±0.020	0.272±0.058	0.638±0.010
ELVAL1-1	0.564±0.028	0.597±0.012	0.566±0.013	**0.598**±0.018	0.503±0.027	0.501±0.019	0.484±0.025	0.474±0.075	0.555±0.016	0.401±0.018	0.540±0.023
ELVAL1-2	0.677±0.011	**0.686**±0.019	0.682±0.019	0.671±0.021	0.571±0.013	0.569±0.035	0.553±0.030	0.539±0.009	0.668±0.019	0.409±0.029	0.648±0.027
ELVAL1-A	0.592±0.016	**0.641**±0.021	0.531±0.052	0.616±0.015	0.447±0.015	0.442±0.017	0.434±0.026	0.452±0.021	0.586±0.019	0.340±0.020	0.555±0.023
ELVAL1-M	0.165±0.013	0.170±0.050	0.132±0.049	0.449±0.055	0.302±0.104	0.209±0.023	0.218±0.023	0.066±0.037	0.153±0.031	**0.528**±0.013	0.345±0.040
EWSR1	0.593±0.025	0.603±0.025	0.593±0.050	**0.630**±0.008	0.429±0.056	0.490±0.045	0.420±0.028	0.319±0.033	0.581±0.014	0.225±0.031	0.562±0.027
FUS	0.689±0.016	**0.746**±0.020	0.660±0.028	0.712±0.022	0.544±0.029	0.526±0.024	0.528±0.031	0.322±0.044	0.691±0.021	0.259±0.030	0.651±0.009
hnRNPC-1	0.755±0.013	**0.785**±0.011	0.753±0.012	0.749±0.004	0.624±0.022	0.623±0.010	0.631±0.005	0.576±0.026	0.718±0.015	0.297±0.014	0.709±0.027
hnRNPC-2	**0.804**±0.020	0.801±0.018	0.794±0.009	0.796±0.016	0.738±0.010	0.738±0.017	0.733±0.011	0.648±0.033	0.774±0.012	0.341±0.027	0.784±0.010
hnRNPL-1	0.216±0.019	**0.333**±0.051	0.057±0.037	0.121±0.103	0.323±0.024	0.068±0.139	0.112±0.154	0.160±0.024	0.059±0.030	0.263±0.021	0.272±0.036
hnRNPL-2	0.133±0.060	0.274±0.033	0.059±0.034	0.064±0.122	**0.630**±0.015	0.167±0.096	0.109±0.107	0.086±0.034	0.052±0.036	0.298±0.029	0.323±0.021
HnRNPL-L	0.215±0.035	**0.282**±0.042	0.162±0.028	0.192±0.043	0.266±0.069	0.132±0.030	0.151±0.067	0.166±0.018	0.080±0.040	0.096±0.036	0.235±0.041
IGF2BP1-3	0.266±0.024	0.156±0.042	0.106±0.007	0.115±0.060	0.238±0.014	0.129±0.025	0.129±0.045	0.101±0.032	0.134±0.014	0.046±0.024	**0.566**±0.014
MOV10	0.322±0.044	0.399±0.029	0.291±0.043	0.297±0.042	**0.414**±0.021	0.167±0.052	0.154±0.052	0.304±0.026	0.341±0.030	0.100±0.057	0.264±0.023
mut-FUS	0.703±0.027	**0.742**±0.022	0.673±0.045	0.731±0.025	0.522±0.021	0.494±0.029	0.485±0.024	0.375±0.047	0.738±0.007	0.253±0.036	0.669±0.018
NSUN2	0.362±0.005	**0.456**±0.064	0.359±0.026	0.363±0.105	0.329±0.046	0.297±0.025	0.273±0.025	0.236±0.037	0.259±0.030	0.082±0.024	0.435±0.021
PUM2	**0.796**±0.012	0.774±0.011	0.780±0.010	0.769±0.022	0.491±0.029	0.489±0.019	0.487±0.042	0.638±0.032	0.644±0.022	0.371±0.043	0.708±0.008
QKI	0.800±0.011	**0.821**±0.004	0.821±0.033	0.806±0.015	0.646±0.010	0.622±0.014	0.631±0.020	0.717±0.020	0.810±0.013	0.394±0.043	0.790±0.024
SFRS1	**0.615**±0.010	0.600±0.019	0.555±0.011	0.527±0.028	0.342±0.010	0.118±0.075	0.125±0.073	0.478±0.041	0.448±0.025	0.012±0.043	0.488±0.030
TAF1S	0.744±0.024	**0.783**±0.012	0.616±0.067	0.768±0.017	0.491±0.061	0.496±0.060	0.415±0.075	0.374±0.025	0.751±0.008	0.153±0.028	0.728±0.023
TDP-43	0.660±0.012	**0.684**±0.010	0.670±0.009	0.672±0.005	0.451±0.023	0.468±0.020	0.463±0.013	0.631±0.011	0.656±0.022	0.170±0.038	0.644±0.021
TIA1	0.637±0.008	**0.647**±0.017	0.565±0.024	0.626±0.030	0.571±0.039	0.551±0.019	0.533±0.018	0.427±0.028	0.553±0.017	0.320±0.034	0.596±0.023
TIAL1	0.506±0.015	**0.519**±0.019	0.510±0.022	0.490±0.013	0.463±0.027	0.480±0.028	0.449±0.044	0.435±0.038	0.506±0.016	0.283±0.016	0.504±0.023
U2AF65	0.739±0.016	**0.786**±0.012	0.729±0.030	0.732±0.022	0.642±0.007	0.656±0.008	0.653±0.013	0.434±0.036	0.617±0.011	0.490±0.033	0.682±0.034
Y2AF65	0.653±0.011	**0.673**±0.016	0.591±0.031	0.612±0.022	0.526±0.037	0.541±0.023	0.528±0.033	0.453±0.027	0.572±0.027	0.530±0.036	0.562±0.006
Avg.±Std.	**0.562**±0.020	0.547±0.025	0.483±0.028	0.518±0.033	0.433±0.029	0.381±0.033	0.372±0.038	0.383±0.032	0.464±0.021	0.248±0.030	0.518±0.023

Supplementary Table. 3: Generalizability comparison in terms of ACCs, AUCs, AUPRCs, F1-score and MCCs for 3UTR-BERT, RmLR, BERT_RBP, DNABERT, RNABERT, RNAMSM, RNAFM, GraphProt2, RPI_Net, DeepCLIP and iDeepE on 22 eCLIP protocols under RBP-generic strategy.

Dataset_22_ACCs	3UTRBERT	RmLR	BERT_RBP	DNABERT	RNABERT	RNAMSM	RNAFM	GraphProt2	RPI_Net	DeepCLIP	iDeepE
AKAP1_HepG2	**0.734**	0.680	0.624	0.640	0.631	0.653	0.642	0.638	0.684	0.564	0.688
BCLAF1_HepG2	**0.756**	0.696	0.607	0.624	0.621	0.623	0.624	0.634	0.726	0.550	0.674
DDX24_K562	**0.790**	0.715	0.586	0.625	0.607	0.624	0.616	0.573	0.746	0.525	0.655
DDX3X_HepG2	**0.787**	0.710	0.687	0.658	0.701	0.683	0.698	0.585	0.732	0.505	0.646
DDX3X_K562	**0.757**	0.695	0.638	0.624	0.655	0.659	0.665	0.588	0.705	0.511	0.621
FAM120A_HepG2	**0.721**	0.673	0.618	0.617	0.629	0.650	0.630	0.576	0.685	0.489	0.638
G3BP1_HepG2	**0.735**	0.685	0.610	0.610	0.620	0.615	0.624	0.587	0.710	0.499	0.640
GRWD1_HepG2	**0.725**	0.688	0.599	0.616	0.609	0.612	0.615	0.573	0.713	0.473	0.630
IGF2BP1_HepG2	**0.643**	0.634	0.593	0.579	0.602	0.598	0.609	0.570	0.636	0.495	0.642
LARP4_HepG2	**0.719**	0.685	0.606	0.617	0.610	0.626	0.616	0.589	0.696	0.512	0.644
LIN28B_K562	**0.678**	0.642	0.569	0.580	0.576	0.571	0.576	0.579	0.659	0.534	0.650
PABPC4_K562	**0.694**	0.685	0.607	0.617	0.619	0.633	0.623	0.570	0.692	0.514	0.672
PPIG_HepG2	**0.794**	0.723	0.615	0.631	0.624	0.636	0.624	0.560	0.743	0.512	0.641
PUM2_K562	**0.727**	0.701	0.584	0.597	0.603	0.608	0.613	0.584	0.714	0.491	0.673
RBM15_K562	**0.737**	0.677	0.640	0.627	0.655	0.647	0.655	0.584	0.683	0.513	0.655
RPS3_HepG2	**0.755**	0.675	0.641	0.624	0.648	0.639	0.647	0.595	0.704	0.500	0.624
SND1_HepG2	**0.748**	0.679	0.617	0.611	0.631	0.629	0.636	0.549	0.688	0.458	0.594
UCHL5_K562	**0.740**	0.700	0.580	0.621	0.592	0.614	0.595	0.605	0.736	0.501	0.653
UPF1_HepG2	**0.714**	0.680	0.633	0.638	0.634	0.641	0.637	0.575	0.685	0.469	0.659
UPF1_K562	**0.698**	0.669	0.604	0.619	0.616	0.633	0.620	0.595	0.679	0.504	0.625
YBX3_K562	**0.674**	0.647	0.584	0.591	0.597	0.595	0.601	0.514	0.672	0.473	0.623
ZNF622_K562	**0.739**	0.701	0.582	0.627	0.591	0.612	0.600	0.565	0.729	0.475	0.621
Avg.	**0.730**	0.684	0.610	0.618	0.621	0.627	0.626	0.581	0.701	0.503	0.644

Dataset_22_AUCs	3UTRBERT	RmLR	BERT_RBP	DNABERT	RNABERT	RNAMSM	RNAFM	GraphProt2	RPI_Net	DeepCLIP	iDeepE
AKAP1_HepG2	0.760	0.727	0.680	0.722	0.687	0.724	0.689	0.735	0.684	**0.769**	0.742
BCLAF1_HepG2	**0.820**	0.788	0.646	0.699	0.654	0.677	0.656	0.707	0.698	0.726	0.708
DDX24_K562	**0.851**	0.821	0.635	0.709	0.646	0.678	0.649	0.611	0.709	0.652	0.682
DDX3X_HepG2	**0.806**	0.793	0.772	0.761	0.783	0.774	0.789	0.631	0.722	0.615	0.683
DDX3X_K562	**0.776**	0.766	0.693	0.701	0.713	0.728	0.722	0.637	0.700	0.628	0.603
FAM120A_HepG2	0.725	0.731	0.691	0.689	0.695	**0.732**	0.696	0.622	0.679	0.592	0.662
G3BP1_HepG2	**0.798**	0.763	0.642	0.690	0.645	0.659	0.646	0.660	0.680	0.628	0.675
GRWD1_HepG2	**0.795**	0.762	0.626	0.690	0.631	0.650	0.632	0.616	0.679	0.567	0.646
IGF2BP1_HepG2	0.660	0.656	0.621	0.603	0.642	0.641	0.646	0.608	0.633	0.578	**0.666**
LARP4_HepG2	0.745	**0.754**	0.643	0.697	0.646	0.687	0.645	0.637	0.683	0.642	0.657
LIN28B_K562	**0.721**	0.690	0.569	0.626	0.564	0.570	0.562	0.619	0.646	0.655	0.695
PABPC4_K562	0.732	**0.749**	0.639	0.688	0.640	0.679	0.642	0.612	0.675	0.641	0.700
PPIG_HepG2	**0.859**	0.836	0.659	0.725	0.662	0.695	0.663	0.594	0.716	0.633	0.664
PUM2_K562	0.772	**0.791**	0.587	0.658	0.605	0.637	0.606	0.617	0.681	0.580	0.711
RBM15_K562	**0.738**	0.731	0.710	0.707	0.714	0.722	0.712	0.636	0.683	0.640	0.655
RPS3_HepG2	**0.782**	0.764	0.711	0.707	0.708	0.700	0.707	0.661	0.699	0.621	0.625
SND1_HepG2	**0.769**	0.754	0.682	0.681	0.678	0.685	0.676	0.569	0.687	0.541	0.577
UCHL5_K562	**0.827**	0.791	0.596	0.699	0.599	0.655	0.605	0.670	0.686	0.631	0.681
UPF1_HepG2	0.721	**0.740**	0.687	0.714	0.684	0.706	0.684	0.605	0.675	0.576	0.687
UPF1_K562	0.710	**0.728**	0.650	0.686	0.656	0.690	0.655	0.657	0.665	0.626	0.633
YBX3_K562	**0.719**	0.693	0.590	0.628	0.595	0.608	0.594	0.498	0.660	0.535	0.642
ZNF622_K562	**0.823**	0.783	0.594	0.700	0.598	0.644	0.605	0.586	0.696	0.567	0.606
Avg.	**0.768**	0.755	0.651	0.690	0.657	0.679	0.658	0.627	0.684	0.620	0.664

Dataset_22_AUPRCs	3UTRBERT	RmLR	BERT_RBP	DNABERT	RNABERT	RNAMSM	RNAFM	GraphProt2	RPI_Net	DeepCLIP	iDeepE
AKAP1_HepG2	**0.577**	0.521	0.484	0.532	0.492	0.542	0.493	0.536	0.541	0.577	0.507
BCLAF1_HepG2	**0.747**	0.702	0.496	0.545	0.511	0.521	0.515	0.513	0.651	0.539	0.492
DDX24_K562	**0.788**	0.735	0.454	0.551	0.467	0.504	0.470	0.425	0.668	0.443	0.496
DDX3X_HepG2	**0.620**	0.616	0.535	0.596	0.554	0.589	0.558	0.429	0.588	0.422	0.456
DDX3X_K562	**0.643**	0.607	0.475	0.551	0.496	0.543	0.503	0.471	0.587	0.446	0.497
FAM120A_HepG2	0.571	0.580	0.575	0.541	0.580	**0.597**	0.581	0.458	0.589	0.437	0.525
G3BP1_HepG2	**0.706**	0.642	0.464	0.530	0.468	0.477	0.468	0.482	0.606	0.463	0.509
GRWD1_HepG2	**0.693**	0.640	0.434	0.528	0.439	0.461	0.439	0.428	0.573	0.399	0.455
IGF2BP1_HepG2	**0.526**	0.514	0.467	0.427	0.487	0.477	0.491	0.421	0.507	0.388	0.494
LARP4_HepG2	0.593	**0.613**	0.451	0.552	0.454	0.512	0.454	0.442	0.560	0.439	0.468
LIN28B_K562	**0.596**	0.565	0.394	0.450	0.389	0.404	0.387	0.471	0.516	0.490	0.575
PABPC4_K562	0.582	**0.598**	0.477	0.534	0.477	0.525	0.476	0.417	0.535	0.438	0.516
PPIG_HepG2	**0.791**	0.744	0.449	0.587	0.452	0.495	0.450	0.418	0.671	0.442	0.519
PUM2_K562	0.640	**0.658**	0.483	0.489	0.506	0.504	0.507	0.416	0.614	0.377	0.506
RBM15_K562	0.537	0.531	0.508	0.533	0.508	**0.539**	0.505	0.450	0.535	0.446	0.495
RPS3_HepG2	**0.649**	0.617	0.486	0.546	0.481	0.494	0.478	0.511	0.575	0.464	0.472
SND1_HepG2	**0.649**	0.621	0.444	0.489	0.439	0.460	0.437	0.402	0.581	0.379	0.406
UCHL5_K562	**0.748**	0.692	0.421	0.565	0.430	0.487	0.437	0.479	0.613	0.457	0.473
UPF1_HepG2	0.502	**0.541**	0.487	0.533	0.486	0.524	0.485	0.393	0.527	0.383	0.463
UPF1_K562	0.527	**0.561**	0.494	0.520	0.499	0.526	0.499	0.496	0.531	0.455	0.467
YBX3_K562	**0.586**	0.541	0.378	0.433	0.382	0.397	0.381	0.357	0.495	0.370	0.491
ZNF622_K562	**0.747**	0.691	0.434	0.563	0.442	0.487	0.448	0.368	0.621	0.358	0.392
Avg.	**0.637**	0.615	0.468	0.527	0.474	0.503	0.476	0.445	0.577	0.437	0.485

Dataset_22_F1s	3UTRBERT	RmLR	BERT_RBP	DNABERT	RNABERT	RNAMSM	RNAFM	GraphProt2	RPI_Net	DeepCLIP	iDeepE
AKAP1_HepG2	**0.595**	0.571	0.529	0.576	0.526	0.568	0.533	0.590	0.515	0.591	0.558
BCLAF1_HepG2	**0.648**	0.618	0.503	0.551	0.507	0.524	0.509	0.573	0.536	0.569	0.531
DDX24_K562	**0.670**	0.640	0.472	0.552	0.485	0.521	0.489	0.483	0.541	0.540	0.517
DDX3X_HepG2	**0.650**	0.636	0.641	0.602	0.646	0.630	0.644	0.501	0.566	0.505	0.506
DDX3X_K562	**0.611**	0.608	0.546	0.551	0.559	0.581	0.567	0.511	0.542	0.523	0.460
FAM120A_HepG2	0.576	0.584	0.540	0.549	0.539	**0.586**	0.540	0.485	0.531	0.486	0.505
G3BP1_HepG2	**0.613**	0.592	0.491	0.527	0.487	0.503	0.483	0.514	0.507	0.505	0.493
GRWD1_HepG2	**0.618**	0.590	0.474	0.534	0.473	0.496	0.474	0.480	0.504	0.463	0.471
IGF2BP1_HepG2	0.498	**0.506**	0.475	0.473	0.483	0.493	0.489	0.470	0.457	0.484	0.490
LARP4_HepG2	0.581	**0.591**	0.494	0.535	0.483	0.527	0.480	0.495	0.507	0.514	0.485
LIN28B_K562	**0.548**	0.516	0.419	0.471	0.402	0.423	0.398	0.491	0.457	0.547	0.536
PABPC4_K562	0.568	**0.580**	0.483	0.524	0.487	0.529	0.486	0.474	0.492	0.529	0.533
PPIG_HepG2	**0.677**	0.657	0.510	0.563	0.508	0.540	0.501	0.467	0.554	0.525	0.503
PUM2_K562	0.629	**0.640**	0.462	0.514	0.476	0.504	0.483	0.483	0.525	0.489	0.550
RBM15_K562	**0.587**	0.560	0.556	0.556	0.559	0.563	0.556	0.497	0.518	0.515	0.487
RPS3_HepG2	**0.609**	0.585	0.566	0.554	0.559	0.555	0.552	0.522	0.533	0.504	0.467
SND1_HepG2	**0.565**	0.561	0.506	0.504	0.504	0.510	0.502	0.434	0.489	0.445	0.398
UCHL5_K562	**0.663**	0.621	0.452	0.544	0.449	0.507	0.449	0.533	0.521	0.511	0.518
UPF1_HepG2	0.570	**0.586**	0.548	0.573	0.537	0.559	0.533	0.460	0.512	0.457	0.503
UPF1_K562	0.564	**0.576**	0.501	0.544	0.503	0.548	0.501	0.514	0.501	0.507	0.465
YBX3_K562	**0.518**	0.494	0.414	0.459	0.414	0.434	0.412	0.377	0.438	0.459	0.481
ZNF622_K562	**0.655**	0.622	0.452	0.553	0.451	0.501	0.456	0.432	0.538	0.445	0.418
Avg.	**0.601**	0.588	0.502	0.537	0.502	0.527	0.502	0.490	0.513	0.505	0.494

Dataset_22_MCCs	3UTRBERT	RmLR	BERT_RBP	DNABERT	RNABERT	RNAMSM	RNAFM	GraphProt2	RPI_Net	DeepCLIP	iDeepE
AKAP1_HepG2	**0.359**	0.329	0.243	0.316	0.245	0.309	0.260	0.328	0.326	0.337	0.319
BCLAF1_HepG2	**0.445**	0.393	0.200	0.269	0.215	0.234	0.218	0.302	0.359	0.284	0.282
DDX24_K562	**0.487**	0.434	0.153	0.276	0.183	0.235	0.194	0.150	0.392	0.211	0.252
DDX3X_HepG2	**0.449**	0.421	0.425	0.354	0.434	0.404	0.430	0.184	0.424	0.145	0.235
DDX3X_K562	**0.386**	0.377	0.269	0.267	0.296	0.324	0.310	0.187	0.356	0.160	0.169
FAM120A_HepG2	**0.330**	0.328	0.239	0.249	0.246	0.316	0.248	0.157	0.317	0.100	0.226
G3BP1_HepG2	**0.396**	0.357	0.192	0.234	0.196	0.209	0.196	0.204	0.309	0.148	0.219
GRWD1_HepG2	**0.405**	0.357	0.167	0.247	0.174	0.200	0.179	0.153	0.302	0.063	0.191
IGF2BP1_HepG2	0.216	**0.222**	0.156	0.142	0.171	0.179	0.184	0.139	0.192	0.109	0.218
LARP4_HepG2	0.353	**0.359**	0.195	0.252	0.185	0.246	0.188	0.184	0.302	0.177	0.217
LIN28B_K562	**0.305**	0.243	0.087	0.148	0.077	0.092	0.075	0.155	0.208	0.205	0.261
PABPC4_K562	0.337	**0.347**	0.185	0.239	0.199	0.255	0.201	0.143	0.283	0.199	0.284
PPIG_HepG2	**0.493**	0.458	0.213	0.290	0.218	0.263	0.211	0.120	0.409	0.171	0.226
PUM2_K562	0.409	**0.418**	0.133	0.198	0.165	0.196	0.180	0.165	0.325	0.115	0.302
RBM15_K562	**0.350**	0.316	0.289	0.283	0.301	0.301	0.296	0.186	0.336	0.183	0.228
RPS3_HepG2	**0.387**	0.342	0.300	0.276	0.294	0.284	0.285	0.211	0.360	0.131	0.179
SND1_HepG2	**0.340**	0.330	0.230	0.224	0.235	0.241	0.235	0.084	0.314	0.027	0.092
UCHL5_K562	**0.468**	0.397	0.125	0.257	0.132	0.208	0.135	0.235	0.330	0.152	0.252
UPF1_HepG2	0.328	**0.342**	0.267	0.303	0.254	0.285	0.252	0.148	0.298	0.085	0.252
UPF1_K562	0.317	**0.321**	0.192	0.251	0.204	0.266	0.204	0.203	0.277	0.142	0.178
YBX3_K562	**0.283**	0.239	0.109	0.163	0.119	0.137	0.120	-0.009	0.229	0.040	0.189
ZNF622_K562	**0.453**	0.397	0.124	0.270	0.132	0.199	0.146	0.116	0.349	0.082	0.142
Avg.	**0.377**	0.351	0.204	0.250	0.212	0.245	0.216	0.170	0.318	0.148	0.223

Supplementary Table. 4: Generalizability comparison in terms of ACCs, AUCs, AUPRCs, F1-score and MCCs for 3UTR-BERT, RmLR, BERT_RBP, DNABERT, RNABERT, RNAMSM, RNAFM, GraphProt2, RPI_Net, DeepCLIP and iDeepE in 31 CLIP experiments on 19 RBPs under RBP-generic strategy.

Dataset_31_ACCs	3UTRBERT	RmLR	BERT_RBP	DNABERT	RNABERT	RNAMSM	RNAFM	GraphProt2	RPI_Net	DeepCLIP	iDeepE
AGO1234	0.789	0.788	0.782	0.774	0.776	0.772	0.773	0.783	0.769	0.788	**0.797**
AGO2	0.781	0.771	**0.789**	0.781	0.787	0.777	0.774	0.784	0.786	0.787	0.752
AGO2-M	0.789	0.786	0.799	0.784	0.778	0.777	0.779	0.839	0.782	0.822	**0.857**
Binding_1	0.823	0.805	0.802	0.806	0.779	0.774	0.773	0.818	0.781	0.809	**0.841**
Binding_2	**0.806**	0.806	0.803	0.805	0.772	0.764	0.771	0.789	0.782	0.783	0.785
ELVAL1-1	0.844	**0.848**	0.835	0.842	0.826	0.841	0.835	0.807	0.812	0.801	0.844
ELVAL1-2	0.859	**0.862**	0.862	0.862	0.854	0.858	0.856	0.818	0.813	0.828	0.786
ELVAL1-A	**0.861**	0.841	0.848	0.845	0.837	0.847	0.846	0.845	0.841	0.822	0.846
ELVAL1-M	**0.868**	0.784	0.784	0.790	0.774	0.765	0.770	0.785	0.783	0.791	0.799
EWSR1	0.864	**0.866**	0.854	0.856	0.828	0.864	0.858	0.848	0.846	0.833	0.854
FUS	0.784	0.870	0.849	0.857	0.847	0.863	0.860	0.807	**0.871**	0.796	0.828
HnRNPL-L	**0.854**	0.781	0.798	0.791	0.793	0.792	0.793	0.807	0.790	0.802	0.839
IGF2BP1-3	**0.876**	0.781	0.782	0.782	0.784	0.778	0.776	0.777	0.785	0.782	0.745
MOV10	**0.857**	0.774	0.782	0.787	0.788	0.777	0.776	0.782	0.778	0.791	0.770
NSUN2	**0.870**	0.771	0.784	0.776	0.775	0.760	0.765	0.790	0.776	0.793	0.798
PUM2	0.791	0.858	0.853	0.865	0.821	0.828	0.833	0.805	**0.872**	0.804	0.852
QKI	0.782	0.863	0.880	0.871	0.856	0.870	0.867	0.774	**0.889**	0.788	0.758
SFRS1	0.790	0.806	0.807	0.800	0.768	0.767	0.769	0.792	**0.816**	0.793	0.765
TAF1S	**0.893**	0.892	0.876	0.886	0.845	0.873	0.859	0.792	0.774	0.800	0.775
TDP-43	**0.837**	0.808	0.828	0.826	0.788	0.784	0.787	0.786	0.773	0.791	0.768
TIA1	**0.873**	0.867	0.849	0.866	0.841	0.836	0.833	0.794	0.866	0.789	0.770
TIAL1	**0.847**	0.835	0.835	0.844	0.837	0.839	0.831	0.823	0.767	0.813	0.833
U2AF65	0.875	**0.876**	0.852	0.870	0.854	0.845	0.847	0.828	0.829	0.809	0.842
Y2AF65	**0.886**	0.836	0.825	0.834	0.834	0.823	0.824	0.807	0.833	0.797	0.844
eIF4AIII_1	**0.884**	0.870	0.853	0.860	0.769	0.763	0.768	0.816	0.782	0.810	0.836
eIF4AIII_2	**0.898**	0.878	0.844	0.853	0.765	0.760	0.765	0.767	0.868	0.786	0.752
hnRNPC-1	0.838	0.832	0.832	0.837	0.839	**0.854**	0.844	0.792	0.831	0.789	0.781
hnRNPC-2	0.872	0.847	0.844	0.852	**0.874**	0.873	0.865	0.842	0.849	0.825	0.843
hnRNPL-1	0.839	0.777	0.790	0.799	0.797	0.798	0.795	0.828	0.799	0.812	**0.861**
hnRNPL-2	**0.860**	0.777	0.788	0.785	0.797	0.797	0.797	0.787	0.785	0.787	0.756
mut-FUS	**0.896**	0.875	0.877	0.879	0.844	0.874	0.862	0.812	0.828	0.811	0.873
Avg.	**0.853**	0.834	0.831	0.835	0.816	0.819	0.817	0.802	0.819	0.800	0.806

Dataset_31_AUCs	3UTRBERT	RmLR	BERT_RBP	DNABERT	RNABERT	RNAMSM	RNAFM	GraphProt2	RPI_Net	DeepCLIP	iDeepE
AGO1234	**0.759**	0.753	0.688	0.715	0.603	0.606	0.600	0.615	0.689	0.596	0.629
AGO2	0.628	0.605	0.567	0.591	0.620	0.630	0.619	**0.693**	0.600	0.654	0.322
AGO2-M	0.627	0.627	0.622	0.628	0.561	0.566	0.566	0.857	0.610	0.829	**0.889**
Binding_1	0.814	0.805	0.768	0.769	0.628	0.625	0.629	0.797	0.717	0.792	**0.894**
Binding_2	**0.810**	0.799	0.765	0.759	0.607	0.612	0.615	0.628	0.723	0.628	0.612
ELVAL1-1	0.873	0.858	0.855	0.849	0.883	**0.890**	0.881	0.787	0.836	0.785	0.865
ELVAL1-2	0.885	**0.887**	0.859	0.872	0.883	0.887	0.883	0.759	0.796	0.786	0.689
ELVAL1-A	0.832	0.826	0.805	0.823	0.831	0.834	0.822	**0.883**	0.878	0.874	0.876
ELVAL1-M	**0.882**	0.590	0.602	0.612	0.465	0.468	0.478	0.640	0.613	0.654	0.693
EWSR1	**0.885**	0.873	0.852	0.844	0.851	0.873	0.873	0.881	0.821	0.866	0.870
FUS	0.613	0.892	0.857	0.853	0.868	0.905	0.900	0.785	**0.906**	0.774	0.854
HnRNPL-L	**0.889**	0.677	0.670	0.659	0.651	0.655	0.643	0.811	0.679	0.797	0.878
IGF2BP1-3	**0.903**	0.672	0.646	0.653	0.586	0.585	0.587	0.416	0.686	0.398	0.314
MOV10	**0.875**	0.729	0.668	0.700	0.751	0.752	0.743	0.711	0.733	0.703	0.590
NSUN2	**0.913**	0.615	0.618	0.618	0.279	0.280	0.281	0.631	0.618	0.622	0.705
PUM2	0.660	0.901	0.878	0.875	0.875	0.875	0.875	0.795	**0.908**	0.792	0.877
QKI	0.661	0.892	0.892	0.870	0.872	0.899	0.895	0.537	**0.901**	0.527	0.457
SFRS1	0.644	0.753	0.750	0.721	0.296	0.350	0.402	0.689	**0.762**	0.653	0.618
TAF1S	**0.925**	0.916	0.903	0.906	0.881	0.913	0.910	0.716	0.632	0.706	0.609
TDP-43	**0.804**	0.789	0.779	0.762	0.736	0.729	0.683	0.636	0.679	0.618	0.612
TIA1	**0.895**	0.882	0.859	0.879	0.882	0.871	0.860	0.617	0.891	0.607	0.539
TIAL1	0.852	0.837	0.809	0.810	**0.868**	0.853	0.842	0.823	0.491	0.800	0.867
U2AF65	0.916	0.914	0.902	0.905	**0.919**	0.897	0.891	0.835	0.873	0.819	0.811
Y2AF65	**0.918**	0.868	0.840	0.842	0.881	0.872	0.861	0.791	0.867	0.764	0.882
eIF4AIII_1	0.899	**0.901**	0.837	0.850	0.368	0.431	0.504	0.818	0.698	0.796	0.883
eIF4AIII_2	**0.929**	0.873	0.819	0.823	0.366	0.438	0.516	0.599	0.851	0.556	0.269
hnRNPC-1	0.869	0.840	0.834	0.833	0.902	**0.904**	0.882	0.756	0.789	0.747	0.723
hnRNPC-2	0.910	0.873	0.887	0.876	0.926	**0.927**	0.914	0.876	0.858	0.873	0.886
hnRNPL-1	0.834	0.696	0.681	0.667	0.714	0.726	0.719	0.841	0.699	0.829	**0.921**
hnRNPL-2	**0.892**	0.682	0.654	0.667	0.718	0.720	0.711	0.754	0.685	0.718	0.337
mut-FUS	0.916	0.909	0.900	0.895	0.858	0.888	0.887	0.841	0.861	0.823	**0.917**
Avg.	**0.829**	0.798	0.776	0.778	0.714	0.724	0.725	0.736	0.753	0.722	0.706

Dataset_31_AUPRCs	3UTRBERT	RmLR	BERT_RBP	DNABERT	RNABERT	RNAMSM	RNAFM	GraphProt2	RPI_Net	DeepCLIP	iDeepE
AGO1234	**0.405**	0.374	0.315	0.334	0.237	0.240	0.235	0.283	0.299	0.266	0.320
AGO2	0.292	0.283	0.267	0.279	0.265	0.276	0.267	**0.317**	0.270	0.274	0.140
AGO2-M	0.332	0.317	0.320	0.308	0.227	0.234	0.234	0.592	0.280	0.558	**0.704**
Binding_1	0.504	0.478	0.440	0.443	0.281	0.277	0.281	0.493	0.344	0.478	**0.669**
Binding_2	**0.480**	0.468	0.438	0.421	0.263	0.261	0.259	0.293	0.360	0.287	0.264
ELVAL1-1	0.616	0.584	0.609	0.604	0.600	**0.632**	0.612	0.470	0.537	0.433	0.625
ELVAL1-2	0.670	0.678	0.656	0.669	0.695	0.710	**0.713**	0.451	0.480	0.520	0.338
ELVAL1-A	**0.645**	0.623	0.593	0.615	0.584	0.611	0.594	0.636	0.623	0.590	0.582
ELVAL1-M	**0.684**	0.269	0.261	0.275	0.178	0.179	0.181	0.293	0.295	0.293	0.358
EWSR1	**0.681**	0.680	0.632	0.630	0.573	0.616	0.627	0.665	0.601	0.629	0.658
FUS	0.295	0.708	0.675	0.683	0.642	0.709	0.700	0.438	**0.727**	0.419	0.537
HnRNPL-L	**0.645**	0.359	0.343	0.348	0.330	0.337	0.320	0.477	0.366	0.443	0.614
IGF2BP1-3	**0.722**	0.318	0.303	0.314	0.245	0.239	0.243	0.164	0.327	0.157	0.138
MOV10	**0.632**	0.347	0.305	0.352	0.327	0.325	0.321	0.359	0.352	0.352	0.244
NSUN2	**0.726**	0.270	0.266	0.265	0.132	0.132	0.133	0.298	0.265	0.301	0.377
PUM2	0.329	0.644	0.662	0.648	0.556	0.574	0.569	0.444	**0.713**	0.437	0.647
QKI	0.338	0.696	0.754	0.695	0.644	0.711	0.721	0.226	**0.780**	0.226	0.176
SFRS1	0.314	0.443	0.435	0.418	0.135	0.145	0.156	0.325	**0.495**	0.290	0.241
TAF1S	**0.786**	0.773	0.747	0.745	0.662	0.711	0.716	0.359	0.281	0.366	0.260
TDP-43	**0.524**	0.455	0.496	0.484	0.347	0.365	0.333	0.282	0.303	0.257	0.254
TIA1	0.678	0.682	0.647	0.658	0.642	0.626	0.615	0.291	**0.700**	0.265	0.219
TIAL1	0.596	0.580	0.569	0.570	0.589	0.595	0.591	0.548	0.190	0.502	**0.611**
U2AF65	**0.725**	0.721	0.710	0.713	0.701	0.651	0.672	0.544	0.583	0.493	0.560
Y2AF65	**0.745**	0.574	0.560	0.562	0.614	0.572	0.587	0.464	0.608	0.403	0.641
eIF4AIII_1	**0.748**	0.716	0.643	0.658	0.148	0.162	0.183	0.494	0.349	0.448	0.593
eIF4AIII_2	**0.790**	0.730	0.621	0.640	0.148	0.164	0.186	0.239	0.659	0.221	0.131
hnRNPC-1	0.573	0.561	0.615	0.591	0.671	**0.684**	0.664	0.384	0.562	0.369	0.321
hnRNPC-2	0.670	0.600	0.658	0.618	0.737	**0.744**	0.715	0.629	0.660	0.610	0.592
hnRNPL-1	0.566	0.346	0.350	0.372	0.378	0.373	0.363	0.560	0.381	0.531	**0.741**
hnRNPL-2	**0.693**	0.347	0.322	0.346	0.382	0.393	0.370	0.388	0.352	0.309	0.142
mut-FUS	0.724	0.707	0.720	0.706	0.612	0.644	0.652	0.527	0.588	0.507	**0.733**
Avg.	**0.585**	0.527	0.514	0.515	0.437	0.448	0.446	0.417	0.462	0.395	0.433

Dataset_31_F1s	3UTRBERT	RmLR	BERT_RBP	DNABERT	RNABERT	RNAMSM	RNAFM	GraphProt2	RPI_Net	DeepCLIP	iDeepE
AGO1234	**0.360**	0.326	0.084	0.175	0.043	0.058	0.026	0.142	0.087	0.102	0.210
AGO2	**0.211**	0.191	0.124	0.161	0.078	0.082	0.058	0.150	0.158	0.053	0.208
AGO2-M	0.252	0.221	0.152	0.176	0.059	0.090	0.090	0.443	0.128	0.276	**0.555**
Binding_1	**0.487**	0.423	0.208	0.322	0.075	0.089	0.058	0.321	0.161	0.201	0.472
Binding_2	**0.480**	0.390	0.215	0.335	0.050	0.071	0.073	0.124	0.174	0.036	0.130
ELVAL1-1	0.541	**0.568**	0.421	0.532	0.435	0.514	0.486	0.288	0.390	0.160	0.480
ELVAL1-2	0.618	**0.627**	0.549	0.599	0.510	0.559	0.544	0.326	0.370	0.328	0.195
ELVAL1-A	**0.613**	0.547	0.483	0.529	0.444	0.526	0.500	0.478	0.525	0.288	0.510
ELVAL1-M	**0.602**	0.194	0.085	0.139	0.009	0.017	0.009	0.115	0.234	0.063	0.199
EWSR1	**0.655**	0.605	0.538	0.579	0.411	0.598	0.559	0.493	0.525	0.345	0.510
FUS	0.234	**0.639**	0.470	0.560	0.474	0.573	0.551	0.293	0.605	0.128	0.445
HnRNPL-L	**0.568**	0.300	0.205	0.272	0.162	0.194	0.188	0.328	0.362	0.208	0.489
IGF2BP1-3	**0.629**	0.232	0.114	0.187	0.085	0.090	0.067	0.035	0.304	0.007	0.008
MOV10	**0.534**	0.270	0.128	0.226	0.078	0.119	0.089	0.168	0.271	0.147	0.080
NSUN2	**0.641**	0.149	0.036	0.082	0.009	0.017	0.009	0.167	0.229	0.080	0.211
PUM2	0.234	0.626	0.512	0.604	0.405	0.442	0.456	0.275	**0.680**	0.148	0.507
QKI	0.243	0.657	0.651	0.639	0.526	0.624	0.596	0.058	**0.691**	0.054	0.055
SFRS1	0.216	0.405	0.225	0.301	0.009	0.017	0.009	0.161	**0.421**	0.072	0.071
TAF1S	0.655	**0.725**	0.624	0.689	0.475	0.619	0.555	0.241	0.117	0.174	0.111
TDP-43	**0.463**	0.389	0.394	0.439	0.138	0.150	0.138	0.108	0.143	0.071	0.101
TIA1	**0.661**	0.621	0.459	0.596	0.450	0.442	0.422	0.134	0.615	0.079	0.102
TIAL1	**0.523**	0.522	0.409	0.497	0.416	0.419	0.372	0.361	0.033	0.230	0.430
U2AF65	0.623	**0.639**	0.482	0.608	0.493	0.471	0.478	0.419	0.474	0.268	0.487
Y2AF65	**0.689**	0.534	0.354	0.488	0.435	0.416	0.417	0.249	0.545	0.098	0.473
eIF4AIII_1	**0.700**	0.675	0.518	0.583	0.009	0.017	0.090	0.343	0.162	0.234	0.457
eIF4AIII_2	**0.730**	0.686	0.466	0.553	0.009	0.017	0.090	0.064	0.681	0.045	0.045
hnRNPC-1	**0.509**	0.488	0.373	0.462	0.435	0.507	0.447	0.181	0.460	0.102	0.134
hnRNPC-2	0.560	0.583	0.466	0.580	0.627	**0.632**	0.590	0.484	0.524	0.330	0.526
hnRNPL-1	0.472	0.319	0.210	0.323	0.178	0.217	0.183	0.358	0.337	0.168	**0.559**
hnRNPL-2	**0.565**	0.269	0.197	0.261	0.222	0.251	0.228	0.152	0.292	0.045	0.152
mut-FUS	**0.674**	0.663	0.617	0.665	0.483	0.634	0.574	0.324	0.456	0.235	0.630
Avg.	**0.514**	0.467	0.347	0.424	0.266	0.305	0.289	0.251	0.360	0.154	0.308

Dataset_31_MCCs	3UTRBERT	RmLR	BERT_RBP	DNABERT	RNABERT	RNAMSM	RNAFM	GraphProt2	RPI_Net	DeepCLIP	iDeepE
AGO1234	**0.269**	0.224	0.031	0.088	-0.025	-0.017	-0.050	0.083	0.004	0.064	0.168
AGO2	**0.133**	0.094	0.086	0.092	0.040	0.017	-0.013	0.093	0.102	0.016	0.112
AGO2-M	0.168	0.151	0.140	0.110	-0.003	0.024	0.030	0.406	0.069	0.296	**0.497**
Binding_1	0.394	0.353	0.183	0.257	0.015	0.016	-0.015	0.292	0.092	0.209	**0.421**
Binding_2	**0.375**	0.298	0.191	0.262	-0.025	-0.020	-0.005	0.086	0.104	0.016	0.078
ELVAL1-1	0.449	**0.485**	0.386	0.452	0.366	0.440	0.412	0.241	0.309	0.154	0.433
ELVAL1-2	0.536	**0.546**	0.510	0.528	0.474	0.501	0.490	0.294	0.301	0.335	0.129
ELVAL1-A	**0.535**	0.455	0.447	0.456	0.399	0.460	0.446	0.436	0.446	0.298	0.450
ELVAL1-M	**0.544**	0.106	0.038	0.100	-0.070	-0.075	-0.077	0.066	0.151	0.040	0.168
EWSR1	**0.572**	0.515	0.482	0.506	0.360	0.532	0.501	0.450	0.457	0.362	0.474
FUS	0.151	**0.566**	0.448	0.499	0.442	0.520	0.504	0.244	0.526	0.113	0.376
HnRNPL-L	**0.496**	0.193	0.168	0.192	0.125	0.143	0.143	0.262	0.274	0.184	0.423
IGF2BP1-3	**0.574**	0.143	0.057	0.113	0.038	0.027	0.030	-0.032	0.219	0.068	0.112
MOV10	**0.490**	0.160	0.069	0.152	0.044	0.049	0.021	0.099	0.165	0.109	0.048
NSUN2	**0.567**	0.061	-0.013	0.015	-0.080	-0.102	-0.095	0.119	0.150	0.064	0.172
PUM2	0.167	0.540	0.471	0.537	0.339	0.374	0.393	0.229	**0.602**	0.161	0.467
QKI	0.154	0.571	0.592	0.568	0.485	0.558	0.538	-0.013	**0.612**	0.020	0.046
SFRS1	0.153	0.313	0.211	0.230	-0.091	-0.092	-0.089	0.122	**0.336**	0.057	0.018
TAF1S	0.570	**0.658**	0.572	0.624	0.435	0.562	0.502	0.174	0.041	0.157	0.038
TDP-43	0.365	0.300	0.353	**0.367**	0.093	0.091	0.090	0.064	0.061	0.048	0.015
TIA1	**0.586**	0.550	0.447	0.536	0.414	0.396	0.379	0.110	0.543	0.048	0.020
TIAL1	0.436	0.433	0.382	**0.440**	0.391	0.400	0.357	0.324	-0.053	0.239	0.382
U2AF65	0.553	**0.578**	0.462	0.551	0.471	0.434	0.442	0.363	0.393	0.238	0.430
Y2AF65	**0.624**	0.443	0.329	0.411	0.387	0.349	0.352	0.222	0.453	0.096	0.431
eIF4AIII_1	**0.628**	0.600	0.473	0.516	-0.089	-0.098	-0.091	0.295	0.095	0.227	0.401
eIF4AIII_2	**0.669**	0.610	0.429	0.486	-0.095	-0.102	-0.095	-0.021	0.608	0.003	0.112
hnRNPC-1	0.431	0.407	0.361	0.406	0.403	**0.473**	0.425	0.135	0.390	0.068	0.071
hnRNPC-2	0.471	0.493	0.429	0.499	**0.568**	0.568	0.531	0.429	0.464	0.322	0.451
hnRNPL-1	0.416	0.214	0.149	0.242	0.149	0.175	0.145	0.342	0.225	0.217	**0.509**
hnRNPL-2	**0.509**	0.163	0.135	0.172	0.175	0.193	0.179	0.103	0.180	0.007	0.107
mut-FUS	**0.597**	0.590	0.573	0.597	0.434	0.571	0.517	0.274	0.381	0.232	0.567
Avg.	**0.438**	0.381	0.309	0.355	0.212	0.238	0.223	0.203	0.281	0.144	0.262

Supplementary Table. 5: Comparison results in terms of average AUCs and AUPRCs with Std of RNA-based language models on coding sequence regions.

Dataset_CDs_AUCs	3UTRBERT	RmLR	BERT_RBP	DNABERT	RNABERT	RNAMSM	RNAFM
DDX24_K562	0.755±0.002	**0.777**±0.006	0.719±0.004	0.769±0.005	0.627±0.001	0.643±0.003	0.641±0.003
G3BP1_HepG2	0.830±0.002	**0.844**±0.008	0.819±0.005	0.837±0.003	0.718±0.004	0.720±0.001	0.720±0.001
GRWD1_HepG2	0.844±0.003	**0.858**±0.004	0.832±0.007	0.847±0.003	0.705±0.007	0.706±0.001	0.706±0.001
PPIG_HepG2	0.853±0.002	**0.862**±0.006	0.837±0.003	0.854±0.004	0.687±0.008	0.709±0.002	0.709±0.001
RPS3_HepG2	0.781±0.001	**0.797**±0.004	0.756±0.003	0.790±0.007	0.609±0.006	0.614±0.002	0.598±0.012
SND1_HepG2	**0.795**±0.003	0.789±0.003	0.740±0.009	0.777±0.007	0.565±0.008	0.740±0.009	0.623±0.010
Avg.±Std.	0.810 ±0.002	**0.821**±0.005	0.784 ±0.005	0.812±0.005	0.652±0.006	0.688±0.003	0.666±0.004

Dataset_CDs_AUPRCs	3UTRBERT	RmLR	BERT_RBP	DNABERT	RNABERT	RNAMSM	RNAFM
DDX24_K562	0.729±0.004	**0.759**±0.005	0.690±0.006	0.748±0.008	0.627±0.003	0.636±0.002	0.635±0.002
G3BP1_HepG2	0.827±0.002	**0.839**±0.007	0.813±0.004	0.835±0.005	0.685±0.004	0.691±0.001	0.692±0.003
GRWD1_HepG2	0.835±0.003	**0.855**±0.005	0.826±0.005	0.844±0.003	0.679±0.003	0.685±0.003	0.685±0.002
PPIG_HepG2	0.831±0.002	**0.845**±0.008	0.818±0.003	0.838±0.006	0.656±0.002	0.679±0.002	0.678±0.002
RPS3_HepG2	0.774±0.003	**0.806**±0.002	0.751±0.006	0.794±0.007	0.644±0.010	0.610±0.004	0.601±0.009
SND1_HepG2	**0.804**±0.006	0.783±0.002	0.724±0.009	0.765±0.009	0.557±0.002	0.724±0.009	0.621±0.010
Avg.±Std.	0.800 ±0.003	**0.815** ±0.005	0.770±0.006	0.804±0.006	0.641±0.004	0.671±0.004	0.652±0.005

Supplementary Table. 6: Comparison results in terms of average AUCs and AUPRCs with Std of RNA-based language models on 5' untranslated regions.

Dataset_5UTR_AUCs	3UTRBERT	RmLR	BERT_RBP	DNABERT	RNABERT	RNAMSM	RNAFM
DDX3X_HepG2	**0.912**±0.003	0.899±0.001	0.886±0.007	0.888±0.002	0.788±0.000	0.801±0.001	0.801±0.001
EIF3G_K562	**0.864**±0.004	0.856±0.002	0.827±0.006	0.835±0.007	0.648±0.001	0.673±0.001	0.668±0.002
GEMIN5_K562	**0.834**±0.003	0.822±0.002	0.798±0.005	0.811±0.003	0.671±0.001	0.689±0.004	0.690±0.004
NCBP2_HepG2	**0.919**±0.004	0.911±0.004	0.897±0.006	0.899±0.006	0.777±0.002	0.794±0.004	0.792±0.005
NCBP2_K562	**0.842**±0.004	0.839±0.001	0.814±0.005	0.828±0.005	0.732±0.001	0.738±0.000	0.738±0.001
SERBP1_K562	**0.830**±0.006	0.823±0.007	0.813±0.002	0.824±0.004	0.748±0.001	0.766±0.001	0.762±0.002
Avg.±Std.	**0.867**±0.004	0.858±0.003	0.839±0.005	0.848±0.004	0.727±0.001	0.744±0.002	0.742±0.002

Dataset_5UTR_AUPRCs	3UTRBERT	RmLR	BERT_RBP	DNABERT	RNABERT	RNAMSM	RNAFM
DDX3X_HepG2	**0.918**±0.003	0.905±0.001	0.894±0.006	0.896±0.003	0.800±0.000	0.805±0.001	0.804±0.001
EIF3G_K562	**0.865**±0.004	0.856±0.002	0.838±0.008	0.845±0.007	0.654±0.001	0.677±0.001	0.672±0.001
GEMIN5_K562	**0.844**±0.003	0.837±0.002	0.816±0.004	0.825±0.003	0.681±0.002	0.691±0.002	0.693±0.002
NCBP2_HepG2	**0.928**±0.004	0.919±0.004	0.912±0.004	0.909±0.003	0.802±0.001	0.812±0.002	0.811±0.002
NCBP2_K562	**0.855**±0.004	0.849±0.002	0.833±0.006	0.844±0.005	0.751±0.000	0.751±0.002	0.753±0.001
SERBP1_K562	**0.835**±0.005	0.825±0.006	0.816±0.002	0.831±0.003	0.760±0.000	0.776±0.002	0.772±0.002
Avg.±Std.	**0.874**±0.004	0.865±0.003	0.852±0.005	0.858±0.004	0.741±0.001	0.752±0.002	0.751±0.002

Supplementary Table. 7: Performance evaluation in terms of average AUC, AUPRC, F1-score, MCC and ACC with Std for 3UTRBERT, DeepM6ASeq, iMRM, WHISTLE, and SCRAMP on human m6A modifications across nine cell lines.

**AUC**	3UTRBERT	DeepM6ASeq	WHISTLE	iMRM	SCRAMP
A549	**0.979**±0.003	0.941±0.008	0.879±0.002	0.918±0.006	0.912±0.003
CD8T	**0.979**±0.003	0.959±0.003	0.861±0.001	0.906±0.005	0.685±0.007
ESC	**0.995**±0.002	0.973±0.002	0.567±0.002	0.881±0.009	0.589±0.013
HCT116	**0.978**±0.004	0.931±0.008	0.771±0.002	0.894±0.007	0.586±0.011
HEK293	**0.980**±0.001	0.959±0.004	0.854±0.001	0.909±0.005	0.882±0.005
HEK293T	**0.981**±0.001	0.960±0.001	0.699±0.001	0.889±0.003	0.563±0.004
Hela	**0.978**±0.002	0.956±0.003	0.796±0.002	0.895±0.004	0.606±0.007
HepG2	**0.974**±0.002	0.950±0.002	0.638±0.002	0.896±0.005	0.518±0.009
MOLM13	**0.985**±0.001	0.944±0.002	0.919±0.001	0.929±0.003	0.937±0.002
Avg. ± Std.	**0.981**±0.002	0.953±0.004	0.776±0.001	0.902±0.005	0.698±0.007

**AUPRC**	3UTRBERT	DeepM6ASeq	WHISTLE	iMRM	SCRAMP
A549	**0.965**±0.004	0.937±0.009	0.879±0.003	0.875±0.008	0.865±0.009
CD8T	**0.970**±0.002	0.965±0.004	0.858±0.001	0.849±0.008	0.617±0.032
ESC	**0.992**±0.002	0.982±0.007	0.554±0.010	0.906±0.092	0.640±0.031
HCT116	**0.970**±0.004	0.936±0.010	0.769±0.004	0.833±0.011	0.526±0.068
HEK293	**0.967**±0.002	0.966±0.010	0.852±0.002	0.862±0.008	0.824±0.013
HEK293T	**0.965**±0.001	0.954±0.002	0.693±0.003	0.820±0.004	0.468±0.012
Hela	**0.968**±0.002	0.956±0.003	0.794±0.003	0.842±0.006	0.555±0.031
HepG2	**0.965**±0.002	0.943±0.002	0.631±0.006	0.831±0.007	0.420±0.056
MOLM13	**0.969**±0.001	0.964±0.003	0.915±0.001	0.889±0.006	0.887±0.009
Avg. ± Std.	**0.970**±0.002	0.956±0.006	0.772±0.004	0.856±0.017	0.645±0.029

**F1**	3UTRBERT	DeepM6ASeq	WHISTLE	iMRM	SCRAMP
A549	**0.963**±0.004	0.905±0.008	0.860±0.002	0.863±0.003	0.851±0.005
CD8T	**0.965**±0.003	0.947±0.003	0.838±0.001	0.839±0.006	0.580±0.023
ESC	**0.991**±0.002	0.960±0.005	0.237±0.004	0.790±0.026	0.626±0.017
HCT116	**0.969**±0.004	0.907±0.008	0.703±0.002	0.821±0.009	0.478±0.054
HEK293	**0.965**±0.002	0.941±0.006	0.827±0.001	0.848±0.005	0.806±0.006
HEK293T	**0.964**±0.001	0.956±0.002	0.572±0.001	0.808±0.004	0.404±0.016
Hela	**0.965**±0.003	0.946±0.003	0.744±0.002	0.832±0.004	0.540±0.024
HepG2	**0.965**±0.002	0.945±0.002	0.438±0.001	0.817±0.006	0.348±0.076
MOLM13	**0.965**±0.001	0.945±0.002	0.910±0.001	0.882±0.004	0.873±0.003
Avg. ± Std.	**0.968**±0.002	0.939±0.004	0.681±0.002	0.833±0.007	0.612±0.025

**MCC**	3UTRBERT	DeepM6ASeq	WHISTLE	iMRM	SCRAMP
A549	**0.928**±0.007	0.849±0.017	0.776±0.005	0.751±0.008	0.728±0.009
CD8T	**0.931**±0.005	0.913±0.005	0.747±0.002	0.712±0.010	0.316±0.014
ESC	**0.983**±0.004	0.941±0.009	0.257±0.008	0.639±0.016	0.172±0.022
HCT116	**0.939**±0.008	0.833±0.017	0.602±0.006	0.692±0.017	0.169±0.024
HEK293	**0.932**±0.004	0.911±0.013	0.735±0.003	0.725±0.008	0.648±0.008
HEK293T	**0.929**±0.001	0.912±0.003	0.490±0.004	0.668±0.007	0.126±0.007
Hela	**0.933**±0.005	0.913±0.006	0.642±0.005	0.701±0.007	0.183±0.015
HepG2	**0.932**±0.004	0.911±0.004	0.388±0.007	0.684±0.011	0.075±0.010
MOLM13	**0.931**±0.003	0.909±0.005	0.843±0.002	0.776±0.007	0.757±0.007
Avg. ± Std.	**0.938**±0.004	0.899±0.009	0.609±0.005	0.705±0.010	0.353±0.013

**ACC**	3UTRBERT	DeepM6ASeq	WHISTLE	iMRM	SCRAMP
A549	**0.963**±0.004	0.913±0.008	0.877±0.002	0.873±0.004	0.862±0.005
CD8T	**0.965**±0.003	0.946±0.003	0.860±0.001	0.852±0.005	0.650±0.005
ESC	**0.991**±0.002	0.960±0.005	0.565±0.002	0.808±0.009	0.582±0.011
HCT116	**0.969**±0.004	0.904±0.009	0.769±0.002	0.839±0.008	0.577±0.007
HEK293	**0.965**±0.002	0.939±0.007	0.852±0.001	0.859±0.004	0.820±0.004
HEK293T	**0.964**±0.001	0.945±0.002	0.698±0.001	0.827±0.003	0.554±0.004
Hela	**0.966**±0.003	0.945±0.003	0.795±0.002	0.846±0.004	0.589±0.006
HepG2	**0.965**±0.002	0.944±0.002	0.637±0.002	0.835±0.006	0.531±0.006
MOLM13	**0.965**±0.001	0.954±0.003	0.917±0.001	0.887±0.004	0.878±0.003
Avg. ± Std.	**0.968**±0.002	0.939±0.005	0.774±0.001	0.847±0.005	0.671±0.006

Supplementary Table. 8: Robustness comparison of different prediction methods under cross-cell line conditions with five types of measurements.

Cross-cell-single-3UTRBERT	ACC	AUROC	F1-score	MCC	AUPRC	Avg.
A549-to-CD8T	0.980	0.990	0.971	0.956	0.973	0.974
A549-to-HCT116	0.960	0.973	0.951	0.917	0.929	0.946
CD8T-to-A549	0.962	0.978	0.954	0.924	0.947	0.953
CD8T-to-HCT116	0.962	0.979	0.950	0.922	0.946	0.952
HCT116-to-A549	0.963	0.976	0.952	0.926	0.935	0.950
HCT116-to-CD8T	0.980	0.987	0.971	0.956	0.957	0.970

Cross-cell-single-DeepM6ASeq	ACC	AUROC	F1-score	MCC	AUPRC	Avg.
A549-to-CD8T	0.779	0.899	0.745	0.612	0.757	0.758
A549-to-HCT116	0.750	0.826	0.742	0.545	0.689	0.710
CD8T-to-A549	0.796	0.911	0.779	0.648	0.812	0.789
CD8T-to-HCT116	0.781	0.864	0.779	0.621	0.736	0.756
HCT116-to-A549	0.747	0.846	0.727	0.549	0.690	0.712
HCT116-to-CD8T	0.748	0.854	0.709	0.544	0.667	0.704

Cross-cell-single-iMRM	ACC	AUROC	F1-score	MCC	AUPRC	Avg.
A549-to-CD8T	0.843	0.909	0.763	0.645	0.861	0.804
A549-to-HCT116	0.838	0.897	0.780	0.659	0.859	0.807
CD8T-to-A549	0.863	0.910	0.808	0.703	0.845	0.826
CD8T-to-HCT116	0.838	0.897	0.780	0.659	0.859	0.807
HCT116-to-A549	0.863	0.910	0.808	0.703	0.845	0.826
HCT116-to-CD8T	0.843	0.909	0.763	0.645	0.861	0.804

Cross-cell-single-SCRAMP	ACC	AUROC	F1-score	MCC	AUPRC	Avg.
A549-to-CD8T	0.883	0.518	0.938	-0.059	0.932	0.642
A549-to-HCT116	0.922	0.671	0.960	0.030	0.964	0.710
CD8T-to-A549	0.883	0.518	0.938	-0.059	0.932	0.642
CD8T-to-HCT116	0.915	0.715	0.955	0.258	0.957	0.760
HCT116-to-A549	0.922	0.671	0.960	0.030	0.964	0.710
HCT116-to-CD8T	0.915	0.715	0.955	0.258	0.957	0.760

Cross-cell-single-WHISTLE	ACC	AUROC	F1-score	MCC	AUPRC	Avg.
A549-to-CD8T	0.798	0.763	0.671	0.614	0.720	0.713
A549-to-HCT116	0.900	0.859	0.827	0.783	0.814	0.837
CD8T-to-A549	0.889	0.866	0.830	0.775	0.835	0.839
CD8T-to-HCT116	0.798	0.763	0.671	0.614	0.720	0.713
HCT116-to-A549	0.889	0.866	0.830	0.775	0.835	0.839
HCT116-to-CD8T	0.900	0.859	0.827	0.783	0.814	0.837

References

[1] R. Pearce, G. S. Omenn, Y. Zhang, *bioRxiv* **2022**, 2022–05.

[2] M. Varadi, S. Anyango, M. Deshpande, S. Nair, C. Natassia, G. Yordanova, D. Yuan, O. Stroe, G. Wood, A. Laydon, *Nucleic acids research* **2022**, *50*, D1 D439.

[3] D. Kozakov, D. R. Hall, B. Xia, K. A. Porter, D. Padhorny, C. Yueh, D. Beglov, S. Vajda, *Nature protocols* **2017**, *12*, 2 255.

[4] S. Jo, T. Kim, V. G. Iyer, W. Im, *Journal of computational chemistry* **2008**, *29*, 11 1859.

[5] B. R. Brooks, C. L. Brooks III, A. D. Mackerell Jr, L. Nilsson, R. J. Petrella, B. Roux, Y. Won, G. Archontis, C. Bartels, S. Boresch, *Journal of computational chemistry* **2009**, *30*, 10 1545.

[6] J. Lee, X. Cheng, S. Jo, A. D. MacKerell, J. B. Klauda, W. Im, *Journal of chemical theory and computation* **2016**, *12*, 1 405.

[7] W. G. Hoover, *Physical review A* **1985**, *31*, 3 1695.

[8] M. Parrinello, A. Rahman, *Journal of Applied physics* **1981**, *52*, 12 7182.

[9] B. Hess, H. Bekker, H. J. Berendsen, J. G. Fraaije, *Journal of computational chemistry* **1997**, *18*, 12 1463.

[10] S. Pronk, S. Páll, R. Schulz, P. Larsson, P. Bjelkmar, R. Apostolov, M. R. Shirts, J. C. Smith, P. M. Kasson, D. Van Der Spoel, *Bioinformatics* **2013**, *29*, 7 845.

[11] J. Huang, A. D. MacKerell Jr, *Journal of computational chemistry* **2013**, *34*, 25 2135.

[12] W. L. Jorgensen, J. Chandrasekhar, J. D. Madura, R. W. Impey, M. L. Klein, *The Journal of chemical physics* **1983**, *79*, 2 926.
